# *In situ* gradient nanoprecipitation enables alloys with record-high as-cast strength−ductility synergy

**DOI:** 10.1093/nsr/nwag094

**Published:** 2026-02-10

**Authors:** Peijian Shi, Yi Li, Junfeng Duan, Xin Jiang, Ziyu Peng, Runguang Li, Bodong Tan, Xiaohan Wang, Yiheng Ruan, Baocheng Nie, Bangfei Zhou, Hui Li, Shilei Li, Yunbo Zhong, C T Liu, En (Evan) Ma

**Affiliations:** School of Materials Science and Engineering, State Key Laboratory of Materials for Advanced Nuclear Energy, Shanghai University, Shanghai 200444, China; School of Materials Science and Engineering, State Key Laboratory of Materials for Advanced Nuclear Energy, Shanghai University, Shanghai 200444, China; School of Materials Science and Engineering, State Key Laboratory of Materials for Advanced Nuclear Energy, Shanghai University, Shanghai 200444, China; School of Materials Science and Engineering, State Key Laboratory of Materials for Advanced Nuclear Energy, Shanghai University, Shanghai 200444, China; School of Materials Science and Engineering, State Key Laboratory of Materials for Advanced Nuclear Energy, Shanghai University, Shanghai 200444, China; Institute for Frontier Materials, Deakin University, Geelong 3216, Australia; School of Materials Science and Engineering, State Key Laboratory of Materials for Advanced Nuclear Energy, Shanghai University, Shanghai 200444, China; School of Materials Science and Engineering, State Key Laboratory of Materials for Advanced Nuclear Energy, Shanghai University, Shanghai 200444, China; School of Materials Science and Engineering, State Key Laboratory of Materials for Advanced Nuclear Energy, Shanghai University, Shanghai 200444, China; Department of Materials Science and Engineering, City University of Hong Kong, Hong Kong 999077, China; School of Materials Science and Engineering, State Key Laboratory of Materials for Advanced Nuclear Energy, Shanghai University, Shanghai 200444, China; Laboratory for Microstructures, Shanghai University, Shanghai 200444, China; State Key Laboratory of Advanced Metals and Materials, University of Science and Technology Beijing, Beijing 100083, China; School of Materials Science and Engineering, State Key Laboratory of Materials for Advanced Nuclear Energy, Shanghai University, Shanghai 200444, China; Department of Materials Science and Engineering, City University of Hong Kong, Hong Kong 999077, China; Center for Alloy Innovation and Design (CAID), State Key Laboratory for Mechanical Behavior of Materials, Xi’an Jiaotong University, Xi’an 710049, China

**Keywords:** direct manufacturing, complex-concentrated alloys, *in situ* nanoprecipitation, as-cast, strength−ductility combinations, commercial alloys

## Abstract

Direct manufacturing of alloys through casting is economical, user-friendly and conducive to near-net-shape processing, and therefore of great application interest. However, as-cast alloys are rarely used directly because, without post-cast thermomechanical treatments, the solidification product is susceptible to coarse grains, compositional segregation and particularly unsatisfying precipitates, which tend towards inadequate strength and brittleness. These ubiquitous disadvantages have, for a long time, seriously hindered practical applications. Here, through screening by using thermodynamic calculations and calorimetric monitoring, we successfully landed non-equiatomic NiCoCrAlTaZrB complex-concentrated alloys (CCAs) to realize *in situ* micro-segregation-induced size-gradient L1_2_ nanoprecipitates increasing from dendritic to interdendritic regions. Consequently, without any post-cast treatment, prolific *in situ* nanoprecipitation strengthening renders ultrahigh as-cast yield strength at the gigapascal (GPa) level, while multiscale chemical/structural segregation heterogeneities reinforced by gradient nanoprecipitates result in progressive and persistent hetero-deformation within dendrites, thereby sustaining a high strain-hardening rate of ∼3 GPa and uniform tensile elongation of up to ∼32%. Such robust as-cast strength−ductility synergy not only exceeds all previous as-cast CCAs, but also outperforms all commercial alloys that have already been optimized via post-cast treatments. This advance through exploiting *in situ* size-gradient nanoprecipitation accomplishes a goal that is more practical than the common pursuit for property records, which all require downstream (multistep and/or subtractive) processing that increases production time and expenses, and even raises severe environmental concerns.

## Teaser

Through screening by using thermodynamic calculations and calorimetric monitoring, we successfully landed non-equiatomic NiCoCrAlTaZrB complex-concentrated alloys to realize dense *in situ* gradient nanoprecipitation, thereby leading to unprecedented as-cast strength−ductility synergy.

## INTRODUCTION

The majority of alloy research currently underway in the laboratories around the world aims for unprecedented performance that surpasses established records [1–12]. This never-ending pursuit, however, inevitably demands added processing tricks to optimize properties, making manufacturing more costly. Yet, one could turn around and ask a different question: can we stick with the most straightforward processing route, i.e. the solidification of alloys via simple liquid casting, but design new alloys in such a way that they can beat the best there is, i.e. those that have dominated the market after years of fine-tuning post-cast thermomechanical treatments?

At first glance, the answer to this question seems negative. In the absence of post-cast treatments such as homogenization annealing, cold/hot deformation and aging, inside the ingot, there would be considerable compositional segregation, large grain sizes and few or no precipitates [13–16], which translate into low yield strength. There is also a propensity for brittleness because the sparse precipitates that form during casting have geometric dimensions ranging from tens to hundreds of micrometers and they reside at grain boundaries [17–20]. Such large precipitates not only are ineffective in strengthening, but also cause severe stress concentrations upon loading, resulting in premature failure even prior to yielding.

Post-cast treatments are also essential for most of the complex-concentrated alloys (CCAs), also dubbed as high-entropy alloys (HEAs), that have emerged in recent years [18–26]. Indeed, the reported HEAs with the best strength−ductility combinations also required lengthy high-temperature homogenization annealing (typically > 1200 K for >18 h), as well as costly (intermediate-temperature) forging, cold/hot rolling and subsequent further heat treatments [21–26]. Much like all previous casting alloys, direct-cast CCAs without thermomechanical treatments (regardless of whether they are single-/dual-/multi-phase) also suffer from a coarse-grained, inhomogeneous solidification structure with element segregations accompanying dendritic growth [13–16], resulting in undesirable properties. Specifically, their tensile yield strength can be as low as 150–300 MPa and their tensile elongation to failure can be less than a few percent due to a susceptibility to premature plastic instability [27–29].

However, there will be circumstances for practical engineering purposes in which it is imperative to strategically seek the direct, one-step manufacturing of high-performance alloys, leaving out complex post-cast treatments [13–16]. In addition to thermomechanical processing being too expensive and time-consuming due to long-duration or precisely controlled multistep annealing/aging at different temperatures, near-net-shape processing with 3D morphology control is sometimes also a mandate. While success stories in cutting out post-cast treatments are few and far between [21–29], it is worth noting that some CCAs do seem to bring forth new opportunities. An example is that decent strength−ductility synergy was achieved for as-cast eutectic CCAs [13], in which the highly concentrated alloying elements lead to finely spaced eutectic lamellar structures. These eutectic CCAs, nevertheless, have not yet approached the best strength−ductility previously achieved in commercial/conventional alloys strengthened via post-cast precipitation hardening (see Fig. S1)—the most efficient approach for strength−ductility optimization. The question posed at the beginning of this paper can then be recast into a modified version: can a CCA, via direct casting alone, produce strength−ductility that rivals and even surpasses those of the best commercial alloys known to date (including those that are age-hardened via complex thermomechanical treatments)? At present, this quest remains a formidable challenge.

This paper showcases our innovative success, without resorting to any post-cast treatment, in directly reducing the feature size of casting microstructures to a previously inaccessible nanoscale hierarchy by using phase-diagram calculations and calorimetric monitoring. Specifically, this coupled strategy realizes proficient *in situ* micro-segregation-mediated gradient nanoprecipitation and strengthening in direct-cast CCAs with compositions designed around Ni_40.00_Co_34.35_Cr_17.15_Al_5.00_Ta_3.50_, which offers an ultrahigh as-cast yield strength at the gigapascal (GPa) level. Meanwhile, the direct-cast multiscale structural/chemical segregation heterogeneities, reinforced with *in situ* gradient nanoprecipitates, stepwise spread the plastic flow all over the sample volume by sequentially evoking planar- and cross-slip dislocations as well as nanoscale stacking-fault and microband networks. These previously unattainable merits not only confer high strain hardening (‘intrinsic ductilization’), but also enable hierarchical crack buffering (‘extrinsic ductilization’), thereby considerably prolonging as-cast uniform elongation by up to ∼32%. Consequently, our CCAs achieve surprisingly high as-cast strength−ductility combinations—not only excelling those of all previous cast CCAs, but also surpassing those of all commercial alloys that have the advantage of established/optimized post-cast processing protocols.

## RESULTS AND DISCUSSION

### Towards efficient *in situ* nanoprecipitation

A main roadblock to high as-cast strength−ductility is the difficulty in achieving adequate precipitation hardening during casting, without post treatments. Specifically, the demanding task is to maximize the volume fraction of the spread-out small coherent nanoprecipitates, while minimizing adverse intergranular precipitates, in a single step of simple solidification. To this end, our alloy design begins by tailoring chemical compositions, as discussed in Note S1. On the thermodynamic modeling side, calculations of phase diagrams (CALPHAD) were performed (see examples in Fig. S2). Screening through the thermodynamic predictions and exploiting prior knowledge, the concentrations of multiple precipitate-forming elements were strategically increased while the contents of matrix-forming elements were reduced, so as to increase the thermodynamic driving force for precipitates and hence their *in situ* population density during casting. From the standpoint of phase-transformation theory, our idea is to find an alloy for which the nose of the *C* curve for precipitate nucleation in the time–temperature–transformation diagram is located at an unusually high temperature and short incubation time, such that copious L1_2_ nanoprecipitates can nucleate and grow with a fine dispersion and a high volume fraction, all within the short time span of conventional casting. Experimental screening was carefully carried out by using differential scanning calorimetry (DSC; see Methods) at a cooling rate of ∼50°C/min, not far from those (70–150°C/min) experienced in our copper-mold casting facility. For the two eventually selected chemical compositions (in atomic %, at.%) of Ni_40.0_Co_34.3_Cr_17.2_Al_5.0_Ta_3.5_ (referred to as A5T3 hereafter) and Ni_39.8_Co_34.1_Cr_17.1_Al_5.0_Ta_4.0_ (A5T4), with trace amounts of B (∼0.006 wt%) and Zr (∼0.10 wt%) added to promote boundary cohesion and grain refinement (Fig. S3), the exothermic peak corresponding to L1_2_ formation is clearly observed in the DSC trace (Fig. S4a). The key here is the deliberately increased contents of Al, Ta and Ni. The former two are strong L1_2_-forming elements, while the latter is the main constituent element in L1_2_. This design is expected to increase the free-energy reduction driving the precipitation of the Ni_3_(Ta, Al)-type L1_2_ phase and decrease the interfacial energy between the precipitate and the matrix (evidence for coherent precipitation will be presented later and discussed in Notes S1 and S2), both lowering the nucleation barrier and incubation time to encourage the *in situ* precipitation of L1_2_ during casting. To see the difference from normal age-hardened alloys, a representative DSC curve for the latter is included in Fig. S4b for comparison. There, the phase-transformation peak to L1_2_ is absent, i.e. too weak to be detectable.

The specially designed CCA is therefore extraordinary in terms of accelerated L1_2_ precipitation. The DSC trace (Fig. S4a) reveals the nucleation burst over a narrow temperature range of 915–1035°C. Such precipitation temperatures are much higher than those used in conventional precipitation hardening via long aging treatments [30–32]. Apparently, in our alloy, the solidified supersaturated solid solution becomes overly supersaturated at relatively high temperatures, at which the atomic diffusion kinetics remain fast. In other words, thermodynamically, the solution has a high driving force to decompose and, kinetics-wise, the L1_2_ particles have no problem in precipitating out at adequate rates of nucleation and growth. As a result, abundant precipitates are able to form well before cooling to low temperatures, at which the phase transformation turns sluggish. Post aging is hence no longer a must-have for massive precipitation hardening. Thereafter, the quickly descending temperature during casting limits the coarsening of the precipitates.

### As-cast CCAs with *in situ* gradient nanoprecipitation

**Figure 1.**
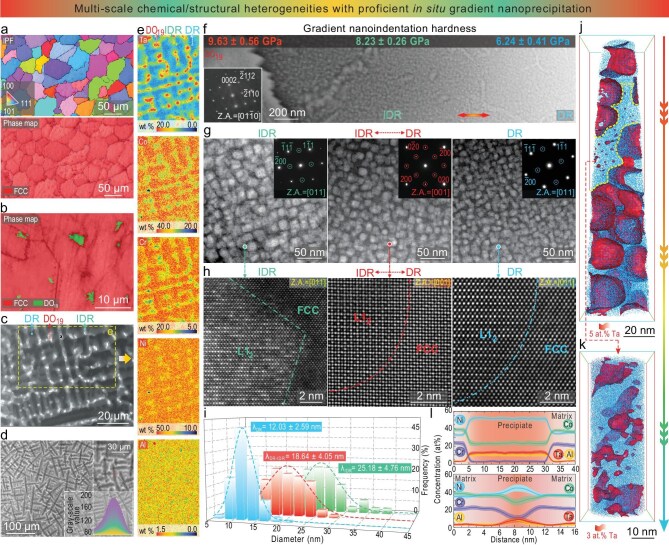
As-cast CCAs with *in situ* gradient nanoprecipitates. (a) EBSD IPF and the corresponding phase map. (b) Enlarged EBSD phase map. (c) Scanning-electron microscopy (SEM) image of D0_19_, DRs and IDRs. (d) 3D CT reconstruction of IDRs as well as DRs from the core to periphery. (e) EPMA–WDS images revealing the element distribution of Al, Co, Cr, Ni and Ta in D0_19_, IDR and DR regions. (f) Scanning TEM (STEM) image of *in situ* gradient nanoprecipitates embedded in DRs and IDRs, as well as D0_19_ particles and its selected-area electron diffraction (SAED), inset, with gradient hardness values. (g) Three STEM images revealing dense L1_2_ nanoprecipitates with different appearances and gradually reduced sizes from the IDR to the DR. Three insets are the corresponding SAED patterns, where super-lattice spots are circled. (h) Three atomic-scale high-angle annular dark field–STEM (HAADF–STEM) images revealing coherent L1_2_ nanoprecipitates from the IDR to the DR. (i) Corresponding size distribution of nanoprecipitates from the DR to the IDR. (j) 3D-atom probe tomography (3D-APT) reconstruction of the FCC matrix and L1_2_ nanoprecipitates in the IDR. The yellow dotted lines in (j) enclose these smaller L1_2_ nanoprecipitates, which are further enlarged in (k). (l) Corresponding 1D compositional profiles of primary larger L1_2_ (upper) and smaller L1_2_ (under) nanoprecipitates.

Figure 1 shows multiscale characterizations of our directly solidified CCAs. The electron-backscatter diffraction (EBSD) inverse pole figure (IPF) and phase map (Fig. 1a and b) reveal that the as-cast CCAs have an average grain size of 54.2 ± 10.6 μm. The grain matrix is a face-centered cubic (FCC)-structured phase containing some sparsely spotted micrometer-scale intragranular precipitates (3.5 ± 0.6 μm). The latter, distributed mainly inside the grains, are hard, brittle intermetallics, with an ordered hexagonal closely packed, D0_19_, crystal structure, as identified by the EBSD phase map (Fig. 1b) and selected-area electron diffraction (SAED; inset, Fig. 1f). The scanning-electron microscopy (SEM) image (Fig. 1c) further reveals that these coarse grains are composed of highly branched dendrites. Complementary 3D computerized tomography (CT; Fig. 1d) unveils the branched dendritic and interdendritic regions (abbreviated as DR and IDR, respectively) in a complex 3D network, which, together with D0_19_ precipitates embedded in the IDRs (Fig. 1c), effectively subdivides these coarse grains. The formation of dendrites stems from the relatively high cooling rate (70–150°C/s). The latter during casting induces large constitutional supercooling, whereby rapid and non-equilibrium dendrite solidification begins (Note S3).

To disclose finer substructures, focused ion-beam sectioning was deployed for site-specific sampling (see Methods), followed by transmission-electron microscopy (TEM) examinations. Long-scale scanning TEM (STEM) probing (Fig. 1f, g and i) reveals widespread nanoscale precipitates, manifesting as a gradient with an increasing size from DRs to IDRs. All these gradient nanoprecipitates have an L1_2_-type crystal structure and are coherent with the FCC-structured DRs and IDRs, as evidenced by SAED (insets, Fig. 1g) and atomic-scale high-angle annular dark field–STEM (HAADF–STEM; Fig. 1h) images. No other types of precipitations are detected through synchrotron high-energy X-ray diffraction (XRD; Fig. S5a). The L1_2_ precipitates in the IDRs display a larger average size and volume fraction of 25.18 ± 4.76 nm and 48 ± 5 vol.%, respectively, compared with those in the DRs (12.03 ± 2.59 nm and 29 ± 6 vol.%) (Fig. 1i). Besides, the precipitates in the IDRs have partially faceted and planar interfaces, whereas those in the DRs display a more spheroidal morphology (Fig. 1g and h). The lattice misfits between the FCC matrix and the L1_2_ precipitate were calculated by using lattice parameters acquired from these atomic-scale HAADF–STEM images (Fig. 1h) to be ∼0.36% and ∼0.58% in the DRs and IDRs, respectively. In comparison to the IDRs, the smaller lattice misfit in the DRs suggests a lower precipitate–matrix interfacial energy, thereby favoring the observed spherical morphology of nanoprecipitates and lowering the nucleation barrier. Regardless of their size, location and morphology, all these nanoprecipitates (as high as 38 ± 5 vol.% in total) are formed directly during solidification, so they will be termed ‘*in situ* nanoprecipitates’ in this paper to distinguish them from those *ex situ* nanoprecipitates emerging during the post-cast aging. Because of the fast cooling rate during casting, the L1_2_ phase could not be fully precipitated, so its volume fraction was less than the prediction given by the equilibrium thermodynamic calculation (Fig. S2).

Next, the origin of the spatial gradient in the size distribution of the nanoprecipitates, i.e. the *in situ* gradient nanoprecipitation, was explored (Fig. 1f–i). To this end, long-range elemental partitioning was measured by using electron probe micro-analysis with X-ray wavelength dispersive spectroscopy (EPMA–WDS). As revealed in Fig. 1e, obvious compositional variations exist among the DRs, IDRs and D0_19_ due to the rapid solidification-induced dendrite micro-segregation. First, the DRs are enriched with Co and Cr elements, and the IDRs and D0_19_ are enriched with Ta. Al and Ni elements exhibit a relatively homogeneous distribution in both the DRs and the IDRs. Second, the color-intensity bars of elements in the EPMA–WDS indicate a compositional gradient between the DRs and IDRs, spreading over a wide distance (5–20 μm). This gradient inhomogeneity hence develops diffused DR–IDR interfaces, while forming *in situ* gradient nanoprecipitates (Fig. 1f–i). The latter correspondingly produce an obvious gradient in hardness [33], increasing from 6.24 ± 0.41 GPa (in the DRs) to 9.63 ± 0.56 GPa (in the IDRs), as quantified by using multiple nanoindentation tests (see Methods, inset of Fig. 1f and Fig. S6). Third, the 3D CT presented in Fig. 1d shows a marked contrast due to elemental partitioning. Besides, a gradual transition in greyscale is detected from the core of the DR to its periphery (insets, Fig. 1d) due to the compositional gradient along its radial direction. Thus, the 3D CT reveals a solidified structure characterized by three spatially distinct zones—(i) the core and (ii) periphery of the DR and (iii) the IDR channel. Fourth, near-atomic-scale elemental partitioning between the FCC matrix and the L1_2_ precipitate is resolved by using 3D-atom probe tomography (APT; Fig. 1j and k). Figure 1l (upper) shows the corresponding 1D compositional profiles of the IDRs: the Ni, Ta and Al elements strongly partition into L1_2_ nanoprecipitates, in which Co is moderately depleted and Cr is significantly depleted. Fifth, compositional partitioning was also detected in the DRs (Fig. S7), although not with the same trends as those in the IDRs. Sixth, interestingly, finer L1_2_ precipitates (<6.5 nm; zoomed-in image in Fig. 1k and Fig. S8) are detected in many locations of the leftover FCC matrix of the IDRs. This case further indicates that our screening-secured CCA composition is characterized by strong precipitating propensity (Note S4), avoiding the precipitate-free zones that are often wide and prone to strain localization and damage. Figure 1l (lower panel) shows that these finer L1_2_ precipitates have different chemical compositions compared with the predominant larger precipitates in the IDRs. In sum, due to the profuse chemical heterogeneities underlying dendrite micro-segregation, widespread *in situ* gradient nanoprecipitates are formed in our CCAs, in the absence of any post-cast treatment. Thus, such microstructure traits containing *in situ* gradient nanoprecipitates exhibit significant differences from those in a recent study [17] (Note S5).

### Record-high as-cast strength−ductility

**Figure 2.**
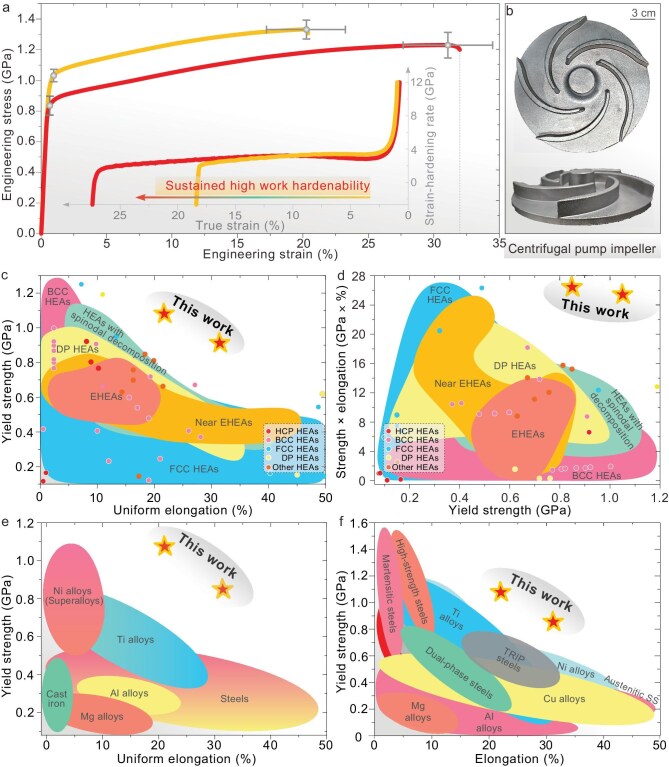
Record-high as-cast strength−ductility combinations enabled by *in situ* gradient nanoprecipitates. (a) Tensile stress–strain curves of two as-cast CCAs: A5T3 (red) and A5T4 (yellow). The inset displays their sustained strain-hardening rates. Repeatable properties under larger sample thickness are indicated in Fig. S25. (b) Cast pump impeller. (c, d) Two property-comparison maps of (c) yield strength versus uniform elongation and (d) ‘yield strength × uniform elongation’ versus yield strength, compared with all previous advanced as-cast CCAs. DP HEAs, Near EHEAs, HCP HEAs and BCC HEAs refer to dual-phase HEAs, near eutectic HEAs, hexagonal close-packed HEAs and body-centered cubic HEAs, respectively. Detailed data and associated references are provided in Fig. S24 and Table S3. (e) Yield strength versus uniform elongation, compared with commercially available conventional as-cast alloys and steels. (f) Yield strength versus total elongation to failure, compared with those commercial alloys and steels that have been processed/optimized via complex thermomechanical treatments.

Figure 2a reveals that such gradient structural/chemical heterogeneities led to surprisingly high as-cast strength−ductility combinations, while simultaneously delivering a remarkable outcome on multiple fronts. First, the ultrahigh (GPa-level) as-cast yield strength of 0.84–1.05 GPa can be achieved directly in our CCAs, even though the as-cast grain size is >50 μm. Second, large uniform elongation, of ≤20.5–31.5%, can be simultaneously obtained at a GPa-level yield strength. It is desirable to achieve high uniform elongation (far more than 10%) for high-strength cast alloys (see Note S6). Third, as indicated by the high uniform elongation, the brittle large intermetallic (D0_19_) particles did not cause the severe ductility loss that is commonly seen in previous precipitation-hardened CCA materials [34]. Fourth, apart from the high yield strength, our as-cast CCAs also feature a high ultimate tensile strength of 1.24–1.33 GPa. This amounts to a considerable strength difference of 280–400 MPa (= ultimate strength – yield strength); such pronounced strain hardening is advantageous in structural applications to guarantee a large safety margin before fracture and is useful for uniform shaping and forming in processing. Last but not least, these as-cast properties are competitive with those of many previous CCAs that have been optimally age-hardened [35]; again, the latter required costly and/or time-consuming thermomechanical processing.

To more directly highlight the achieved superior as-cast properties, an Ashby plot was first used to compare them with those of all previously reported cast single-/dual-/multi-phase CCAs. As displayed in Fig. 2c, our properties unequivocally stand out from all previous as-cast strength−ductility profiles. A further comparison can be made in the form of Fig. 2d, in which the strength−ductility product, obtained through ‘yield strength × uniform elongation’, again sets our as-cast CCAs apart from all previous as-cast CCAs. This further confirms/evidences the record-high as-cast mechanical properties achieved by our designed CCAs.

A broader performance comparison is then made between our as-cast CCAs and various grades of commercially available alloys and steels, regardless of their alloying species, structures, phase components and post-cast treatments (from American Society for Metals and American Society for Testing and Materials Handbooks; Note S7). First, our as-cast CCAs are far superior to all commercial metallic materials when they are prepared in the as-cast form (Fig. 2e). Second, and more importantly, our as-cast CCAs even outperform all commercial alloys that have been through carefully designed post-cast processing—these are benchmarks in the market (Fig. 2f, Fig. S9a–f and Note S7).

As such, the strength−ductility combinations of our as-cast CCAs having *in situ* gradient nanoprecipitates that clearly excel over all previously reported cast CCAs and commercial alloys; the latter always undergo complex, costly and/or time-intensive post-cast treatments for satisfying the application-required high performance. Thus, compared with commercial alloys, not requiring any downstream processing, but having higher strength−ductility combinations, makes our as-cast CCAs highly promising in practical applications across multiple industries, particularly when considering once again that the applied method—casting—is inexpensive, user-friendly and conducive to near-net-shape manufacturing. Of course, the mechanical properties of this studied CCA can be further enhanced by thermomechanical processing and structure refinement [33–37].

### *In situ* gradient nanoprecipitates enhance properties

Next, we discuss the micro-mechanisms underpinning the record-high as-cast strength−ductility combinations. Compared with the precipitate-free Ni_43.75_Co_37.50_Cr_18.75_ baseline CCA, our present as-cast CCA has a yield strength that is exceptionally high, by a whopping ∼700 MPa margin (see Fig. S10). This clearly points to the significance of *in situ* gradient nanoprecipitation and strengthening. Quantitative calculations suggest that gradient L1_2_ nanoprecipitates contribute to a remarkable strength increase of ∼535 MPa, while the remaining part of the yield strength mainly comes from solid-solution strengthening (Note S8).

**Figure 3.**
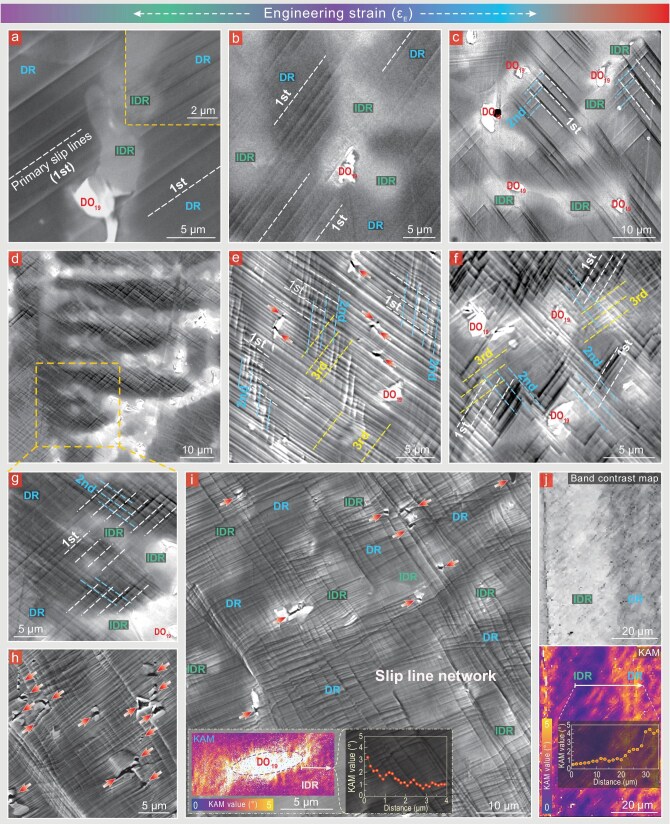
Multiscale correlated microcopy characterizations at different strain stages. (a–i) SEM characterizations of dynamic slip-line evolution with increasing tensile strain, in DRs, IDRs and brittle D0_19_ particles. Primary, secondary and tertiary slip lines (1st, 2nd and 3rd) are marked by dotted lines of different colors. Microcracks in/around the D0_19_ particles are indicated by red arrows. The inset in (i) shows the kernel average misorientation (KAM) map, revealing the gradually increased KAM values when approaching the D0_19_ particle. (j) Band contrast map and KAM map, revealing the gradually decreased KAM values from the DR to the IDR.

**Figure 4.**
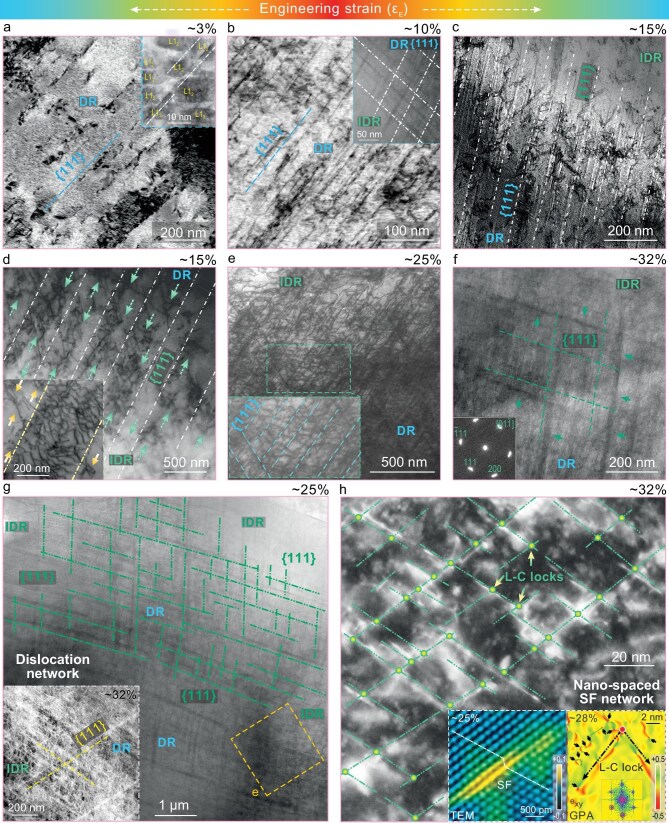
Intense multimode defect interactions and dislocation accumulation operating stepwise from the DR to the IDR. (a) Transmission-electron microscopy (TEM) image revealing planar-slip of {111} dislocations in the DR. The scanning TEM (STEM) inset shows dislocation shearing for L1_2_ nanoprecipitates (marked by blue dotted lines). (b) STEM image revealing densely planar-slip {111} dislocations in the DR. The STEM inset shows bidirectional planar dislocations, which are obvious in the DR, but gradually absent when approaching the IDR. (c) STEM image revealing densely planar {111} dislocations (marked by blue dotted lines) and frequent dislocation cross-slip (among planar {111} dislocations). The cross-slip prevails around diffused DR/IDR interfaces and further details are shown in the inset (d). (d) STEM image revealing intensive dislocation interactions (marked by green arrows) within {111} slip bands, which are further displayed in the enlarged STEM inset (in which yellow arrows mark cross-slip dislocations, away from {111} slip bands). (e) Enlarged STEM image for (g) (see orange frame) revealing dislocation network and cross-slip, as further shown in the zoomed-in inset. (f and g) STEM images, together with low-angle annular dark field–STEM (LAADF–STEM) inset (g), revealing bi-/multidirectional planar-slip and widespread cross-slip as dislocations proliferate, thereby making full use of the undeformed volume, while promoting dense and intense dislocation accumulation. The SAED inset (f) indicates no twinning and phase transformation. (g) STEM image revealing planar-slip dislocation network (marked by green lines). (h) ADF–STEM images revealing gradually formed SFs (see left atomic-scale TEM inset), L–C locks [right geometric phase analysis (GPA) inset] and nano-spaced SF networks with straining (25%–32%).

The sustained high strain-hardenability, as revealed in the inset of Fig. 2a, should account for the large tensile ductility, especially the impressive uniform elongation as high as ∼31.5%, along with a robust as-cast tensile strength of 1.24–1.33 GPa. With the aim of deciphering the origin of this strain hardening at high flow stresses, we next monitored how the plastic flow is mediated at different strain stages in A5T3. Upon early straining (∼3%), the SEM results of the pre-polished surface display a few slip lines (Fig. 3a), due to dislocation planar-slip on the {111} planes (see TEM image; Fig. 4a). Planar-slip is well known to result from glide-plane softening, which happens here because of the partial destruction of the shearable L1_2_ nanoprecipitates as the repeated slipping dislocations open up easier pathways for the ensuing dislocations to follow suit (inset, Fig. 4a). Interestingly, these slip lines are mostly distributed in the DRs even upon further straining (∼6%; Fig. 3b) due to their relatively low hardness (inset, Fig. 1f). With increasing strains (6%–10%), the slip lines run/operate successively from the core to the periphery of the DRs (Fig. 3b and c) due to a gradient hardness difference. Meanwhile, the spacing between the primary slip lines (labeled as ***1st***) is gradually reduced (due to the fresh slip lines in the free space between the initially existing ones), along with newly activated secondary slip lines (***2nd***; to alleviate stress concentrations) (Fig. 3b and c). These progressive plastic flows spread all over the DRs. Also note that the planar-dislocation spacing is even refined to 20–30 nm in some regions (Fig. 4b). Such dense, intersecting planar-slip leads to a high frequency and density of local short-range interplay between dislocations and between dislocations and nanoprecipitates. As such, these short-range interactions greatly contribute to forest-dislocation hardening, creating a persistent strain hardening at true strains of <10% (inset, Fig. 2a).

After a medium strain (∼14%), it was observed that massive slip lines get transmitted successively from the readily deformable DRs into adjoining IDRs (Fig. 3d and g), thanks to the hardness gradient (enabled by *in situ* gradient nanoprecipitates) and the same crystal structure between the DRs and IDRs. The built-in strain gradients necessitate additional geometrically necessary dislocations (GNDs), as confirmed by the gradually increased EBSD kernel average misorientation (KAM) values (Fig. 3j), that contribute to non-homogeneous deformation and hence back-stress hardening. Moreover, it was noticed that the degree of slip transfer is highly varied; many slip lines terminate inside harder IDRs (Fig. 3d and g). Nanometer-resolved STEM reveals that this strong resistance enables frequent dislocation cross-slip (Fig. 4c), away from the primary {111} slip bands (marked by yellow arrows; inset, Fig. 4d). Besides, under the strong resistance, widespread interactions of dislocations were detected within their initially operating {111} slip bands (Fig. 4d and its enlarged inset). Thus, these slip bands do not undergo outright softening that would trigger plastic instability, but rather harden with proliferating dislocation activities. Also note that extensive cross-slip (Fig. 4c and d) makes better use of the undeformed volume, markedly promoting the deformation delocalization into the regions between those already-formed slip bands. At a larger strain (∼20%), more far-ranging slip lines with more pronounced slip-transfer activities were observed in Fig. 3e. (i) The DRs exhibit a higher density and frequency of slip lines and short-range interactions among them, as well as the activation of higher-order slip lines (***3rd***). (ii) Massive primary slip lines pass through harder IDRs, while some secondary ones gradually penetrate into the IDRs. These diversified scenarios hence continue progressive plastic deformation, whereas the obvious differences in the slip-line density seen in Fig. 3e highlight the persistent inhomogeneous deformation. Again, these contribute to simultaneous forest-dislocation hardening and back-stress hardening, thereby collectively preserving the high strain-hardening rate over the true-strain stage of 10%–18% (inset, Fig. 2a).

Upon further straining (∼25%), abundant primary and secondary slip lines gradually penetrate the IDRs (Fig. 3f). Yet, some adjoining harder D0_19_ particles tend to slow down and block their movement. This pre-empts outright softening of the slip bands and instead develops long-range, GND-mediated strain gradients around D0_19_ particles (as revealed by the gradient EBSD–KAM distribution; inset, Fig. 3i), also adding back-stress hardening. Meanwhile, it was observed that these slip lines from the IDRs, without the obstruction of D0_19_, are frequently interlaced to interact with other slip lines from other dendrite arms and vice versa (Fig. 3h and i). Thus, an interlocking deformation network mediated by slip lines of varied lengths is built up step by step—dynamically refining the substructure down to the ∼2-μm scale (Fig. 3i). Further STEM probing (Fig. 4g) reveals a finer, nanometer-spaced planar-dislocation network prevailing underlying these interlocking slip lines, thereby enabling higher-degree substructure refinement with more profuse dislocation entanglement and accumulation. Nanoscale deformation details are further revealed through complementary STEM and low-angle ADF (LAADF) STEM probing (Fig. 4e and its zoomed-in inset, inset of Fig. 4g and Fig. S11). Exceedingly extensive intersections of primary and secondary planar dislocations as well as frequent dislocation cross-slip are observed, creating dislocation tangles all over the DRs and IDRs, especially in the latter. At the final stage (25%–32% strain), stacking faults (SFs), Lomer–Cottrell (L–C) locks and even nano-spaced SF networks are gradually accumulating (Fig. 4h and its two insets). These behaviors also promote dislocation trapping [evidenced by the black arrows in the geometric phase analysis (GPA), inset of Fig. 4h] and contribute to dynamic refinement and strain hardening. Such deformation mechanisms are also expected to impart high fatigue resistance [37].

Unexpectedly, the strong shearing forces offered by primary slip trigger the internal breakage of some D0_19_ particles (see red arrows; Fig. 3e), including those rather large D0_19_ particles when reaching higher strains (25%–32%; Fig. 3h and i). Accordingly, the cracking path proceeds mostly along the direction of the slip lines, rather than the phase interfaces (which instigate premature failure). As such, the stress concentrations are relieved, without interface debonding. Instead, over a wide strain range (20%–32%), the incipient microcracks dynamically adjust their morphology (Fig. 3e, h and i). In addition, the high and steady strain-hardening ability of the IDRs confers intense crack buffering and arresting around the D0_19_ particles, such that, after incipient damage nucleation, no large (secondary) cracks were observed (Fig. 3i). These incidents pre-empt the interface debonding and crack-related premature failure, which often occur in previous brittle intermetallic-containing high-strength CCAs [31,32,34,35].

Finally, loading–unloading–reloading experiments were conducted to quantitatively monitor the evolution of the back and effective stresses (see Methods and Fig. S12 for separating out the two contributions). At the early strain stage (<10%), a large increase was observed in the short-range effective stress, indicative of forest-dislocation hardening, the contribution of which is obviously higher than that of the long-range GND-assisted back stress. This matches well with our microstructure observations in this early deformation stage (Figs 3a–c and 4a, b), in which the plastic deformation is dominated by widespread local dislocation shearing for nanoprecipitates as well as short-range interactions between the planar {111} dislocations. Upon further straining (10%–20%), the DR/IDR non-homogeneous deformation increases the back stress to slightly surpass the effective stress (Fig. S12b). Subsequently, the small difference between the two stresses persists over a rather wide strain range (20%–32%). This can be due to a dynamic balance between the diversified short-range interactions (i.e. effective stress) and the persistent non-homogeneous deformations (back stress) discussed earlier. Note that, at the final stage (25%–32% strain), the formation of SFs, L–C locks and nano-spaced SF networks (Fig. 4h) together with some thin microbands (green arrows; Fig. 4f and its inset) dynamically reinforced both the structural heterogeneity and the ensuing non-homogeneous plastic deformation [38–41]. This enhances the back-stress hardening to balance out the decrease in back stress caused by the weakened non-homogeneous plastic flow. Further high-energy XRD and SAED results are reported in Figs S5b and S13, respectively. No new diffraction peaks and spots were detected at the fracture, indicating that the record-high as-cast tensile properties of our CCAs are not caused by the commonly found TWIP (e.g. nanotwins) and TRIP (phase transformations) [42,43] mechanisms, but are rather a result of multilevel structural/chemical heterogeneities reinforced by *in situ* gradient nanoprecipitates [40,41]. The latter, as schematically illustrated in Fig. S14, help to sequentially spread/diversify the plastic flow via multiple coupled defect modes at high flow stresses. A counter example used to further confirm this is given in Note S9 and Figs S15–S19.

## CONCLUDING REMARKS

**Figure 5.**
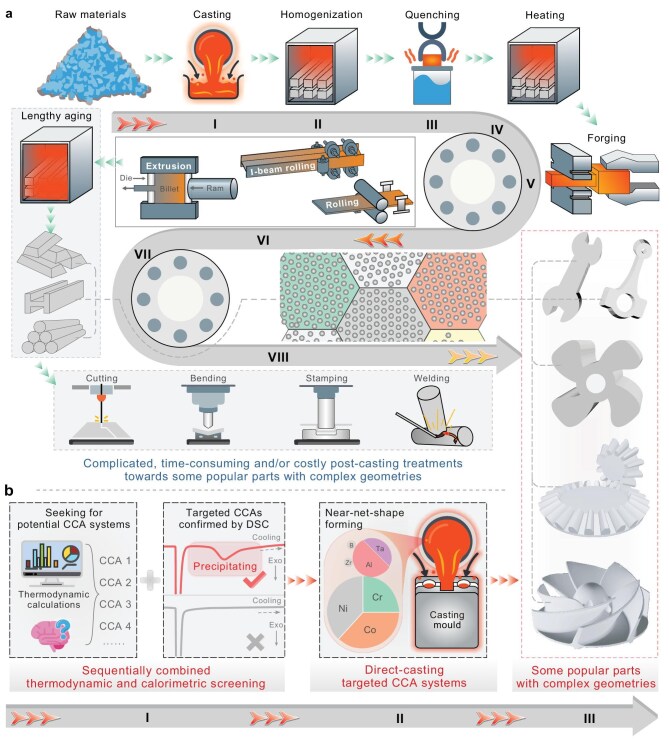
Schematic diagrams showing different manufacturing procedures, suggesting the economic, eco-friendly and technological significance of our present work (b) compared with traditional manufacturing (a). (a) Complicated, time-consuming and/or costly post-casting treatments towards high-performance parts with complex geometries. (b) Fast and direct manufacturing of complex-shaped parts with superior properties, following a sequentially combined thermodynamic and calorimetric screening strategy. Our study (b) towards direct-cast superior properties, especially casting some key and popular parts with complex geometries, can greatly reduce the amount of raw materials needed and shorten the manufacturing process, as well as being an important advancement that is different from the often relentless pursuit for unprecedented properties, which all required downstream processing that increases (being often multistep and subtractive) production time, expenses and pollution, as well as greenhouse gas emissions (a).

Before closing, we emphasize once more that, through *in situ* gradient nanoprecipitation, our achieved record-high as-cast properties (even superior anti-corrosion performance, as seen in Fig. S20) do not require any downstream processing treatment, making the new as-cast alloys highly promising for practical applications. The superb casting fluidity of the new alloys is demonstrated in Fig. S21 and was successfully exploited to cast broadly useful machine parts (see Fig. 2b and Fig. S22 and its legend). Also note that casting is an economic, user-friendly method that is extensively used to manufacture a variety of products, including those with complex geometries. In particular, their near-net-shape-forming capability can greatly reduce the amount of raw materials needed and shorten the manufacturing process compared with subtractive processes (as schematically illustrated in Fig. 5), resulting in high productivity as well as low cost, including a reduced environmental impact (e.g. greenhouse gas emissions). Furthermore, we extended our composition screening strategy and successfully landed as-cast strength−ductility synergy in other alloys, including, but not limited to, Ni_40_Co_34_Cr_17_Al_5_Ta_5_, Ni_35_Co_37_Cr_18_Al_5_Ti_5_, Ni_35_Co_33_Cr_17_Al_5_Ti_3_Nb_2_ and Ni_40.7_Co_20.6_Cr_12.2_Fe_11.5_Al_7.8_Ta_7.2_ (Fig. S23). Finally, as an outlook, one can integrate machine learning into the design of higher-performance as-cast CCAs and further extend our alloy design by combining it with Scheil simulations in Thermo-Calc software to track the segregation of alloying elements during casting.

## Supplementary Material

nwag094_Supplemental_File
