# Supplementary material for: In situ gradient nanoprecipitation enables alloys with record-high as-cast strength−ductility synergy
Source: Natl Sci Rev. 2026 Feb 10;13(9):nwag094. doi: 10.1093/nsr/nwag094 (PMC13170801; doi:10.1093/nsr/nwag094)
Supplement: nwag094_Supplemental_File [file nwag094_supplemental_file.pdf]

Article

In situ gradient nanoprecipitation enables alloys with record-high as-cast strength−ductility synergy

Authors: Peijian Shi, Yi Li, Junfeng Duan, Xin Jiang, Ziyu Peng, Runguang Li, Bodong Tan, Xiaohan Wang, Yiheng Ruan, Baocheng Nie, Bangfei Zhou, Hui Li, Shilei Li, Yunbo Zhong, C.T. Liu & En (Evan) Ma

This supplementary data includes:

Methods

Notes S1 to S9

Figs S1 to S25

Tables S1 to S4

References 44 to 68

References (1~207) for Tables S3 and S4

Methods

Sample preparation. The CCA ingots with nominal compositions of $Ni_{40.0}Co_{34.3}$-$Cr_{17.2}Al_{5.0}Ta_{3.5}$ (A5T3) and $Ni_{39.8}Co_{34.1}Cr_{17.1}Al_{5.0}Ta_{4.0}$ (A5T4) (in atomic percent, at.%) were synthesized from pure metals (> 99.9 in weight percent, wt.%) via melting using a vacuum arc furnace. All ingots with minor additions of B and Zr elements (~0.006 wt.% and ~0.10 wt.%, respectively) were re-melted at least five times to ensure the chemical homogeneity and then cast into water cooled copper molds with a size of 80 mm (length) × 40 mm (width) × 6 mm (thickness). The actual chemical compositions of both CCAs were analyzed using inductively-coupled plasma atomic emission spectroscopy (ICP-AES) and summarized in the following table:

Table S1: Actual chemical compositions

at%	Ni	Co	Cr	Al	Ta	Zr	B
A5T3	Bal.	34.28	17.84	4.68	3.56	0.07	0.03
A5T4	Bal.	33.89	18.25	4.65	4.03	0.07	0.03

For comparison, some CCA ingots were homogenized for 12 h at 1200 ℃ in high-purity argon gas flow followed by water-quenching to eliminate dendrites. Then these homogenized CCAs were aged for 4 h at 800 ℃.

The CCA ingots in Fig. S23 were also prepared using the same equipment and method.

Repeatable microstructures and mechanical properties under a larger sample thickness were demonstrated in Fig. S25.

SEM, EPMA and EBSD observations. The microstructure was characterized using a field-emission scanning electron microscope (FEI Nova 450, USA) in backscattered electron (BSE) and secondary electron (SE) modes, equipped with an energy dispersive spectrometer (EDS) for chemical composition analysis. Further component analysis was conducted on a X-ray wavelength dispersive spectroscopy (EPMA-WDS) (JEOL JXA iHP200F, Japan). More detailed microstructure was conducted using Electron backscatter diffraction (EBSD) in a Zeiss field emission scanning electron microscope (Carl Zeiss Gemini 560, Germany) with a Symmetry S2 EBSD detector. Associated EBSD analysis was conducted using Oxford AztecCrystal 2.2 software[44]. These specimens for the SEM, EPMA observation are semi-automatically ground and polished with a diamond paste to achieve a sub-micron finish. The particle diameters for the diamond paste were 2.5 μm and 1.0 μm. Silica particles with a diameter of 0.04 μm were used for the final mirror polishing. These specimens for EBSD analysis were subsequently electrochemically polished using a 6% perchloric acid + 30% n-butyl alcohol + 64% methyl alcohol solution (vol%) at a direct voltage of 30 V at -30 ℃.

TEM, STEM, LAADF-STEM, and HAADF-STEM characterizations. Transmission electron microscopy

(TEM), scanning TEM (STEM), and high-angle annular dark-field scanning TEM (HAADF-STEM) analyses were conducted in a JEOL-2100F and JEM-F200 TEM operating at 200~300 kV. For low-angle annular dark-field (LAADF) imaging, a probe semi-convergence angle of 17 mrad and inner and outer semi-collection angles from 14 mrad to 63 mrad were used. Ultra-high-resolution HAADF-STEM images were acquired using a probe Cs-corrected transmission electron microscope (JEM-ARM300F, Japan). TEM specimens were first mechanically ground to ~30 μm thickness and then twin-jet electropolished using a mixture of 90% ethanol and 10% perchloric acid (vol%) at -30 ℃. Some high-quality TEM observations and reconfirmation were also made using samples prepared via FEI HONGKONG 600i dual-beam focused ion beam (FIB) instrument in multiple steps starting with 30 kV Ga^+ ions down to 5 kV ions for reducing surface damage induced by the high energy Ga ions. The sample was tilted 52 ±1.1°, and the Ga ions strike perpendicular to the sample surface. For the obtained TEM sample, the Ga ion energy is reduced to 2 kV for final polishing with uniform sample thinning. FFT analysis was performed by using the DigitalMicrograph software (Gatan Inc., USA). The lattice misfit ($K^{\gamma/\gamma'}$) was calculated by the equation of $K^{\gamma/\gamma'}=2(C_{L12} - C_{matrix}) / (C_{L12} + C_{matrix})$, in which the C refers to the lattice parameter of each phase[45].

3D Atom probe tomography. Near-atomic scale elemental information was gathered using a LEAP™ 5000X HR (CAMECA) under a high vacuum of 2×10^{-11} Torr. Atom probe tomography (APT) experiment was run in the laser mode with a specimen temperature of 60 K, a pulse rate of 125 kHz, laser energy of 40 pJ, and a detection rate of 0.5%. The APT results were reconstructed and analyzed employing the CAMECA integrated visualization and analysis software (IVAS 3.8.6). Needle-shaped samples for APT characterizations were prepared via FEI HONGKONG 600i FIB.

Element partitioning coefficient. To quantitatively assess the partitioning behavior between $L1_2$-phase and FCC-matrix in alloying elements, the partitioning coefficient is given by:

$$K_i^{L1_2/FCC} = \frac{C_i^{L1_2}}{C_i^{FCC}} \qquad (1)$$

where $C_i^{L1_2}$ and C_i^{FCC} are the atomic concentration of alloying element *i* in $L1_2$-phase and FCC-matrix, respectively.

Synchrotron-based high-energy X-ray diffraction. *In situ* high-energy X-ray diffraction measurements were carried out on the t the beamline BL16B of the Shanghai Synchrotron Radiation Facility (SSRF). Dimensions of the tensile specimen in the gage part were ~10.16 mm (length) × ~3.18 mm (width) × ~0.80 mm (thickness). During tensile loading, a monochromatic X-ray beam ($\lambda = 0.124$ nm) and beam size of 139 μm (height) × 139 μm (width) was used. A 2-D detector was placed ~1700 mm behind the tensile sample to record the scattering intensity.

Nanoindentation test. Nanoindentation tests were used to determine the hardness of the as-cast samples before and after tensile test via a MicroMaterials Platform 3 nanoindenter with a Berkovich indenter tip.

During each indentation, the load was increased linearly from 0 mN to 3 mN over a period of 5 s, kept constant for 3 s, and unloaded to 0 mN over a period of 5 s. 49 nanoindentations were made in each specimen in a 5 × 7 grid and the spacing between each indentation is 10 μm.

Quasi-static tensile test at room temperature. Dogbone-shaped tensile samples with a cross-section of 3.2 × 1.0 mm^2 and a gauge length of 13.5 mm were cut from the as-cast CCAs using wire electro-discharge machining. Room-temperature tensile tests were conducted in an MTS Exceed E44 machine with an initial strain rate of ~1 × 10^{-3} s^{-1}. Before testing, all sides of the gauge section were carefully ground to a 2000-grit finish using SiC grinding papers. The tensile tests were repeated at least five times for each alloy condition to ensure reproducibility. The strains were measured using a 10-mm extensometer. The yield strength reported is taken at the 0.2% offset.

Loading–unloading–reloading (LUR) testing. Both the samples and parameters used for the LUR tests were the same as those of the monotonic tensile test. Upon straining to a designated strain at the strain rate of ~1 × 10^{-3} s^{-1}, the specimen was unloaded in load mode to 20 N at the unloading rate of 200 N min^{-1}, followed by reloading at a strain rate of ~1 × 10^{-3} s^{-1} to the same applied stress before the next unloading. Following Refs[20,27], we acquired the unloading yield stress (σ_u) and reloading yield stress (σ_r), and divided the flow stress into the back stress (σ_B) and the effective stress (σ_E) using Eq. (1) and (2).

$$\sigma_B = 0.5 \times (\sigma_r + \sigma_u) \quad (2)$$

$$\sigma_E = \sigma_p - \sigma_{B,} \quad (3)$$

where σ_p is the stress at the beginning of unloading.

Electrochemical corrosion testing. A CHI660E electrochemical workstation was used to conduct electrochemical corrosion testing in 3.5 wt.% NaCl solution. In the experimental setup, saturated calomel electrode (SCE) serves as the reference electrode, platinum foil electrode as the counter electrode, and the tested sample acts as the working electrode. The surface area of samples is standardized to 2 mm × 5 mm. To obtain a polished surface, all samples were sequentially abraded using SiC paper ranging from 400 to 2000 grit, followed by polishing with diamond paste. Subsequently, these polished samples were subjected to a final cleaning process using alcohol and deionized water in an ultrasonic bath. Prior to the electrochemical testing, a cathodic polarization at -1000 mV_{SCE} was applied for 200 s. Stable open-circuit potential (OCP) can be generally obtained by immersing the samples in the NaCl solution for a duration exceeding 3600 s. Thereafter, a potentiodynamic polarization curve was accessed under a scanning rate of 1 mV/s, with a potential range extending from -1000 mV_{SCE} to 400 mV_{SCE}.

Fluidity evaluation. The fluidity of cast CCAs can be gauged using a spiral test, which is shown in Fig. 21a. The key part milled into the apparatus is a spiral contour, with a total length over 1500 mm. The fluidity is evaluated by examining the flow length of an alloy in the spiral contour. The evaluation results comparing our cast CCAs with 304 stainless steels are presented in Fig. S21b and c.

Note S1:

Composition design

It was well documented that a precipitation-hardened CCA $(NiCoCr)_{94}(AlTi)_3$ can be obtained by adding Al and Ti elements into the equiatomic NiCoCr system[46-49]. We used this CCA as our starting point. However, the nanoprecipitates produced, in the form of a chemically ordered $Ni_3(Ti,Al)$-type $L1_2$ phase, have relatively low volume fraction (11~16%)[46-49], limiting the enhancement of tensile properties. In addition, their formation requires complex thermomechanical processes, especially lengthy aging (even up to 360 h) due to sluggish diffusion of the elements[49].

We then tuned the alloying elements and their fractions to seek for higher $L1_2$-precipitatate contents. Simultaneously adding more Ni, Al, Ti and/or Ta elements into the equiatomic NiCoCr is a feasible strategy to increase the volume fraction of precipitates, as the Ni, Al, Ti and Ta are strong forming elements of $Ni_3(Ti/Ta,Al)$-type $L1_2$ precipitates[50-52]. Specifically, the Al, Ti and Ta elements are strong $L1_2$-forming elements, while Ni is the main constituent element in $L1_2$. However, excessive additions (>=5 at.% each) of them will catalyze (large-sized) brittle topologically closed packed phases, especially Cr-rich σ at grain boundaries, severely deteriorating ductility[53]. Our recipe increased the Ni content, retained high Co content and reduced Cr, while pushing up an Al-Ta co-addition (~5 at.% Al and 3~4 at.% Ta), in the base equiatomic NiCoCr. The rationale can be summarized as follows, based on prior knowledge[50-59]. Increasing the Ni content can stabilize a ductile FCC matrix. Retaining high Co content, besides stabilizing FCC matrix, can sustain relatively low stacking fault energy (SFE), relieving the SFE increase caused by doping Al, so as to inject stacking faults (SFs) and SF networks to improve properties. Using Ta to replace Ti also reduces the SFE. In addition, the Ta and Al with larger atomic sizes than other constituent elements can impart pronounced solution hardening in the FCC matrix.

In the next step of our screening towards proficient as-cast nanoprecipitation during fast solidification, we used thermodynamic calculations to evaluate the chemical driving force for the formation of the $L1_2$ phase (i.e., the formation energy predicted in the thermodynamic modeling), which helps to establish supersaturation upon moderate undercooling to a sufficiently high temperature at which precipitation kinetics remain rampant. We carried out phase diagram calculation to provide a prediction of the equilibrium volume fraction of $L1_2$ phase (this prediction would be higher than that can be actually reached during the non-equilibrium casting)

over the temperature range of interest. Three representative calculated phase diagrams are shown in Fig. S2. The calculated phase diagrams indicate that with the increase in Al and Ta contents, the $L1_2$-phase fraction rises, to even exceeding 40 vol.%.

In the final step, we screened the candidate alloys using differential scanning calorimetry (DSC at a cooling rate similar to that in direct casting synthesis) to find CCAs showing additional high-temperature exothermic peak corresponding to $L1_2$ precipitation, as exemplified in Fig. S4a. This additional peak led us to desired CCAs that are capable of profuse *in situ* nanoprecipitation during casting, without the need for (costly and/or time-consuming) post-casting treatment.

Overall, taking advantage of prior knowledge to help select alloying elements and combining thermodynamic calculations and calorimetry experiments, one can land CCA compositions conducive to copious precipitation during direct casting synthesis.

Note S2:

Conventional precipitation mechanisms

We revisit here the commonly observed aging or isothermal precipitation cases to help understand the present atypically direct and quick precipitation scenario. For conventional age hardening, the alloy is first heated to a relatively high temperature, so that the solute atoms contained therein can fully dissolve in the matrix lattice. If cooled rapidly (usually quenching), a supersaturated solution is obtained. Re-heating to, and holding at, an intermediate temperature leads to ageing that precipitates out the intermetallic phase.

In our cast case, the time experienced by the alloy at elevated temperatures is short, such that precipitation is limited. Although a high degree of undercooling/supersaturation would be reached when cooled to lower temperatures, atomic diffusion becomes too sluggish then to mediate the formation of intermediate phases. Hence, casting often leaves a supersaturated solution, with some chemical short-range orders in the lattice.

Our goal is therefore to establish adequate supersaturation upon moderate undercooling to a sufficiently high temperature at which precipitation kinetics remain rampant. This is needed to achieve proficient as-cast nanoprecipitation under direct solidification synthesis. As a pointer for the direction to go, the phase diagram calculations in our screening predicts the equilibrium volume fraction of $L1_2$ phase in the temperature range of interest.

Note S3:

Solidifying dendritically with obvious compositional variations

In metallurgy, dendrites are arborescent crystalline structures that grow by diffusion-limited heat and mass transfer. During solidification, at the liquid/solid interface, the solid phase grows by incorporating atoms from the liquid phase. The ensuing formation of dendrites can be influenced by multiple factors, including nucleation, crystallographic anisotropy, diffusion-limited growth, thermal gradient and solidification rate. Among them, solidification rate, the rate at which the material undergoes solidification, plays a crucial role in the dendrite formation. Rapid solidification rates or high undercooling conditions favor the formation of dendrites, especially the appearance of equiaxed dendrites. This is because at high cooling rates, the heat is extracted rapidly, preventing the dendrites from growing in a preferred direction. Instead, equiaxed dendrites with no preferred orientation form. Consequently, the formation of rapidly grown dendrites is the result of a large deviation of the chemical equilibrium state at the solidification front, and is concomitant with extensive compositional differences between the DR and IDR.

Note S4:

Finer in situ precipitates in the leftover FCC matrix

As per discussion above, our CCAs feature the critical ability of supersaturation buildup upon moderate undercooling, with a high thermodynamic driving force to set off the (DSC-detectable) early nucleation burst (Fig. S4a). This happens at rather high temperatures such that atomic diffusion is fast, thereby facilitating the $L1_2$ growth. With further cooling, atomic diffusion slows down, limiting the growth of the already formed precipitates. In the meantime, in the remaining FCC-matrix additional nucleation of $L1_2$ occurs at new locations but with much slower growth rates (Fig. 1j, k and Fig. S8), concomitant with varied chemical compositions in the newly formed precipitates (Fig. 1l and Fig. S7). These sequential events are obviously different from conventional precipitation incidents/mechanisms (Note S2).

In addition, because of microsegregation, the majority of $L1_2$-forming elements, such as Ta, were enriched in IDRs (Fig. 1e). As a result, the average size and population of $L1_2$ precipitates in IDRs are larger than those in the DRs (Fig. 1i). The observation of at least two $L1_2$-nucleation bursts in the IDRs (Fig. 1j-l) is also evidence of the strong driving force for $L1_2$ precipitation in our alloys. Simultaneously, more Ta-rich $D0_{19}$ particles form in the IDRs (Fig. 1b-e). Different element-partitioning trends were detected among multimodal precipitates in DRs and IDRs (Fig. S7), resulting in pronounced nanometer-scale chemical heterogeneities.

Note S5:

Different as-cast microstructures

The microstructure of our high-performance as-cast alloys, designed on the basis of classical precipitation-strengthening concept, is different from that in the paper in *Mater Today* (*2024, 81:70*), which takes a cue from bio-inspired hierarchical structures. As a result, the two sets of alloys achieve properties based on different materials science ideas. Their microstructures are significantly different. At the micro-scale, our designed alloys are dominated by single-phase FCC grains, rather than the FCC+BCC dual-phase-dominated grains in *Mater Today, 2024, 81:70*. At a finer scale, our FCC grains are composed of highly branched dendrites, while the BCC grains in the Mater Today paper consist of sub-micrometer $L2_1$ particles with shell structures (i.e., exhibiting a core-shell configuration). At the nanometer scale, our dendrites contain *in situ* single-phase ($L1_2$) nanoprecipitates with a gradient of sizes increasing from the branched DR to IDR, while the leftover space of their BCC grains and FCC grains comprise multimodal and multi-phase nanoprecipitates (e.g., B2, η-Ni_3Ti and $L1_2$).

Note S6:

Pursuing high ductility for high-strength cast alloys

Figure 2c shows that the combination of yield strength and uniform elongation in our alloys, especially the latter (~32%), is obviously superior to that in *Mater Today, 2024, 81:70*. In addition, compared to other reported as-cast properties, our samples exhibit significantly higher yield strength at equivalent elongation or much higher elongation at equivalent yield strength. These superior property trends also lead to a considerably higher product of yield strength and uniform elongation (Fig. 2d), which further confirms the exceptional as-cast mechanical properties achieved by our designed alloys.

It is important to pursue high ductility for high-strength cast alloys. Specifically, ductility indicates a material's ability to deform plastically before fracturing, in which high uniform elongation allows the plastic flow to proceed in a controlled manner without run-away failure. Thus, obtaining higher ductility and uniform elongation (e.g., >10%) is essential for materials that need to absorb energy and/or undergo significant deformation without breaking, which can lead to more efficient and safer designs.

Even though cast alloys are used in the as-cast form without subsequent deformation processing, a high ductility is in fact necessary for safety and reliability in engineering applications. We emphasize here that 10% tensile ductility is in fact inadequate for as-cast parts in many applications. For instance, ASME BPVC sets lower bounds for %elongation for cast products. To name a few, the lower limit required for (low-temperature) ductile irons (such as QT400-18L, QT400-18AL and EN-GJS-400-18) is ≥18%[60-64], and that for the cast austenitic stainless steel (ASTM-A351-CF8M) is ≥30%[63,64]. Specifically, a wide range of demanding applications require a large safety margin, and/or a large energy absorbtion, from yield to failure. Examples for >30% ductility are primarily for safety-critical applications of cast alloys in aerospace and medical fields, and under extreme (repeated impact, corrosive, high-temperature, and high-pressure) conditions[60-64]. Under these circumstances, custom-made specific parts are often desired, including crankshafts, camshafts, connecting rods, gears, hydraulic cylinder blocks, wind power castings (such as impeller hubs and gearboxes), automobile wheels, chassis components and chassis components, etc.

To reiterate, high-performance cast alloys do demand ductility well above 10%, in addition to GPa-level as-cast yield strength. Our as-cast tensile ductility as high as ~32%, including uniform elongation far more than 10%, stands out in this regard (Fig. 2c). The highlighted reasons above explain why, while seeking for GPa-level as-cast yield strength, we also pursue at the same time higher as-cast tensile ductility (~32%), especially uniform elongation with far more than 10%.

Note S7:

ASM and ASTM Handbooks

Nunes, R. Adams, J. H. & Ammons, M., ASM handbook volume 2: Properties and selection: nonferrous alloys and special-purpose materials. ASM International, US, (1990).

ASTM B367-22, Standard specification for titanium and titanium alloy castings (2022).

ASTM B381-21, A., Standard specification for titanium and titanium alloy forgings. (2021).

ASTM B265-20a, A., Standard specification for titanium and titanium alloy strip, sheet, and plate. (2020).

ASTM B564-22, A., Standard specification for nickel alloy forgings. (2022).

A494/A494M-24, A., Standard specification for castings, nickel and nickel alloy. (2024).

Aluminum Alloys

H1X, Strain-Hardened Only. The digit **X** following the H1 indicates the degree of strain hardening.

H2X, Strain-Hardened and Partially Annealed. The digit **X** following the H2 indicates the degree of strain hardening remaining after the product has been partially annealed.

H3X, Strain-Hardened and Stabilized. The digit **X** following the H3 indicates the degree of strain hardening remaining after stabilization.

Hx11, applies to products that incur sufficient strain hardening after final annealing to fail to qualify as 0 temper, but not so much or so consistent an amount of strain hardening to qualify as Hx1 temper.

H112, pertains to products that may acquire some strain hardening during working at elevated temperature and for which there are mechanical property limits.

T1, Cooled From an Elevated-Temperature Shaping Process and Naturally Aged to Substantially Stable Condition.

T3, Solution Heat Treated, Cold Worked, and Naturally Aged to a Substantially Stable Condition.

T4, Solution Heat Treated and Naturally Aged to a Substantially Stable Condition.

T5, Cooled From an Elevated-Temperature Shaping Process and Artificially Aged.

T6, Solution Heat Treated and Artificially Aged.

T7, Solution Heat Treated and Overaged or Stabilized.

T8, Solution Heat Treated, Cold Worked, and Artificially Aged.

T4X, T6X, T7X and T8X, Additional T Temper Variations. When it is desirable to identify a variation of T tempers described above, additional digits, the first of which cannot be zero, may be added to the designation, like T42, T451, T62……

Magnesium Alloys

H1X, Slightly strain hardened.

H2X, Strain hardened and partially annealed.

T-X, Heat treated, including solution heat treated, aged and soon.

Copper Alloys

H-X: Cold-worked tempers

O & OS: Annealed tempers

M-X: As-manufactured tempers

TM-X: Mill-hardened tempers

TD-X: Solution-treated and cold-worked tempers

TH-X: Cold-worked and precipitation-hardened tempers

TF00: Solution-treated and precipitation-hardened temper

Titanium Alloys

Titanium and Titanium Alloy Forgings

α: Grade 1, 2, 2H, 3, 7, 7H[B C], 11, 12, 13, 14, 15, 16, 16H[B C], 17, 26, 26H[B C], 27, 30, 31, 33, 34, 37.

β: Grade 4, 9[D], 18, 18[D], 28, 32, 36

γ: Grade 19[E], 20[E], 21[E]

δ: Grade 5, 6, 9, 19[F], 20[F], 21[F], 23, 24, 25, 29, 35, 38

ε: Grade 19[G], 20[G], 21[G]

Titanium and Titanium Alloy Strip, Sheet, and Plate

ζ: Grade 1, 2H[C D], 7, 7H[C D], 11, 13, 14, 16, 16H[C D], 17, 26 H[C D], 27, 30, 33, 37

η: Grade 3, 4, 9, 12, 15, 18, 28, 31, 32, 34, 36, 39, 40

θ: Grade 5, 6, 19[G H], 20[G H], 21[G H], 23, 24, 25, 29, 35, 38

Titanium and Titanium Alloy Castings

ι: Grade 5, 6, 38

κ: Grade 2, 3, 7, 8, 9, 12, 16, 17, 18

Grade 1—Unalloyed titanium

Grade 2—Unalloyed titanium

Grade 2H—Unalloyed titanium (Grade 2 with 400 MPa minimum UTS)

Grade 3—Unalloyed titanium

Grade 4—Unalloyed titanium

Grade 5—Titanium alloy (6 % aluminum, 4 % vanadium)

Grade 6—Titanium alloy (5 % aluminum, 2.5 % tin)

Grade 7—Unalloyed titanium plus 0.12 to 0.25 % palladium

Grade 7H—Unalloyed titanium plus 0.12 to 0.25 % palladium (Grade 7 with 400 MPa minimum UTS)

Grade 9—Titanium alloy (3.0 % aluminum, 2.5 % vanadium)

Grade 11—Unalloyed titanium plus 0.12 to 0.25 % palladium

Grade 12—Titanium alloy (0.3 % molybdenum, 0.8 % nickel)

Grade 13—Titanium alloy (0.5 % nickel, 0.05 % ruthenium)

Grade 14—Titanium alloy (0.5 % nickel, 0.05 % ruthenium)

Grade 15—Titanium alloy (0.5 % nickel, 0.05 % ruthenium)

Grade 16—Unalloyed titanium plus 0.04 to 0.08 % palladium

Grade 16H—Unalloyed titanium plus 0.04 to 0.08 % palladium (Grade 16 with 400 MPa minimum UTS)

Grade 17—Unalloyed titanium plus 0.04 to 0.08 % palladium

Grade 18—Titanium alloy (3 % aluminum, 2.5 % vanadium) plus 0.04 to 0.08 % palladium

Grade 19—Titanium alloy (3 % aluminum, 8 % vanadium, 6 % chromium, 4 % zirconium, 4 % molybdenum)

Grade 20—Titanium alloy (3 % aluminum, 8 % vanadium, 6 % chromium, 4 % zirconium, 4 % molybdenum) plus 0.04 % to 0.08 % palladium

Grade 21—Titanium alloy (15 % molybdenum, 3 % aluminum, 2.7 % niobium, 0.25 % silicon)

Grade 23—Titanium alloy (6 % aluminum, 4 % vanadium with extra low interstitial elements, ELI)

Grade 24—Titanium alloy (6 % aluminum, 4 % vanadium) plus 0.04 % to 0.08 % palladium

Grade 25—Titanium alloy (6 % aluminum, 4 % vanadium) plus 0.3 % to 0.8 % nickel and 0.04 % to 0.08 % palladium

Grade 26—Unalloyed titanium plus 0.08 to 0.14 % ruthenium

Grade 26H—Unalloyed titanium plus 0.08 to 0.14 % ruthenium (Grade 26 with 400 MPa minimum UTS)

Grade 27—Unalloyed titanium plus 0.08 to 0.14 % ruthenium

Grade 28—Titanium alloy (3 % aluminum, 2.5 % vanadium) plus 0.08 to 0.14 % ruthenium

Grade 29—Titanium alloy (6 % aluminum, 4 % vanadium with extra low interstitial elements, ELI) plus 0.08 to 0.14 % ruthenium

Grade 30—Titanium alloy (0.3 % cobalt, 0.05 % palladium)

Grade 31—Titanium alloy (0.3 % cobalt, 0.05 % palladium)

Grade 32—Titanium alloy (5 % aluminum, 1 % tin, 1 % zirconium, 1 % vanadium, 0.8 % molybdenum)

Grade 33—Titanium alloy (0.4 % nickel, 0.015 % palladium, 0.02 5 % ruthenium, 0.15 % chromium)

Grade 34—Titanium alloy (0.4 % nickel, 0.015 % palladium, 0.025 % ruthenium, 0.15 % chromium)

Grade 35—Titanium alloy (4.5 % aluminum, 2 % molybdenum, 1.6 % vanadium, 0.5 % iron, 0.3 % silicon)

Grade 36—Titanium alloy (45 % niobium)

Grade 37—Titanium alloy (1.5 % aluminum)

Grade 38—Titanium alloy (4 % aluminum, 2.5 % vanadium, 1.5 % iron)

Grade 39—Titanium alloy (0.25 % iron, 0.4 % silicon)

Grade 40—Titanium alloy (3.9 % vanadium, 0.85 % aluminum, 0.25 % iron, 0.25 % silicon).

Steels

SS: Stainless steels

QT steels: Quenched and tempered steels

BH steels: Bake hardenable steels

CP steels: Complex phase steels

DP steels: Dual phase steels

IF steels: Interstitial free steels

IS steels: Isotropic steels

TRIP steels: Transformation induced plasticity steels

Note S8:

Strength contribution calculation

The yield strength σ_y of metallic materials is usually considered as the sum of different strengthening mechanisms independently[35,58,59]. Here, the σ_y of A5T3 can be given as:

$$\sigma_y = \sigma_0 + \sigma_{ss} + \sigma_{dis} + \sigma_{GB} + \sigma_{L12} + \sigma_{D019} \tag{4}$$

where σ_0, σ_{ss}, σ_{dis}, σ_{GB}, σ_{L12}, and σ_{D019} represent the lattice friction stress, solid-solution hardening, dislocation hardening, grain-boundary hardening, $L1_2$ precipitation hardening, and micron-scale $D0_{19}$ particle hardening, respectively. The σ_{dis} part can be ignored in the analysis as all the undeformed samples were in the as-cast state.

The contribution of σ_{ss} induced by solute atoms within the matrix can be incorporated into the lattice resistance σ_0 for CCAs[65,66]. Thus, the value of (σ_0 + σ_{ss}) in our work can be estimated by employing the newly-developed Varvenne's solid-solution hardening model[66,67]. This model was developed specifically for the random multi-principal alloys and has shown quantitative success for many CCAs[66,67]. The model suggests that the solute strengthening in a concentrated solid solution can be described by an effective average matrix ("solvent") with all atoms being embedded "solute" atoms[66,67]. An important concept in this model is the misfit volume $\triangle V_n$, which is proposed to describe the local lattice distortion for solute n in a given alloy[66]. $\triangle V_n$ of each type of specific solute-n (reflecting the average local atomic environment around atom-n) can be expressed as[66,67]:

$$\Delta \mathrm{V_n} = \sum_\mathrm{m} \mathrm{C_m} \left[\frac{\partial \overline{V}}{\partial c_\mathrm{n}} - \frac{\partial \overline{V}}{\partial c_\mathrm{m}}\right] \tag{5}$$

where c_m and c_n represent the content of the solute-m and -n species, respectively. As an estimation, $\triangle V_n$ can also be calculated as the difference between the apparent atomic volume V_n and the average atomic volume $\overline{V}$, namely, $\triangle V_n = V_n - \overline{V}$. $\overline{V}$, for simplicity, can be determined using Vegard's law, $\overline{V} = \sum_\mathrm{n} \mathrm{c_n} V_\mathrm{n}$[66,67].

Therefore, the predicted zero-temperature, shear yield stress τ_{y0}, and energy barrier $\triangle E_b$ for dislocation motion thermally activated of a material can be given as[66,67]:

$$\tau_{y0} = 0.01785 \alpha^{-\frac{1}{3}} K_\tau \left[\sum_n c_n \Delta V_n^2\right]^{\frac{2}{3}} \tag{6}$$

$$\Delta E_b = 1.5618 \alpha^{\frac{1}{3}} K_{\Delta E} \left[\sum_n c_n \Delta V_n^2\right]^{\frac{1}{3}} \tag{7}$$

$$K_\tau = G \left(\frac{1+v}{1-v}\right)^{\frac{4}{3}} b^{-4} \text{ and } K_{\Delta E} = G \left(\frac{1+v}{1-v}\right)^{\frac{2}{3}} \tag{8}$$

where v is the Poisson ratio and G is the shear modulus. As the chemical composition of the matrix in this material is very close to that of the NiCoCr alloy, the values of v and G are adopted as what was reported in NiCoCr alloy, which are 0.3 and 87 GPa, respectively. Additionally, the parameter α is set to be 0.123

associated with the line tension of an edge dislocation in FCC-structured alloys. The values of V_n for each constituent element are adopted from previous publications[66,67]. The strengthening effect of Zr and B is not considered here due to their minor content.

Table S2: *The values of V_n for Ni, Co, Cr, Al, Ta*

Element	V_n (Å^3)
Ni	10.94
Co	11.12
Cr	12.27
Al	14.00
Ta	17.35

At a given temperature T and strain rate $\dot{\varepsilon}$, the thermal activation theory leads to the predicted yield shear stress $\tau_y(T,\ \dot{\varepsilon})$ as:

$$\tau_y(T, \dot{\varepsilon}) = \tau_{y0}\left[1 - \left(\frac{k_B T}{\Delta E_b}\ln\frac{\dot{\varepsilon}_0}{\dot{\varepsilon}}\right)^{\frac{2}{3}}\right] \tag{9}$$

where $\dot{\varepsilon} = 10^{-3}\ s^{-1}$ is the experimental strain rate, $\dot{\varepsilon} = 10^{4}\ s^{-1}$ is a reference strain rate, and $k_B = 1.38 \times 10^{-23}$ J/K is the Boltzmann constant[67]. Accordingly, the theoretical tensile yield stress $\sigma_{y(th)}$ can be given by:

$$\sigma_{y(th)} = M\tau_y \tag{10}$$

where M = 3.06 is the Taylor factor. Based on Eqs. (5) - (10), it can be calculated that $\sigma_0 + \sigma_{ss}$ = 208.6 MPa at 300 K.

The σ_{GB} can be calculated from the Hall–Petch relationship:

$$\sigma_{GB} = k_{GB} D^{-\frac{1}{2}} \tag{11}$$

where k_{GB} = 556 MPa $\mu m^{1/2}$ was extracted from NiCoCr alloy[66]. Thus, σ_{GB} can be calculated to be 75.5 MPa considering the average grain size D = 54.2 μm.

The micro-sized particles in an alloy normally show limited influences on the dislocation motion. Their contribution to strength is caused by the load-bearing effect. A simple but generally accepted formula for the load-bearing strength increment is[68]:

$$\sigma_{DO19} = 0.5 V_{DO19}\, \sigma_M \tag{12}$$

where V_{D019} (~5.2 vol%) is the volume fraction of $D0_{19}$ particles, and σ_M is the matrix strength of CCAs, which is equal to the sum of the first five terms in Eq. (4). Therefore, Eq. (4) can be rewritten as:

$$\sigma_y = (1 + 0.5V_{D019}) \times (\sigma_0 + \sigma_{ss} + \sigma_{dis} + \sigma_{GB} + \sigma_{L1_2}) \tag{13}$$

It can be calculated that σ_{L1_2} = 534.6 MPa and σ_{D019} = 21.3 MPa.

Note S9:

An example of degraded ductility due to increased stress concentration

When the multiple coupled defect modes discussed in the main text are less activated, plastic flow can be localized, and damage incurred via stress concentrations becomes a threat to ductility. To further demonstrate this, we modified our directly synthesized as-cast specimens by high-temperature homogenization annealing at 1200 °C for 12 h and aging at 800 °C for 4 h. A homogenous nanoprecipitation structure with sporadic undissolved $D0_{19}$ particles is achieved, as shown in Fig. S15 and its inset. The resulting aged samples also exhibit a high yield strength (comparable to as-cast CCA counterparts), yet suffering from an inferior tensile ductility of 16~18% (Fig. S16). We show that the markedly reduced ductility can be ascribed to premature damage emerging from stress concentrations. Microstructure investigations unveil that in aged CCAs, strong (primary) planar slip concomitant with slip-plane softening (Fig. S17a, b) severely localizes plastic deformation near grain boundaries (Fig. S17c). At the nanoscale, it is expected that upon stress loading a large number of intragranular planar-slip dislocations glide towards, as well as finally accumulate densely at, grain boundaries, as indicated by much higher KAM values (Fig. S17d). These cause high stress concentrations at grain boundaries, resulting in intergranular cracking and premature failure, as shown in Fig. S17e, f. In contrast, in directly solidified as-cast CCAs, as discussed extensively in the main text the *in situ* gradient nanoprecipitates regulate a progressive, multi-scale and multi-mode-coupled plastic flow, which effectively spread the flow and avoids severe stress concentrations and excessive dislocations piling-up at grain boundaries, as revealed in Fig. S18. Instead, dislocation accumulation and entanglement inside the grains are promoted (Fig. 4), rather than at grain boundaries. The corresponding fracture surface exhibits extensive dimples (Fig. S19a-c), in contrast to the aged CCAs, which showed a relatively smooth fracture with significantly fewer dimples (Fig. S19d-f) due to large-scale intergranular cracking.

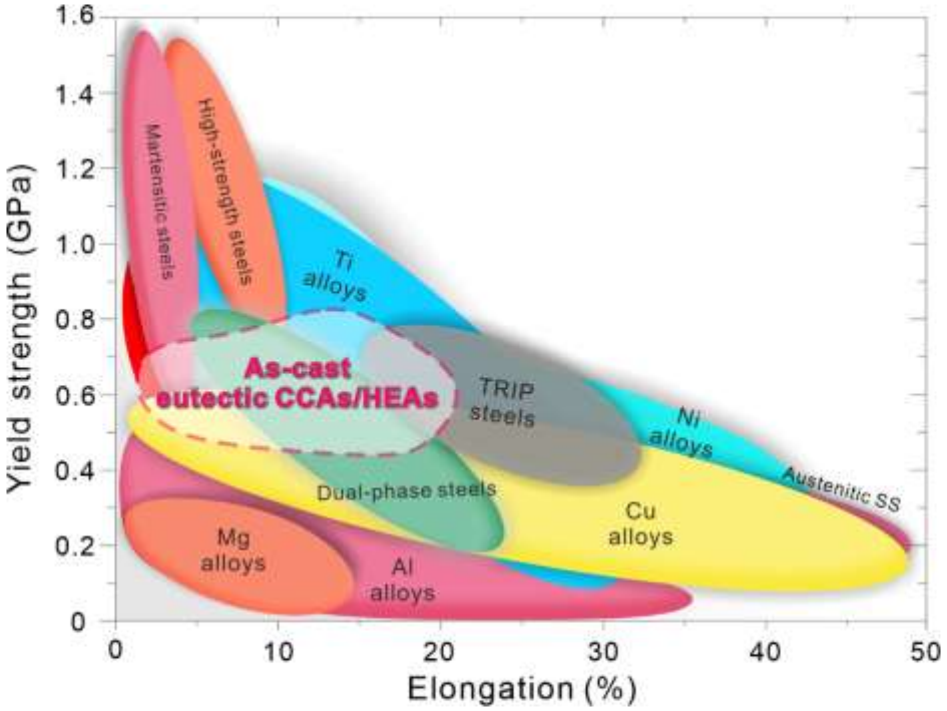

Figure S1 | Performance comparison chart of yield strength *versus* elongation. The as-cast eutectic CCAs (in the dashed circle) have not yet reached the best strength−ductility previously achieved in commercial alloys and steels (various colored regions) processed via well-established post-cast treatments. See detailed data and associated references of these as-cast eutectic CCAs/HEAs and commercial materials in **Table S4** and **Note S7**.

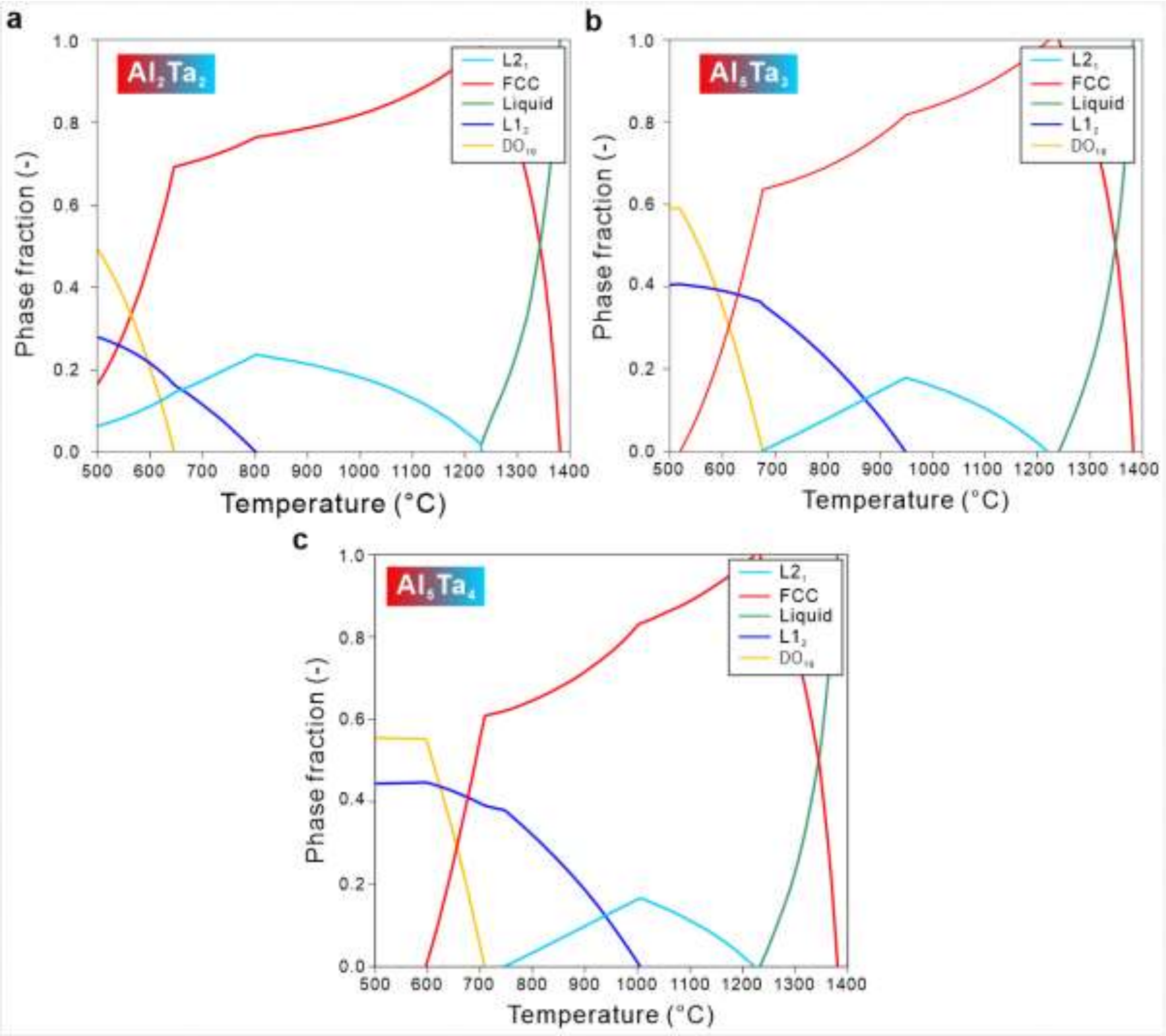

Figure S2 | CALPHAD phase diagrams for our studied Ni-Co-Cr-Al-Ta CCA system. See discussion in **Note S1**.

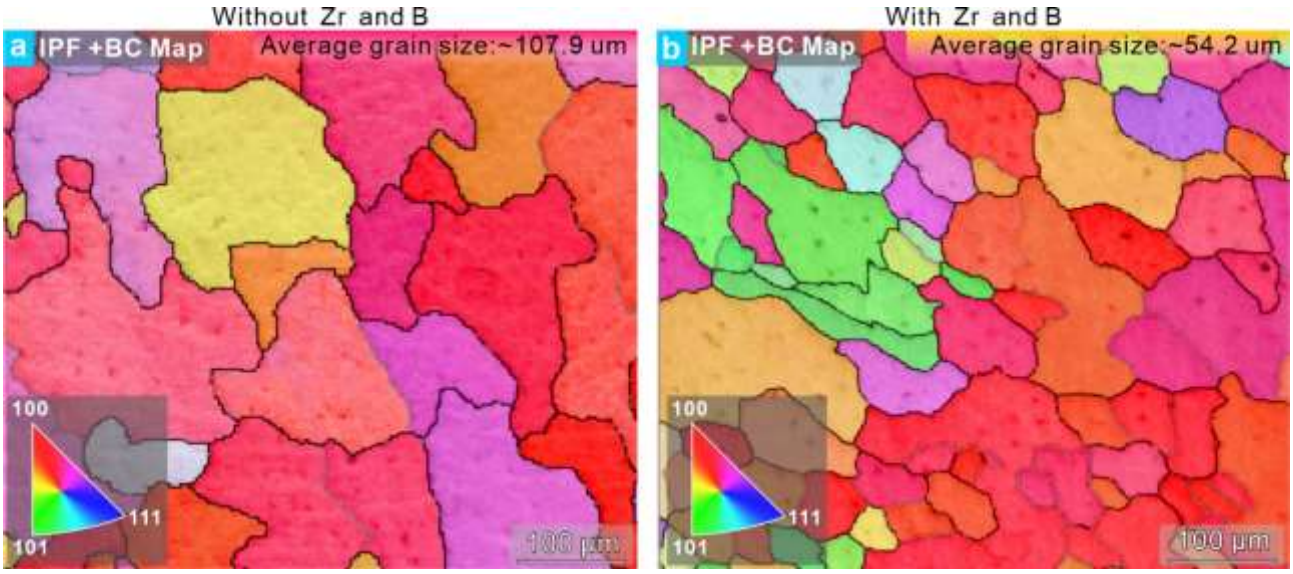

Figure S3 | EBSD IPF image of our directly solidified as-cast CCAs (A5T3), revealing that adding minor Zr and B elements (~0.006 wt.% and ~0.10 wt.%, respectively) can render a significant grain refinement (~54.2 μm), as seen in (**b**), relative to that without, ~107.9 μm in (**a**). Trace amounts of Zr and B render grain refinement through heterogeneous nucleation. The refined grains can impact the formation of nanoscale $L1_2$ precipitates by influencing atom diffusion and providing more nucleation sites. Grain boundaries provided by refined grains can act as diffusion highways, accelerating the redistribution of solute atoms needed for the $L1_2$ precipitation.

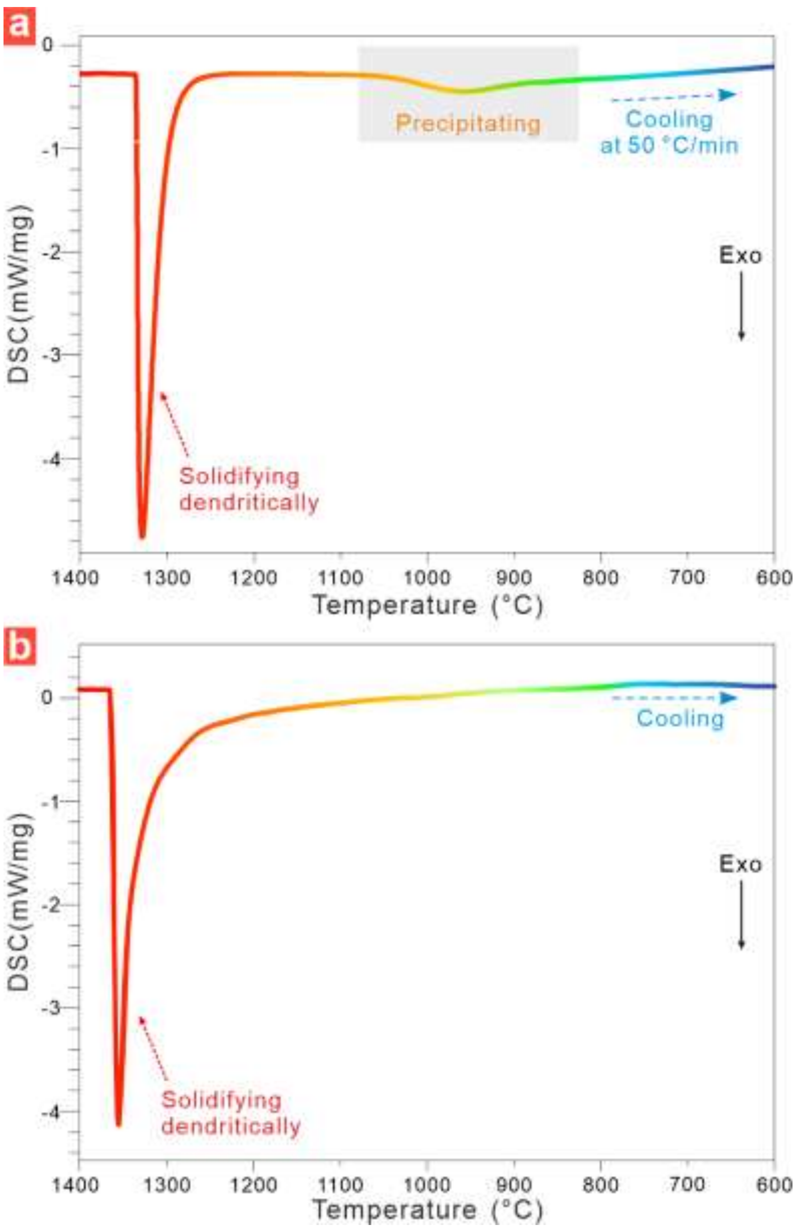

Figure S4 | DSC scan at 50 ℃/min showing a precipitation peak, and mimicking the solidification and phase transformation (*in situ* precipitation) in our CCA (a). The normal $(CoCrNi)_{94}(TiAl)_3$ CCA (**b**) does not show precipitation upon such fast cooling.

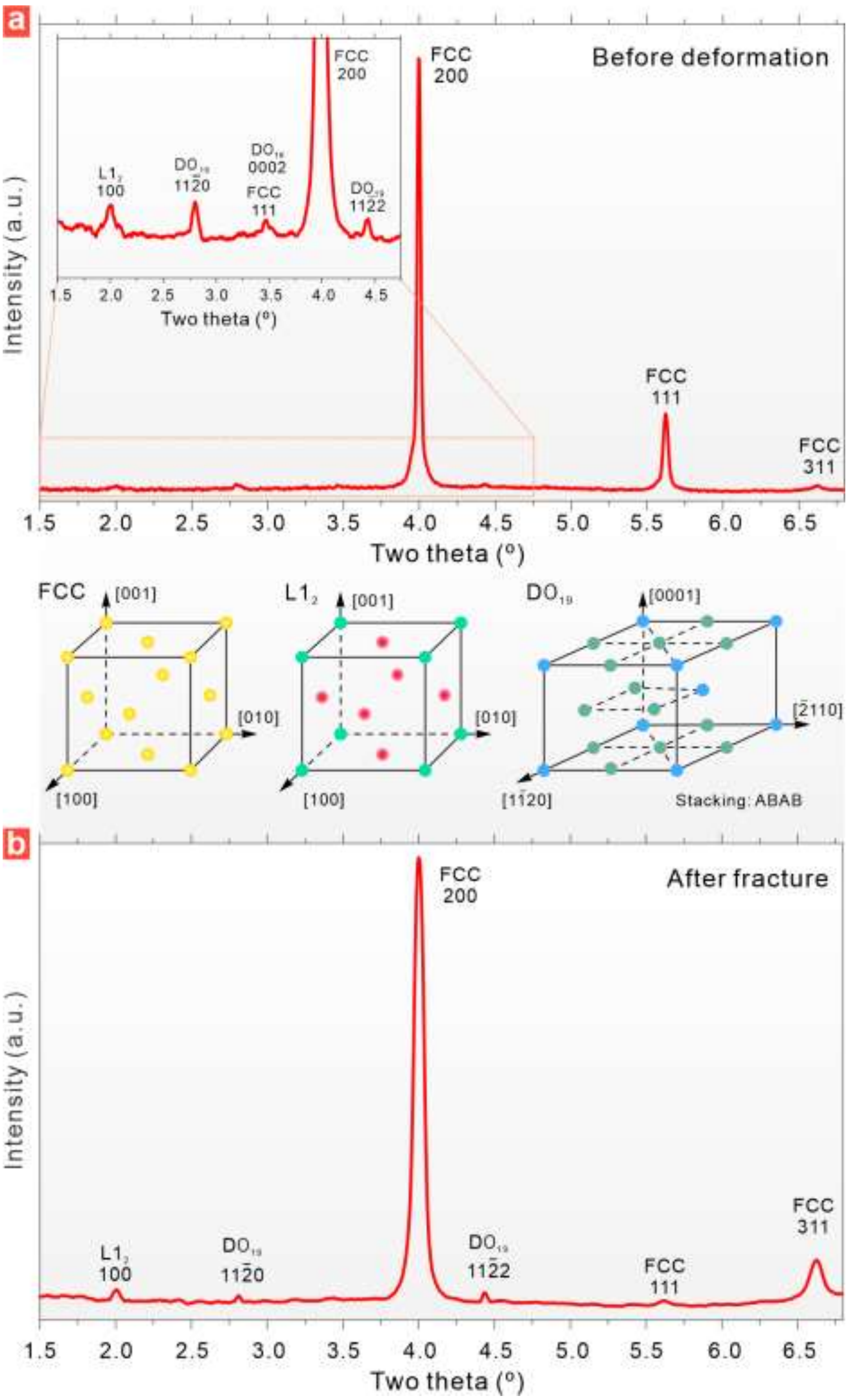

Figure S5 | Synchrotron high-energy X-ray diffraction (SHE-XRD) spectra before deformation (a) and after fracture (b). Three crystal structures of FCC, $L1_2$ and $D0_{19}$ are schematically illustrated in the bottom panel (**a**). No new diffraction peaks (i.e., from TRIP) are detected after fracture in the SHE-XRD curve (**b**).

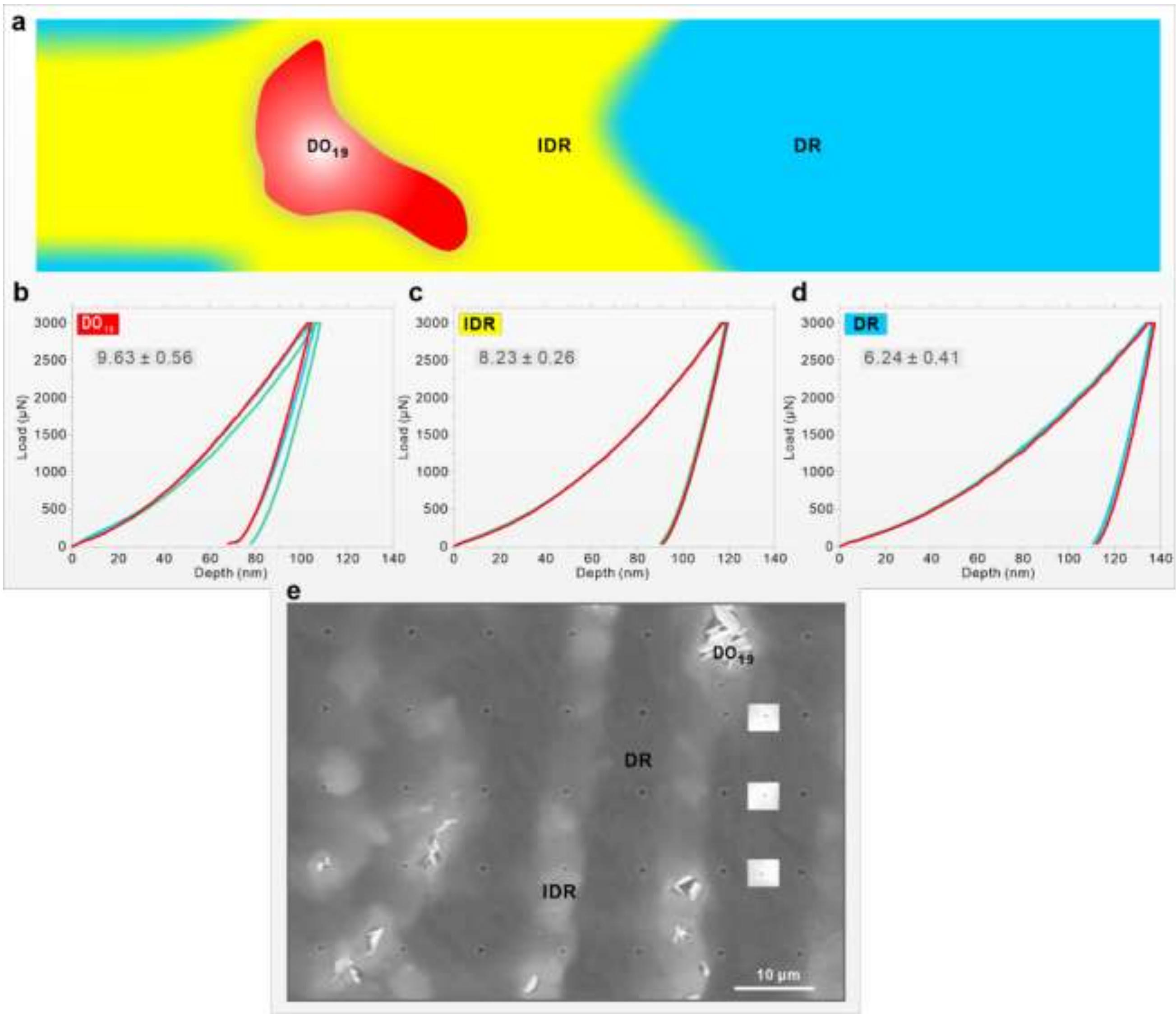

Figure S6 | Nanoindentation tests. (**a**) Schematic diagram showing $D0_{19}$, DR and IDR regions. (**b-d**) Nanoindentation curves of these regions, respectively, with corresponding hardness values. (**e**) SEM image of nanoindentations. Evaluating hardness values of the $D0_{19}$, DR, and IDR by making a 5×7 nanoindentation testing in multiple areas. The three SEM insets (**e**) show additional and clearer nanoindentations of $D0_{19}$. Three insets and the main image have the same magnification (the same scale bar).

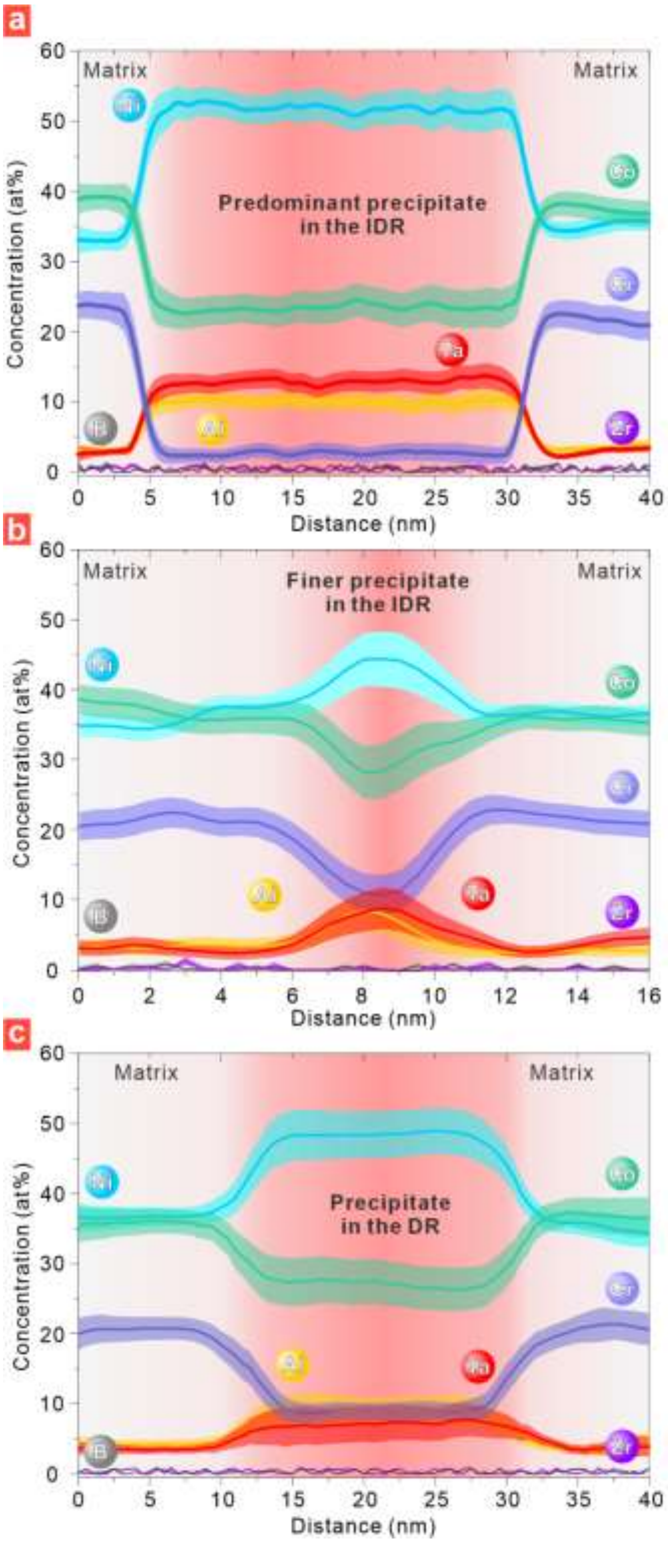

Element	$K^{\gamma/\gamma'}$
Al	3.373
Cr	0.115
Ni	1.516
Co	0.615
Ta	5.498

Element	$K^{\gamma/\gamma'}$
Al	2.016
Cr	0.701
Ni	1.200
Co	0.823
Ta	2.319

Element	$K^{\gamma/\gamma'}$
Al	2.486
Cr	0.467
Ni	1.266
Co	0.792
Ta	3.154

Figure S7 | (a-c) 1D compositional profiles, obtained from 3D APT probing, of $L1_2$ nanoprecipitates across IDRs and DRs, as well as corresponding element partitioning coefficients, $K^{\gamma/\gamma'}$.

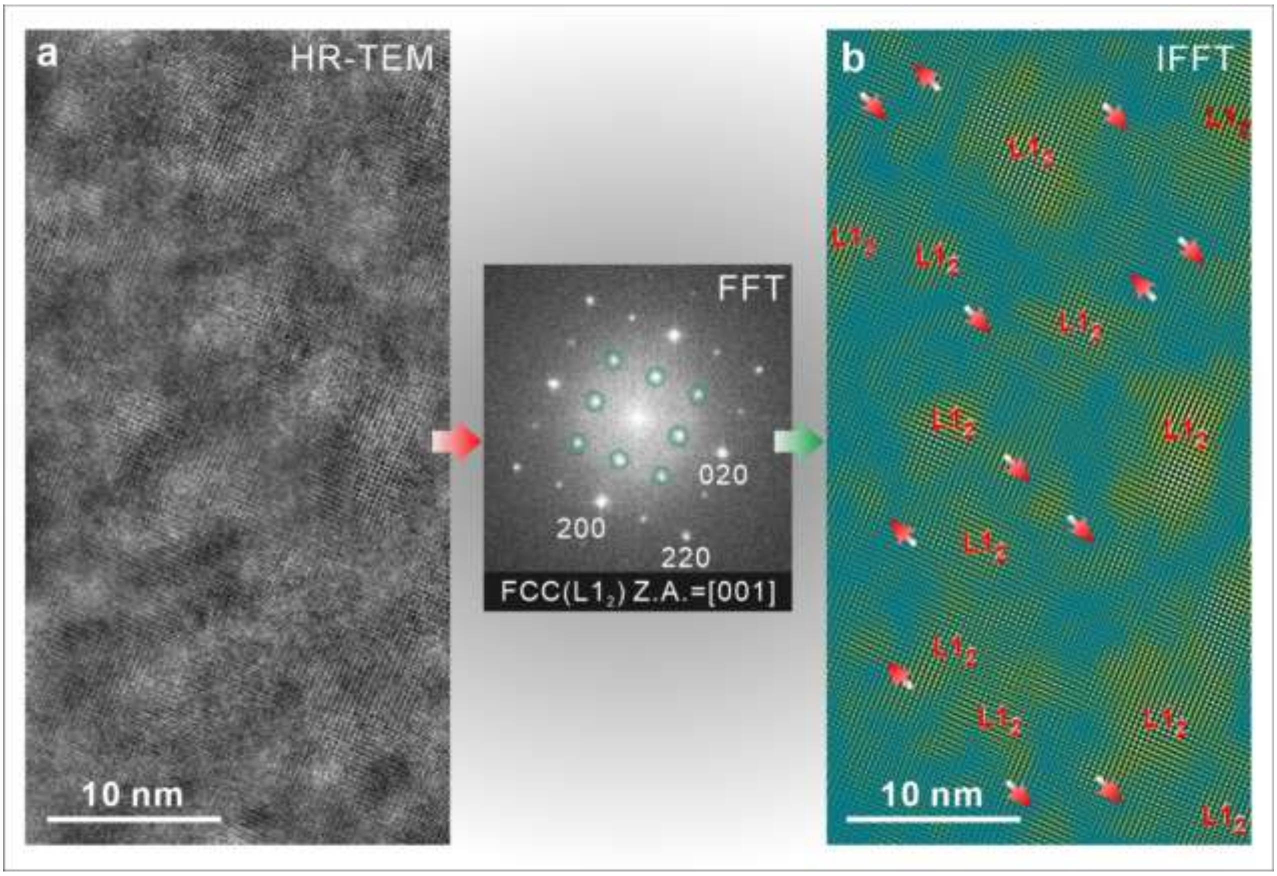

Figure S8 | Fine L1$_2$ nanoprecipitates detected in IDRs. (**a**) High-resolution (HR) TEM image. (**b**) Corresponding FFT confirming the existence of L1$_2$ nanoprecipitates, as evidenced by L1$_2$-type superlattice spots (circled by green lines). (**c**) Corresponding IFFT image of L1$_2$ precipitates, manifesting as yellow regions (labeled as L1$_2$). Some extremely fine L1$_2$ precipitates (<3 nm) are marked using red arrows. The very different L1$_2$ sizes hint multiple nucleation bursts at different temperatures.

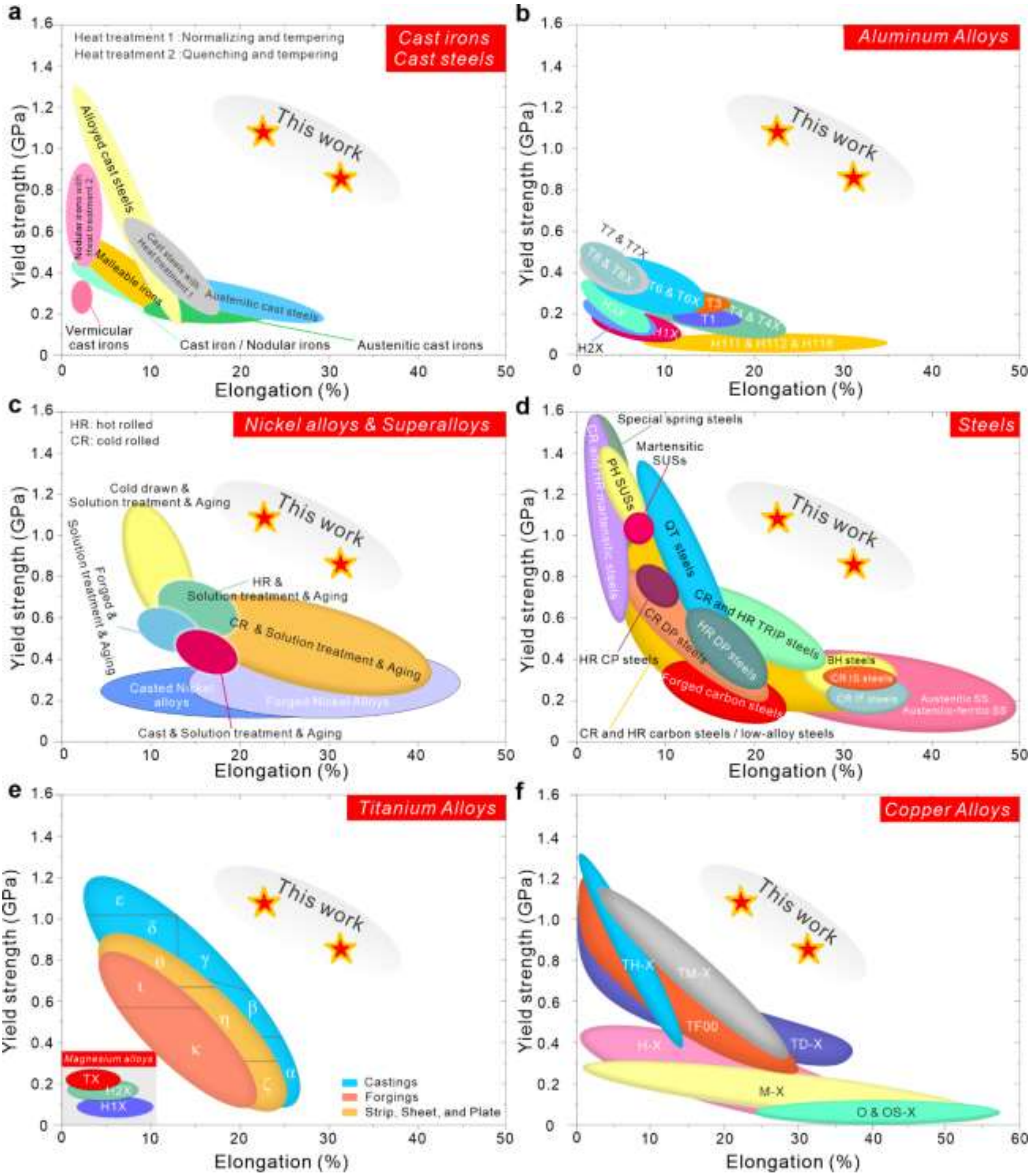

Figure S9 | Performance charts to compare with each separate group summarized in Fig. 2f: cast irons and steels (**a**), Al alloys (**b**), Nickel alloys & Superalloys (**c**), diverse steels (**d**), Ti and Mg alloys (**e**), and Cu alloys (**f**). The materials in (**a-f**) are detailed in **Note S7**.

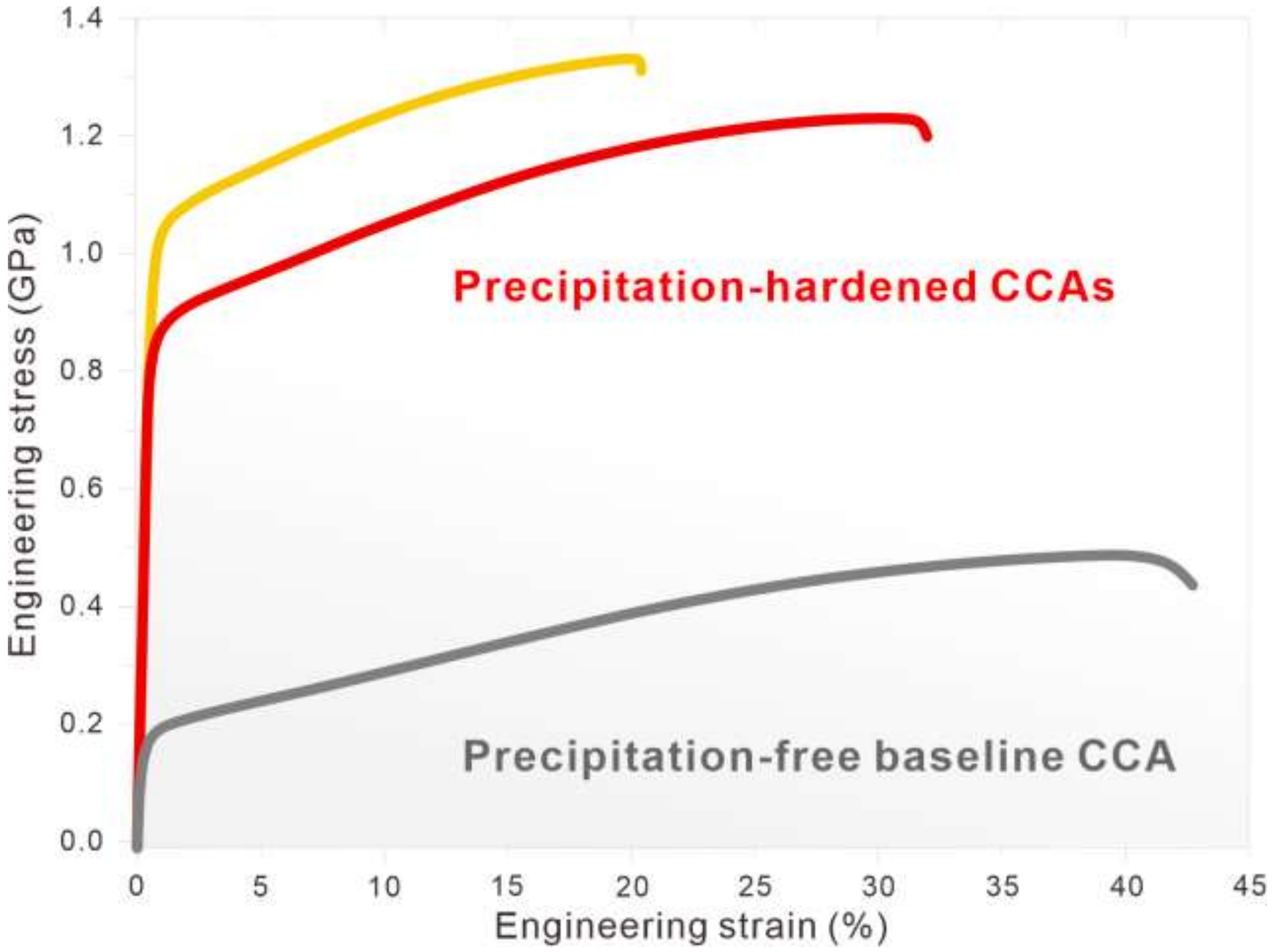

Figure S10 | Engineering stress-strain curves of two as-cast precipitation-hardened CCAs (A5T3 and A5T4), compared with the precipitation-free as-cast CCA baseline.

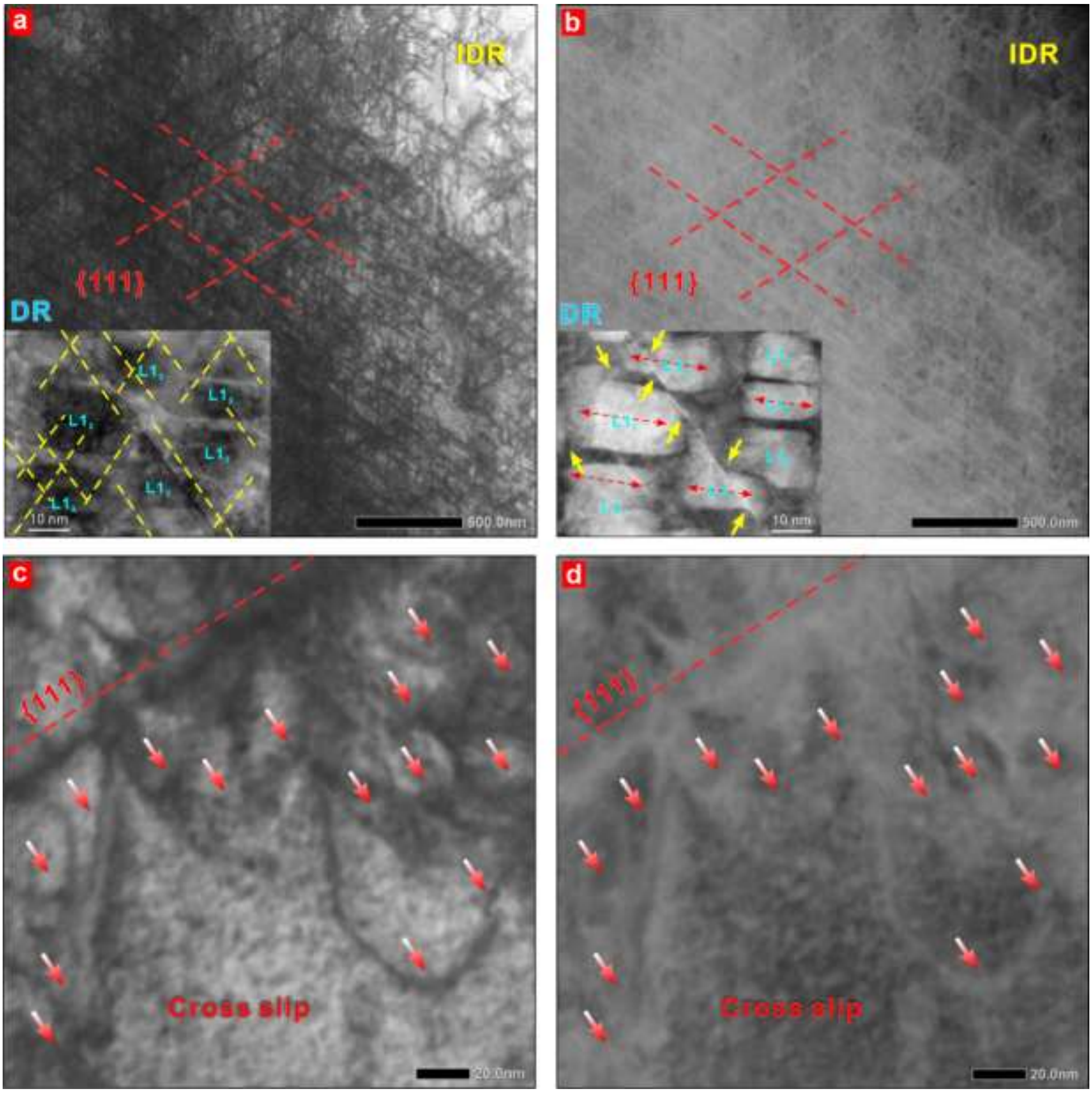

Figure S11 | Wide-spread defect interactions and dislocation cross-slip. (**a, b**) Complementary STEM and LAADF-STEM images, revealing exceedingly profuse intersections of primary and secondary planar {111} dislocations as well as frequent dislocation cross slip, which are especially remarkable in the DR (compared to that in the IDR). The two insets (**a, b**) reveal that the $L1_2$ phase was cut off and misaligned by planar-slip dislocations and stacking faults. Due to dense dislocation-mediated deformation activity, the $L1_2$ phase was markedly elongated (see red arrows; inset, **b**) compared to before deformation. (**c, d**) Enlarged STEM and LAADF-STEM images, revealing wide-spread dislocation cross-slip (marked by red arrows) far away from the {111} slip band (marked by red dotted lines).

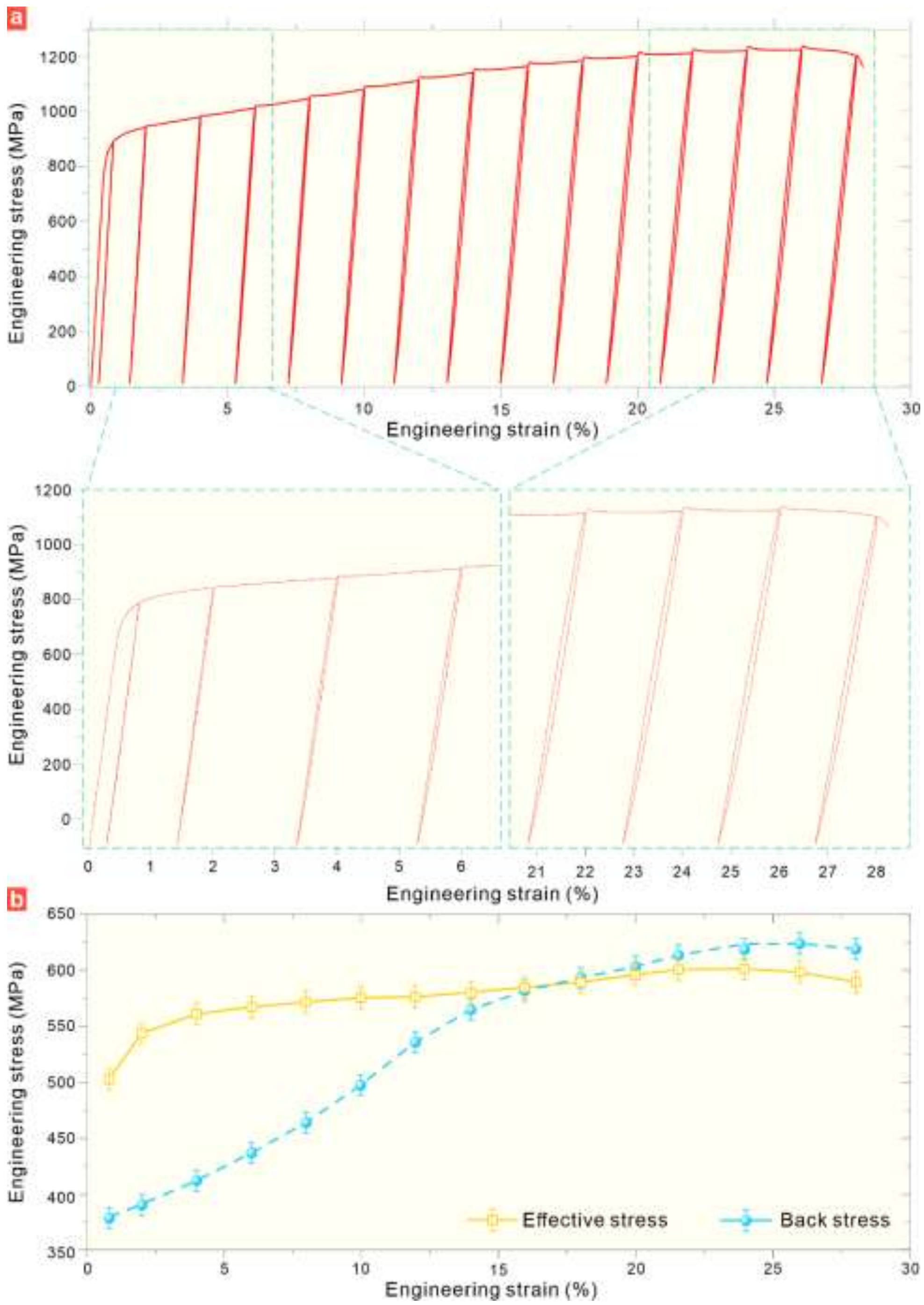

Figure S12 | Loading–unloading–reloading (LUR) curve (a) and the corresponding evolution of back stress and effective stress (b). The insets (**a**) show the partially magnified LUR curve. The back stress starts out relatively small in (**b**), because the effective stress is overwhelming, stemming from the predominant short-range interactions between dislocations and nanoscale $L1_2$ precipitates as well as among multiple modes of dislocations (Fig. 4, Fig. S11, and their insets). Such stress trends are confirmed by the closed LUR loop (overlapping curves) in the early strain stage and the somewhat expanded LUR loop in the later strain stage (see **a** and its inset).

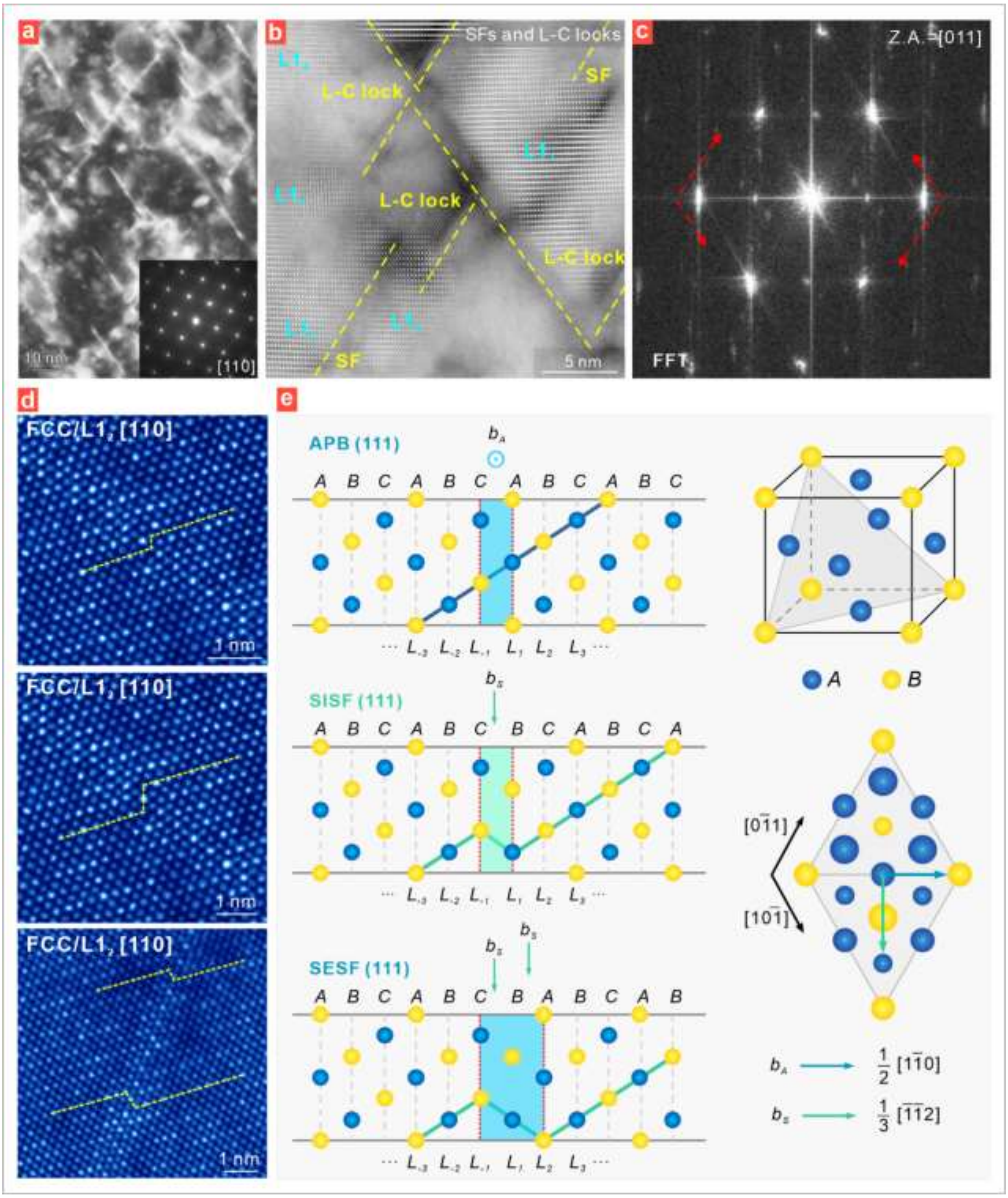

Figure S13 | SFs and L-C locks. (**a**) ADF STEM image and corresponding SAED inset. (**b**) Atomic-scale TEM image. (**c**) Corresponding FFT image. (**d**) Atomic-resolution HAADF-STEM image showing superlattice intrinsic and extrinsic stacking faults (SISF and SESF, respectively). (**e**) Schematic diagrams of corresponding formation mechanisms/processes of SISF and SESF. No twinning deformation is detected in (**a-d**). These images (**a-d**) collectively reveal multiple interactions among the L1$_2$ phase, SISF, SESF and L-C

locks.

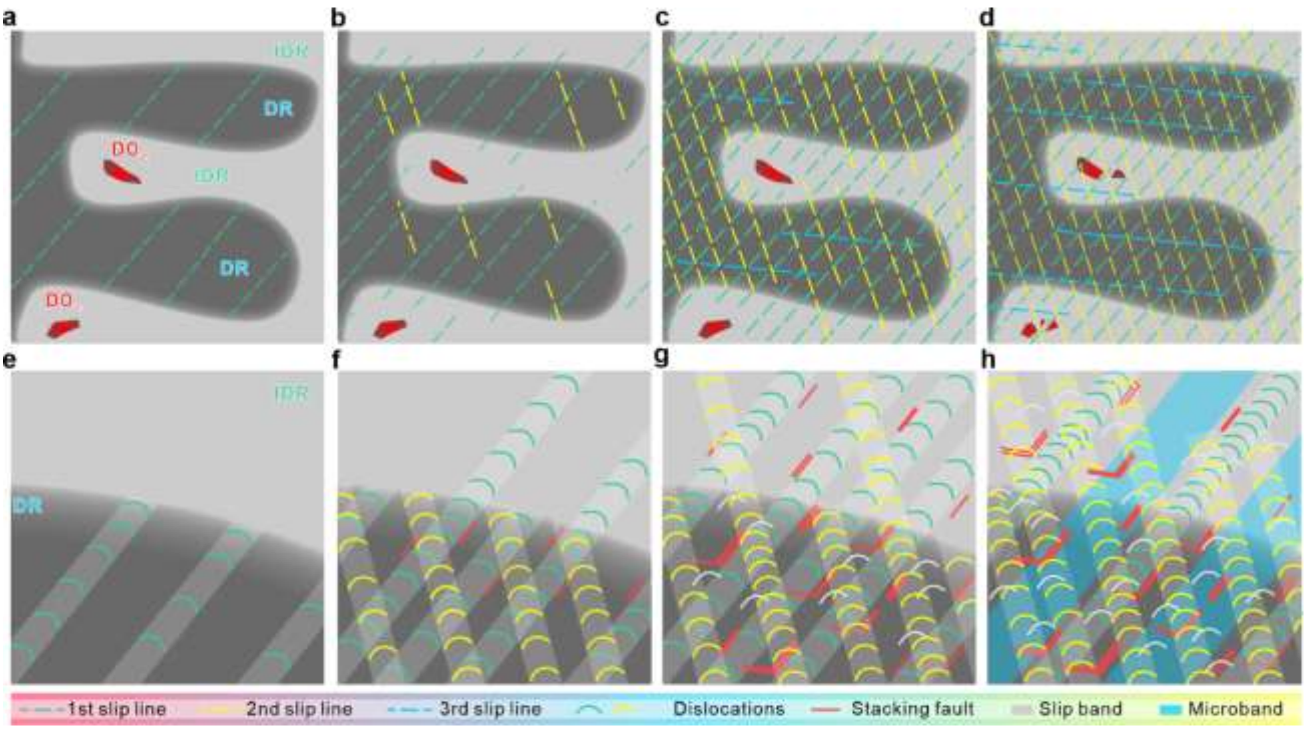

Figure S14 | Schematic diagram of deformation mechanisms spanning from the microscopic scale (a-d) to nanometer scale (e-h).

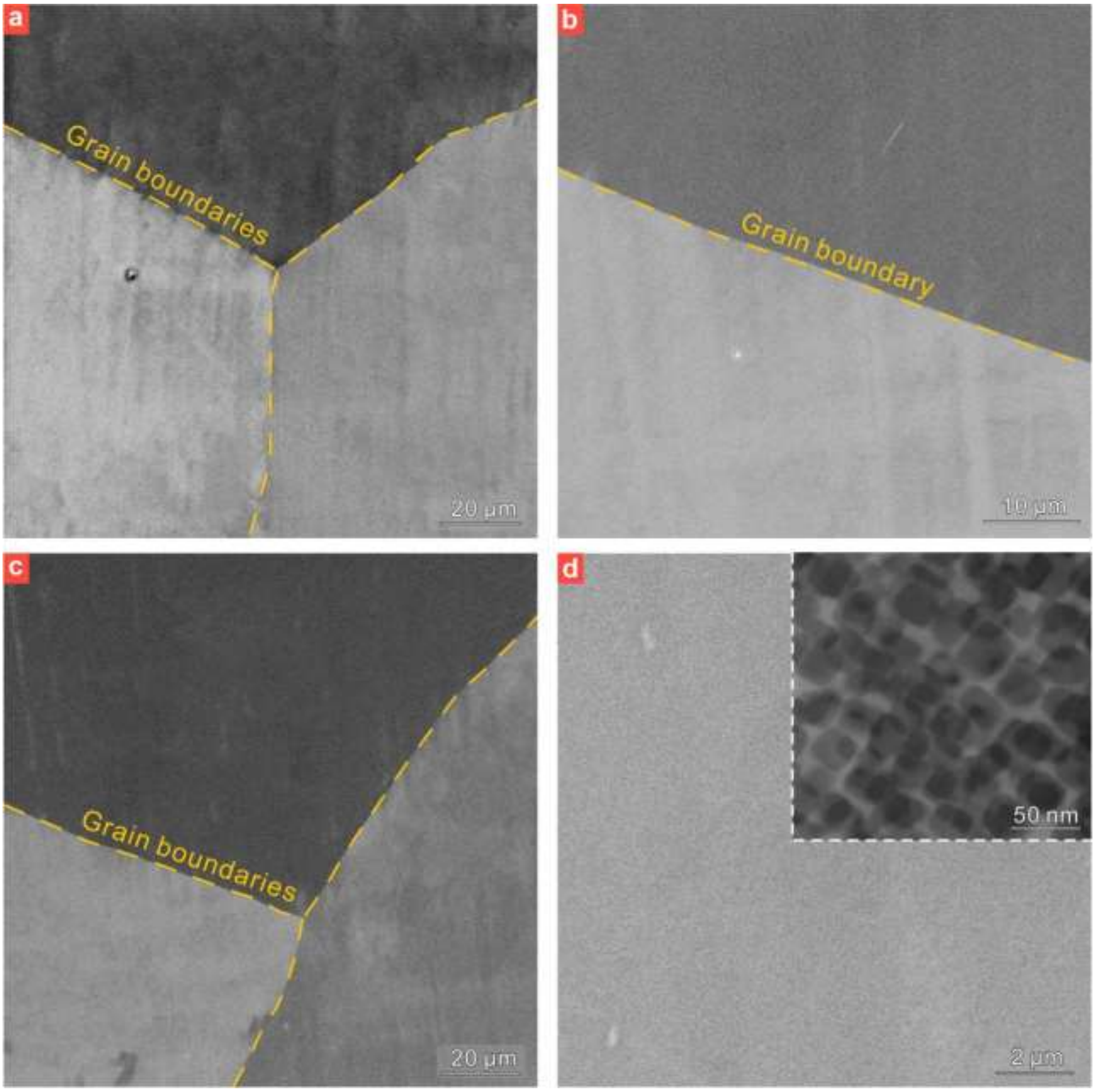

Figure S15 | Homogeneous microstructures after annealing and/or ageing. (**a, b**) SEM images with different magnifications revealing a homogeneous microstructure after high-temperature homogenization annealing at 1200 °C for 12 h. Sporadic undissolved $D0_{19}$ particles are observed in (**a, b**). (**c, d**) SEM images with different magnifications revealing a homogeneous microstructure after high-temperature homogenization annealing at 1200 °C for 12 h and aging at 800 °C for 4 h. The STEM inset shows dense nanoprecipitates after aging.

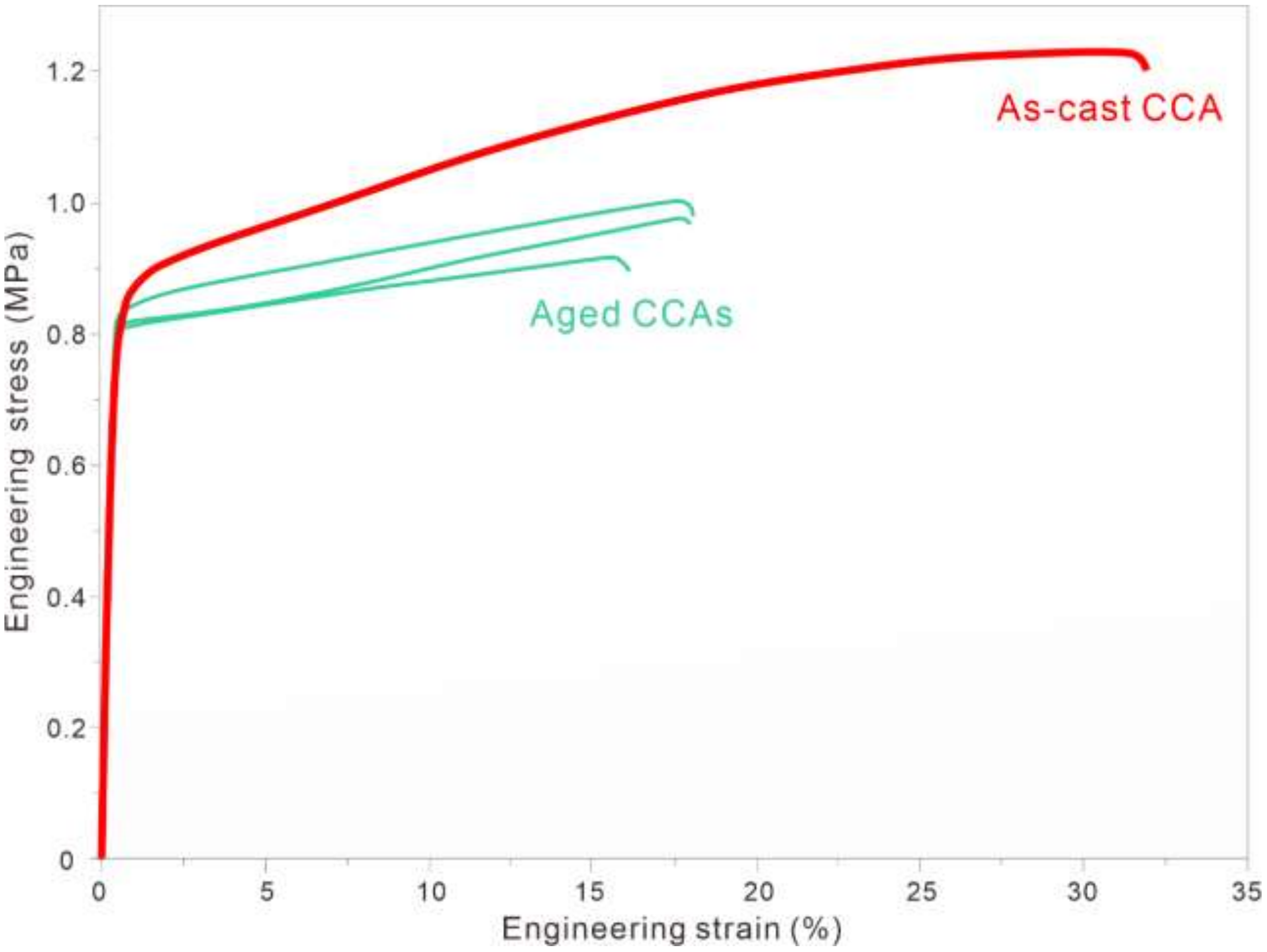

Figure S16 | Engineering stress-strain curves of as-cast CCA *vs* CCAs aged at 800 ℃ for 4 hrs.

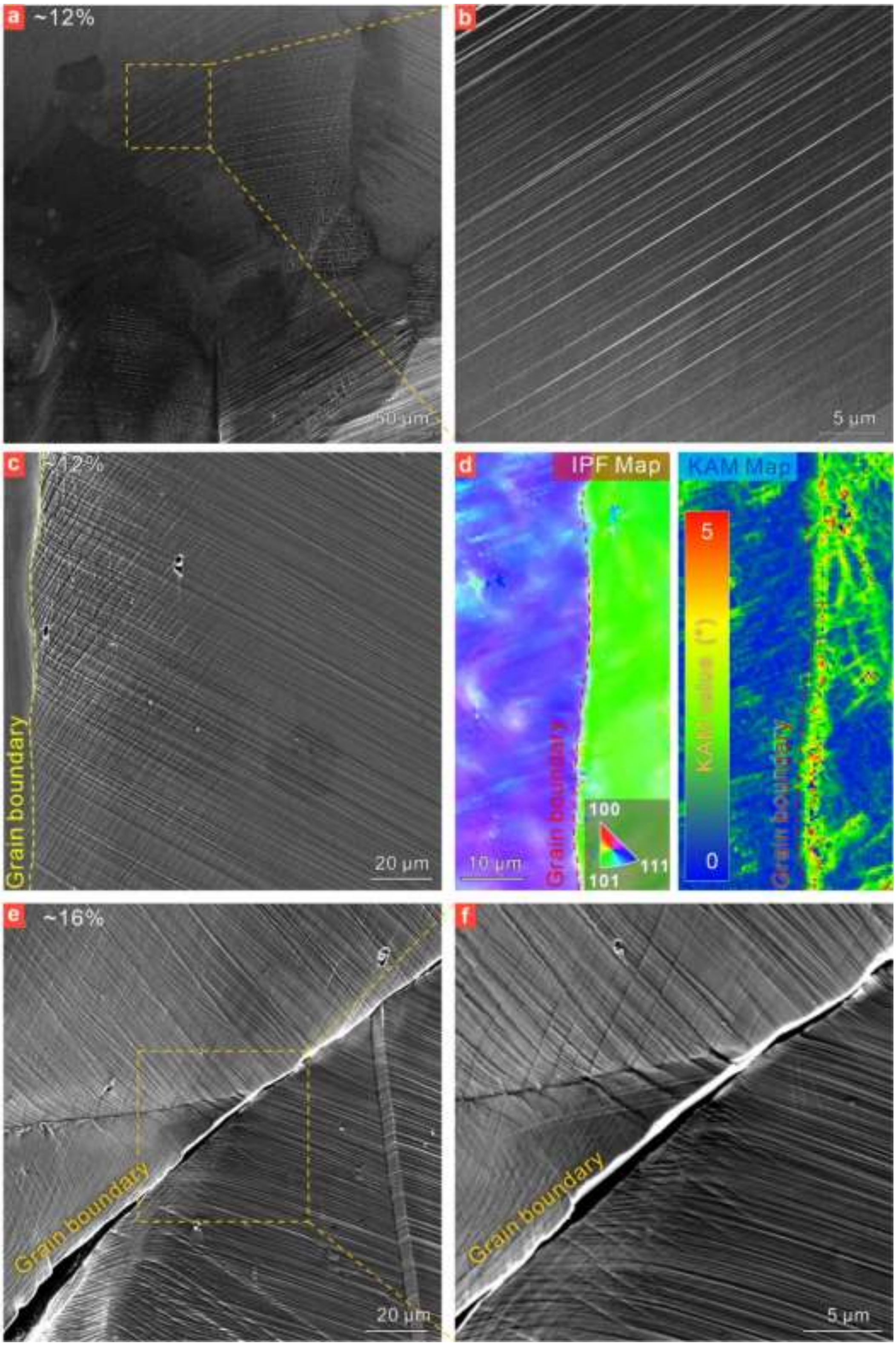

Figure S17 | SEM and EBSD images revealing deformation and failure mechanisms of aged CCAs. (**a, b**) Single-directional intragranular slip lines. (**c**) Dense and multi-directional slip lines rendering severe plastic deformation near grain boundaries. (**d**) Corresponding EBSD probing revealing higher KAM values near grain boundaries. (**e, f**) Grain-boundary cracking.

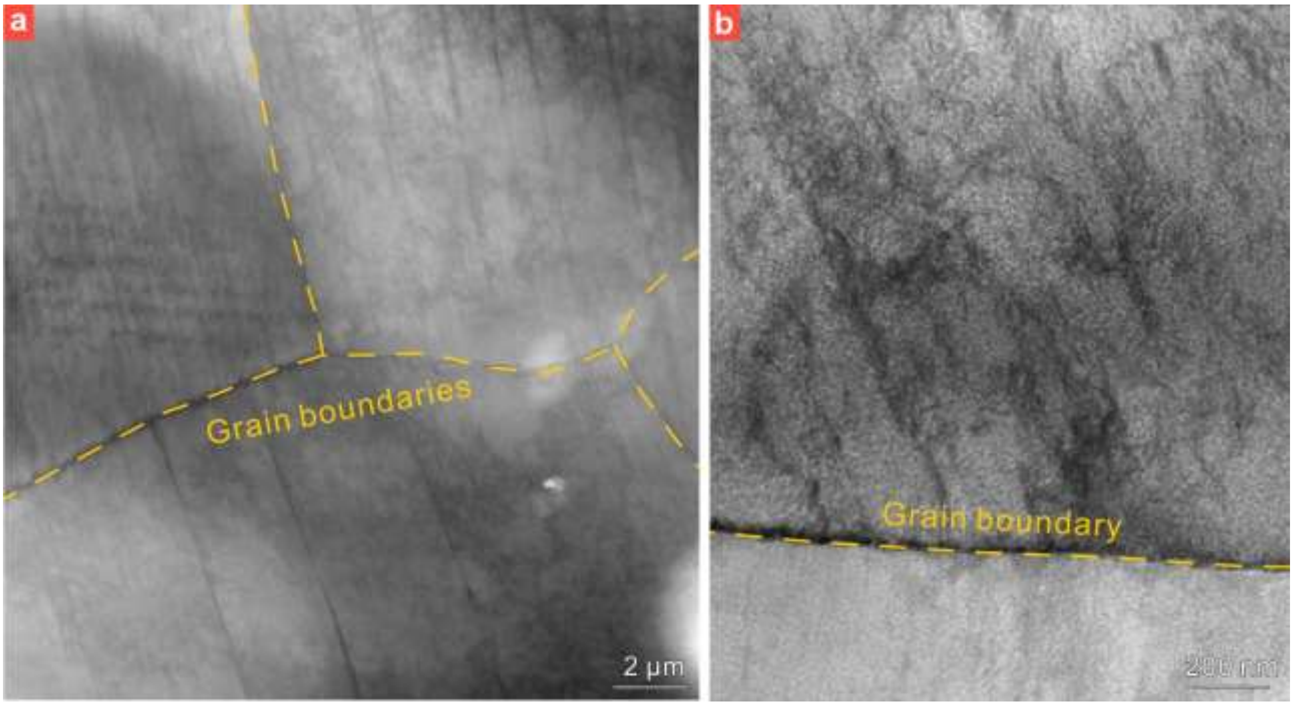

Figure S18 | (a, b) STEM images at different magnifications revealing that there is no significantly increased dislocation accumulation near grain boundaries compared to inside the grains.

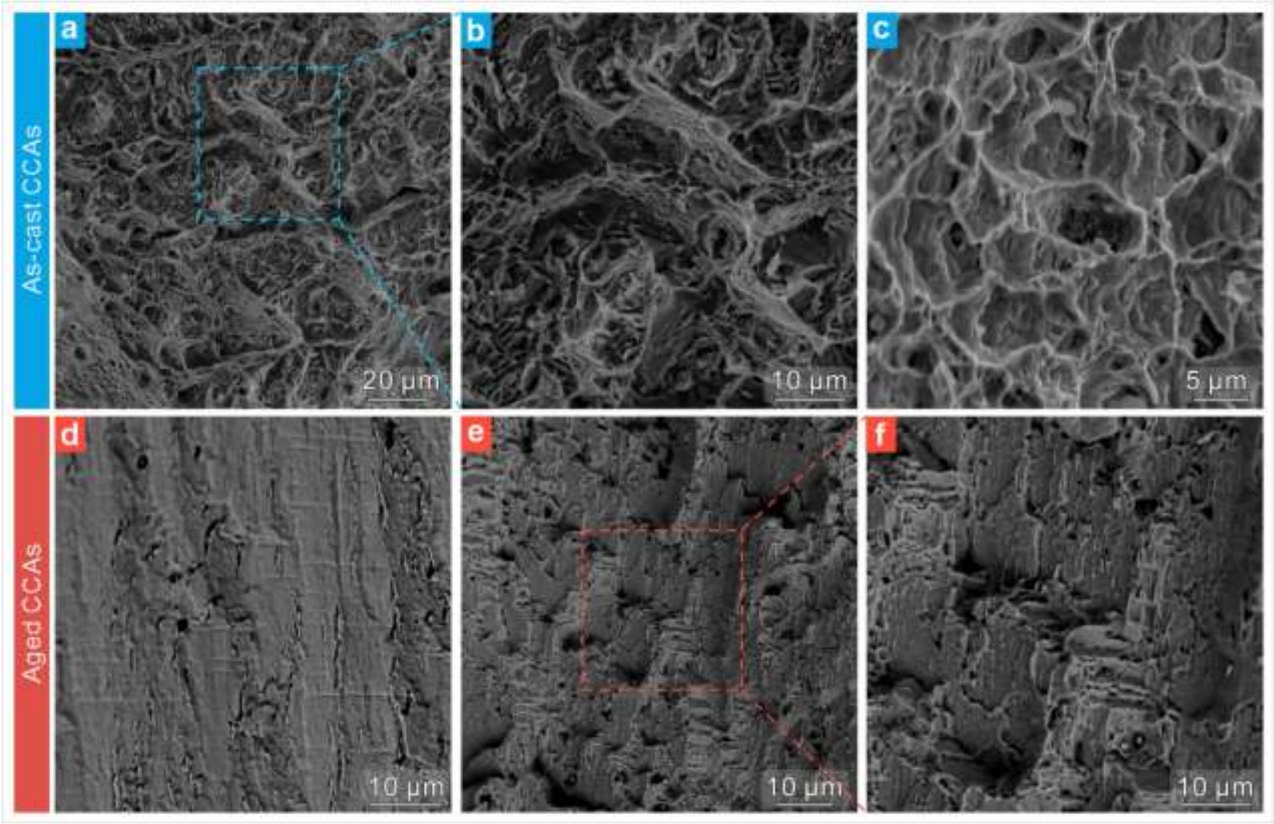

Figure S19 | SEM images revealing different fracture morphologies of (a-c) as-cast CCAs and (d-f) aged CCAs.

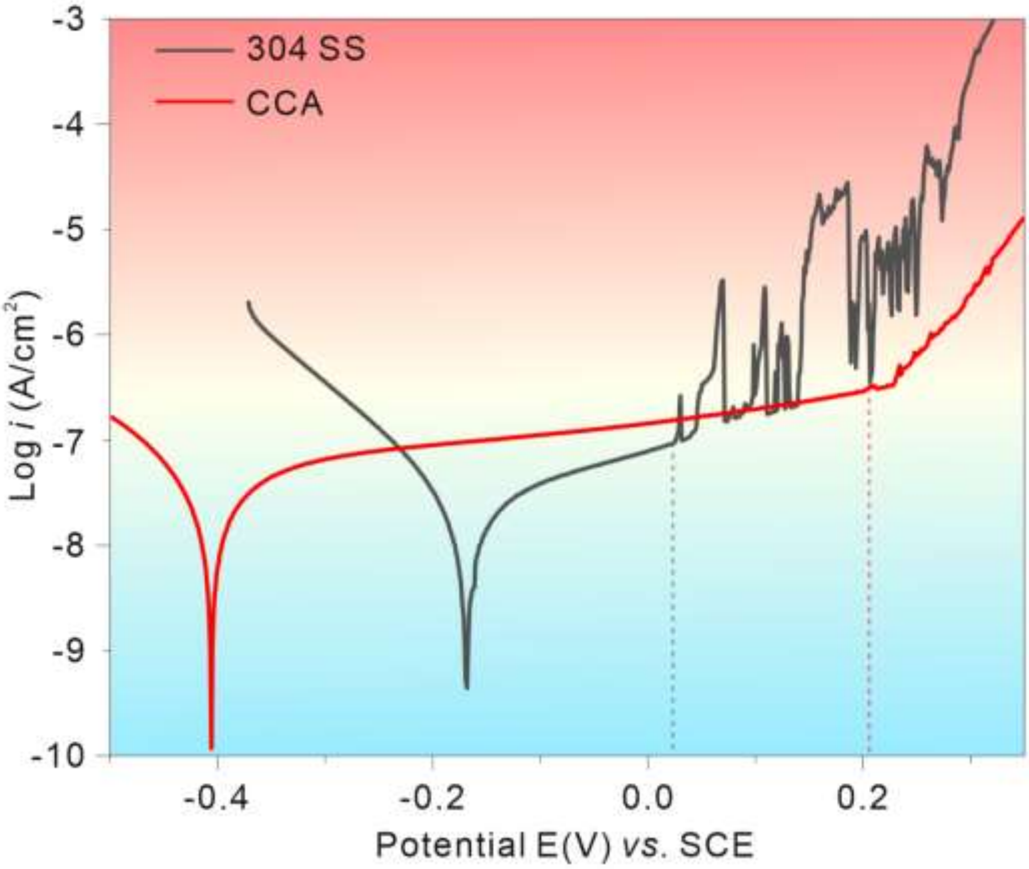

Figure S20 | Potentiodynamic polarization curves of the developed CCA and commercial 304 stainless steel (304 SS; a typical corrosion-resistant material) in 3.5 wt.% NaCl solution. The corrosion potential (E_{corr}) is employed to denote the propensity for corrosion to occur, whereas the pitting potential (E_p) typically signifies the potential at which the pitting corrosion commences. The difference between these two potentials ($\Delta E = E_p - E_{corr}$) is defined as the passivation range. Although E_{corr} of the CCA is relatively lower (about -400 mV_{SCE}), its pitting potential is comparable to that of 304 SS. Besides, it is noteworthy that the passivation range of the CCA is 637 mV_{SCE}, whereas that of 304 SS is 420 mV_{SCE}. The length of the passivation range serves as a significant indicator of both the stability and protective efficacy of the passive film formed on the material surface, whereby a longer passivation range typically suggests a stronger corrosion resistance. The CCAs we developed feature superior corrosion resistance, in addition to salient advantage in as-cast mechanical properties.

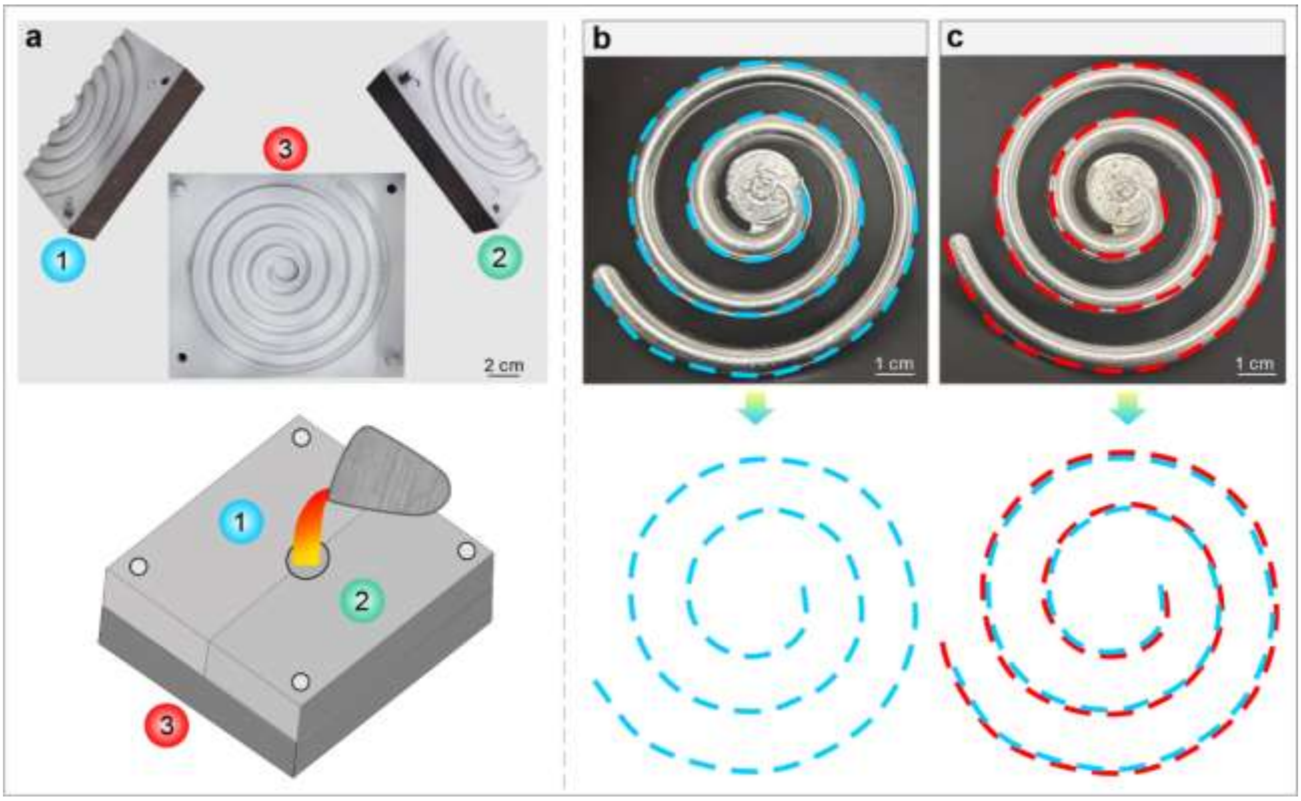

Figure S21 | Fluidity evaluation. (**a**) Optical images displaying three separation molds used in the fluidity evaluation. The schematic diagram in the bottom panel shows the casting process. (**b, c**) Optical images revealing that compared to the commercial 304 stainless steel (a common casting material) (**b**), our CCA in this work (**c**) exhibits a comparable solidification length, which is indicative of good fluidity and castability.

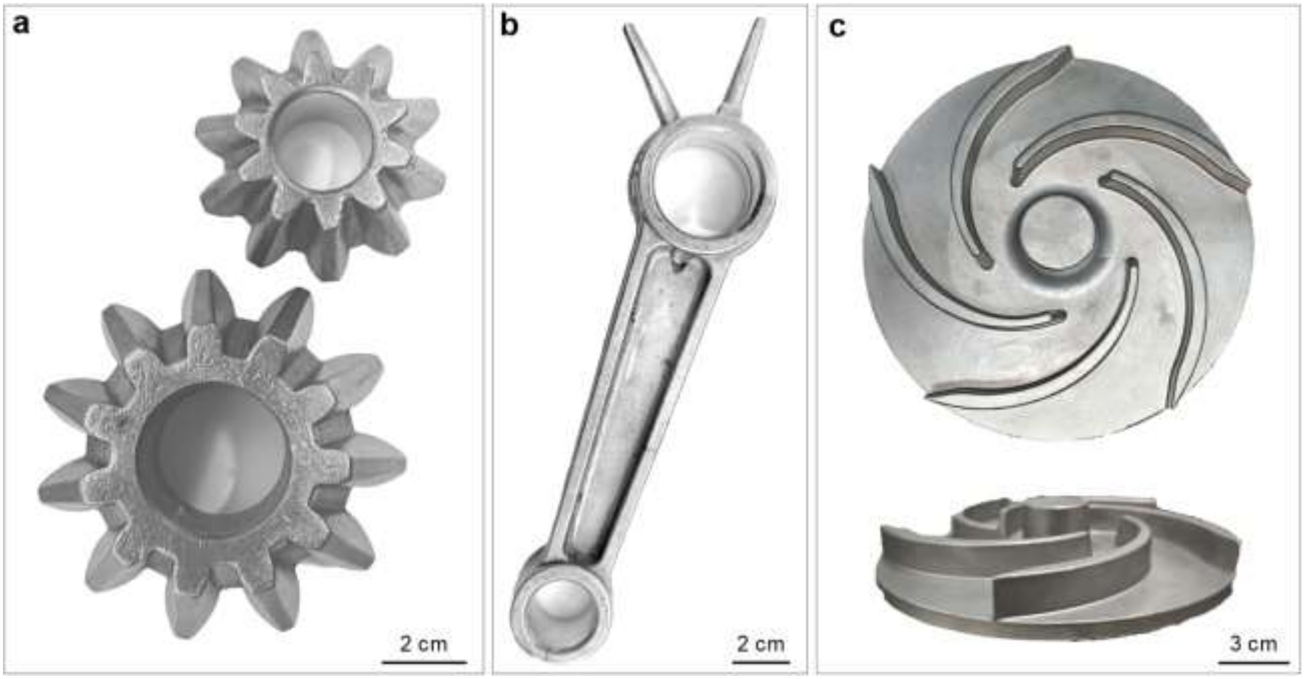

Figure S22 | Optical images of some casting parts. (**a**) Bevel gears. (**b**) Connecting rod. (**c**) Centrifugal pump impeller. These illustrated parts are in great demand in both civilian and military applications, both require excellent as-cast mechanical properties. In addition, the complex geometries obtained through directly casting alone can leave out downstream processing, such as welding and stamping, which pose additional risk of failure, in addition to being time-consuming and costly (as schematically illustrated in **Fig. 5**). Thus, this study is also highly competitive with expensive powder metallurgy and additive manufacturing (used to simultaneously produce complex geometries and high properties). Besides, our high-performance cast CCAs can also be used to produce launcher components, bearing shells, propulsion components, triggers, bolt catches, bolt carriers, barrels, sights, blank fire adapters, ejectors, rocket motor case, hinges, rotary joints, etc. Overall, the military has a big demand for casting parts. Among them, mechanically strong casting parts are particularly needed for weapons, tanks, warships, heavy-duty vehicles, etc. Such diverse usage scenarios, on the other hand, demand multi-purpose materials, which are currently highly sought after by metallurgists and material scientists. Finally, we emphasize once again that these developed casting materials are particularly suitable for those components that require rapid, near-net-shape forming preparation as well as high mechanical properties (but cannot be subject to post treatments). Such a ‘green’ achievement is beyond the pursuit for sheer property records, which all required downstream processing that increases, being often multi-step and subtractive, production time, expenses as well as greenhouse gas emissions.

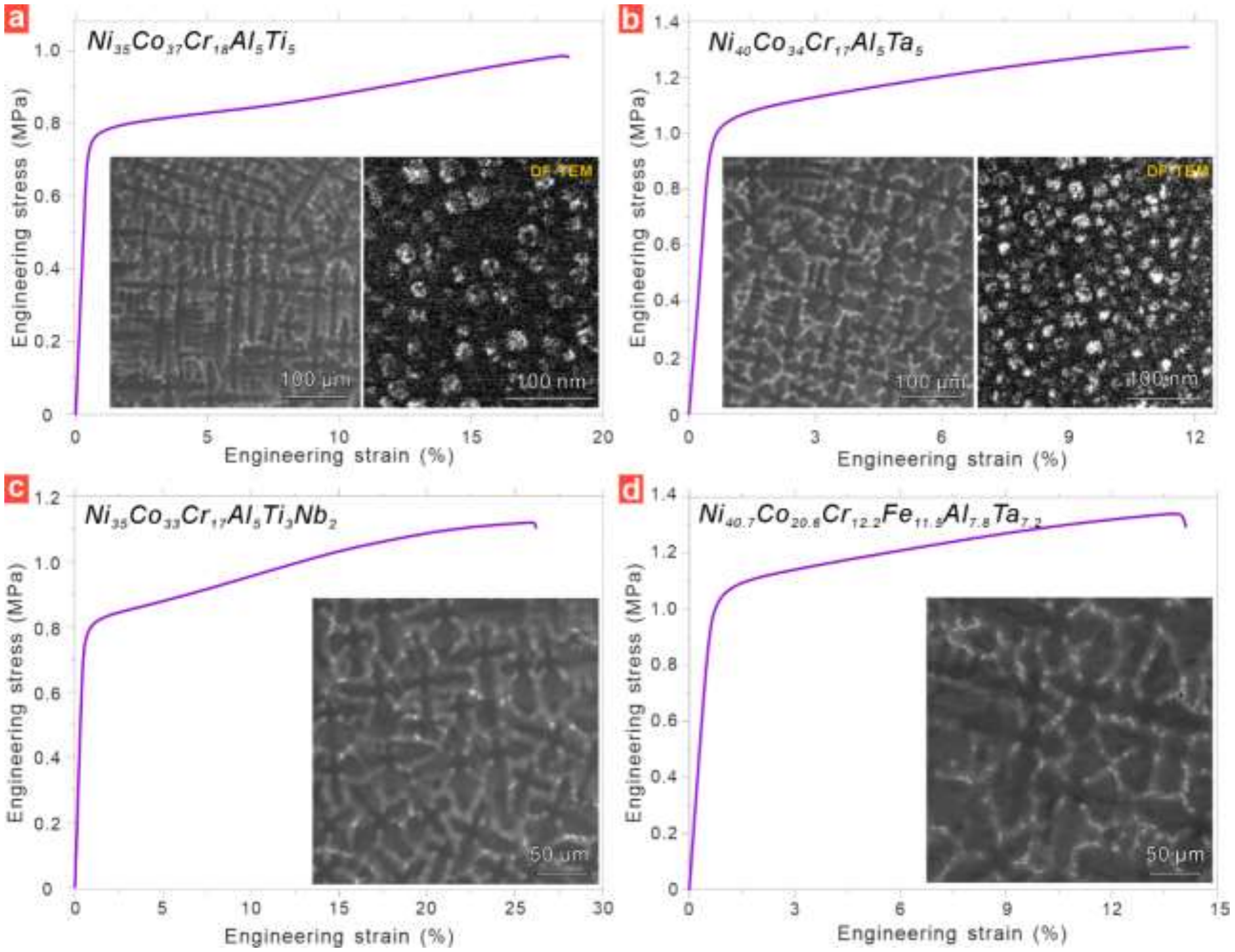

Figure S23 | Tensile stress-strain curves of four different *in situ* precipitation-hardened CCA systems with high as-cast strength−ductility combinations. The SEM insets (**a-d**) show corresponding as-cast, highly-branched dendrite microstructures. The dark-field (DF) TEM insets (**a, b**) show *in situ* nanoprecipitates after casting.

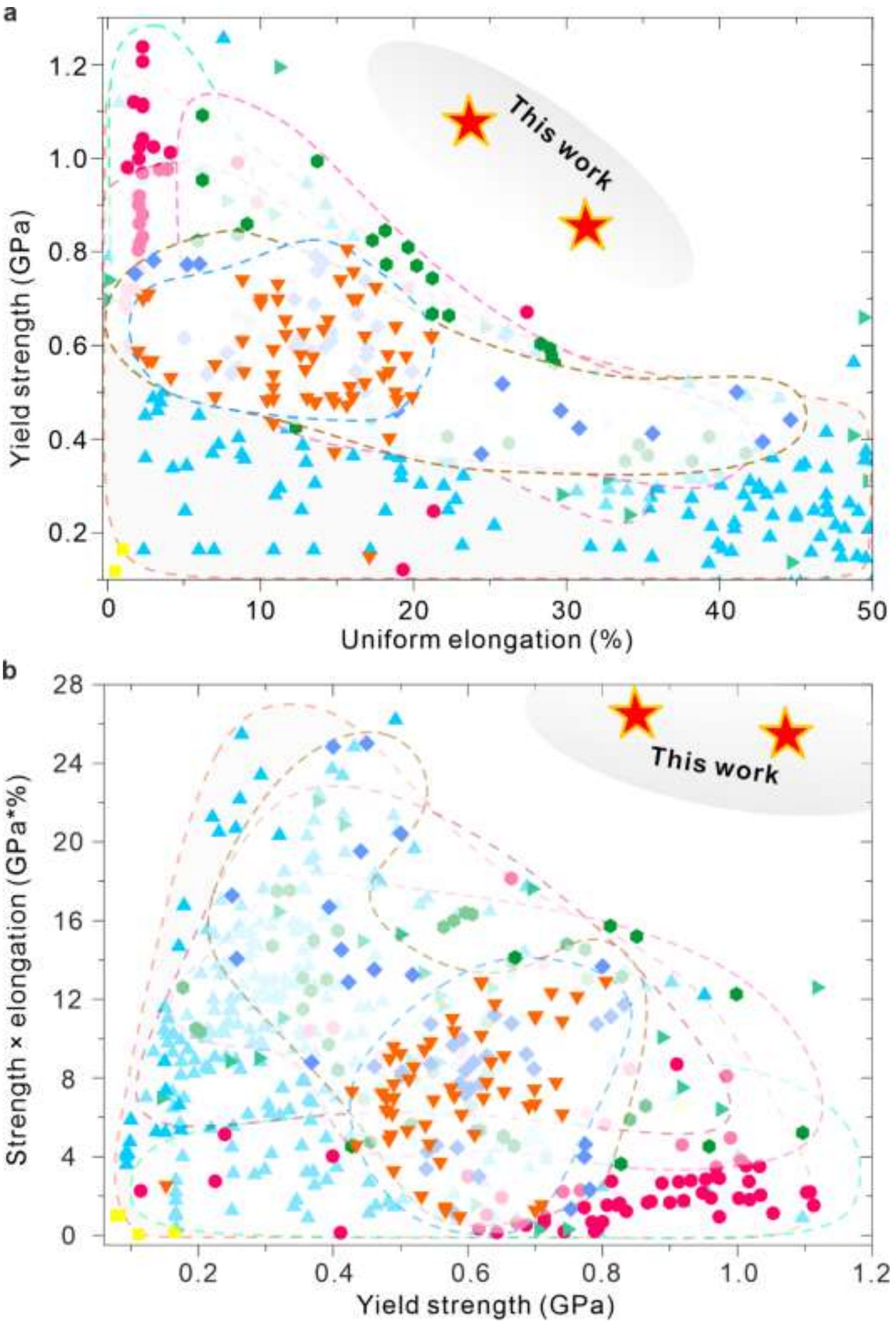

Figure S24 | (a, b) Two property comparison maps, showing (**a**) yield strength *versus* uniform elongation and (**b**) 'yield strength × uniform elongation' *versus* yield strength, in comparison with all previous as-cast CCAs. The detailed data and associated references of every symbol in (**a**, **b**) are given in **Table S3**.

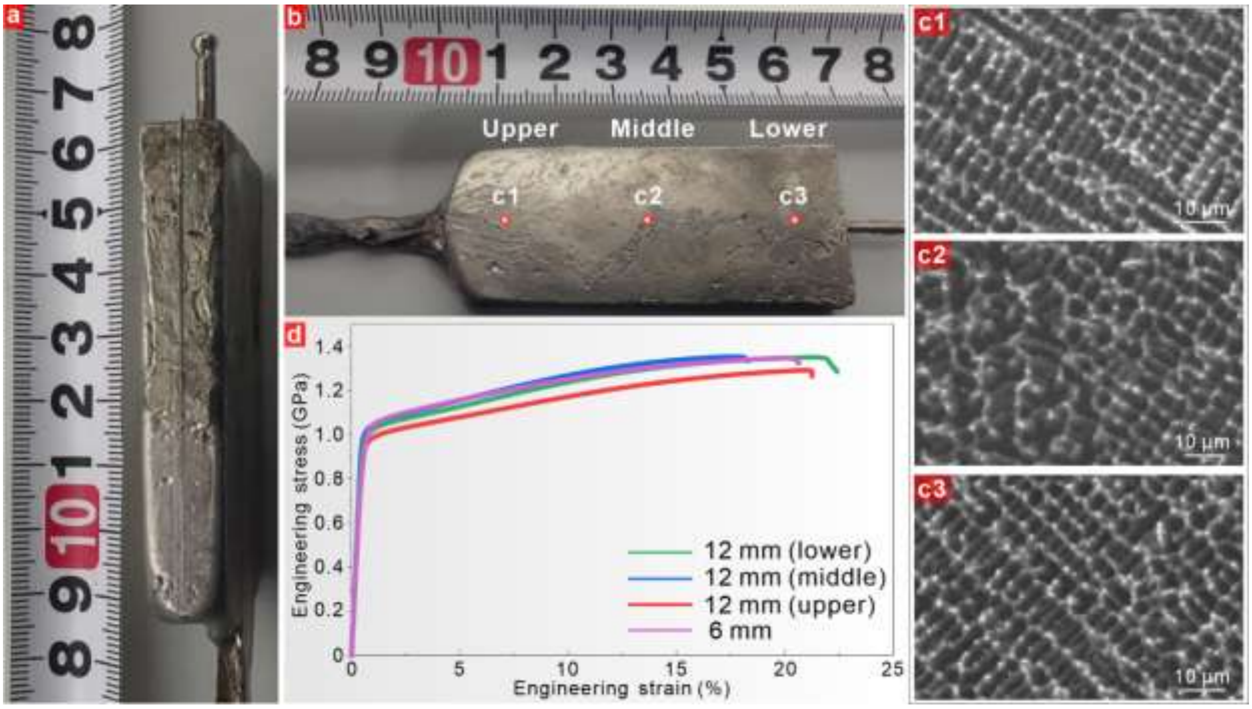

Figure S25 | Repeatable microstructures, and mechanical properties for smaples of a larger thickness. (**a, b**) Photographs of as-cast non-equiatomic NiCoCrAlTaZrB CCAs with a larger thickness of ~12 mm than that of ~6 mm in the main text. (**c**) SEM images showing uniform microstructures of the upper, middle and lower parts. (**c, d**) Comparable microstructures (**c**) and tensile properties (**d**) with that having a thickness of ~6 mm in the main text.

Table S3. A summary of tensile properties in Fig. 2c and d, including yield strength ($\sigma_{0.2}$), ultimate tensile strength (σ_{uts}), uniform elongation (ε_u), and corresponding references.

Composition	$\sigma_{0.2}$ (MPa)	σ_{uts} (MPa)	ε_u (%)	Ref
**HCP structure CCAs**				
$(TiZrHf)_{99}Cu_1$	767	910	10.2	1
$(TiZrHf)_{98}Cu_2$	803	959	8.9	
$(TiZrHf)_{97}Cu_3$	917	1036	7.2	
HoYGd	111	113	0.5	2
HoDyTb	32	197	10.1	
HoYGdTb	78	169	12.8	
HoDyGdTb	83	137	12.3	
HoDyYGdTb	165	204	1	
**BCC structure CCAs**				
$Al_{12.5}Ni_{21.875}Co_{21.875}Fe_{21.875}Cr_{21.875}$	598	930	15.6	3
$Al_{12.5}Ni_{17.5}Co_{17.5}Fe_{35}Cr_{17.5}$	991	1245	8.2	
$Nb_{40}Ti_{40}V_{20}$	650	700	0.3	4
$Al_{15}Nb_{40}Ti_{40}V_5$	750	775	0.3	
$Al_{20}Nb_{40}Ti_{40}$	795	800	0.3	
$Al_5Mo_5Nb_{36}Hf_{13}Ti_{41}$	680	683	1	5
$Al_5Mo_7Nb_{36}Hf_{13}Ti_{39}$	820	860	3.4	
$Ti_{37}V_{15}Nb_{22}Hf_{23}W_3$	980	1000	1	6
TiVNbTa	720	735	1.4	7
$TiVNbTaSi_{0.1}$	1120	1240	1.4	
$Ti_{35}Zr_{35}Nb_{20}Ta_5Mo_5$	968	1127	2	8
$Ti_{35}Zr_{35}Nb_{15}Ta_{10}Mo_5$	1041.5	1234	2	
$Ti_{35}Zr_{35}Nb_{10}Ta_{15}Mo_5$	1110	1127	2	
$(Zr_{0.5}Ti_{0.35}Nb_{0.15})_{90}Al_{10}$	1012	1097	3.8	9
$(Zr_{0.5}Ti_{0.35}Nb_{0.15})_{80}Al_{20}$	693	693	0.8	
$(TiZrNbTa)_{99.7}N_{0.3}$	1115	1152	2	10
$(TiZrNbTa)_{99.4}N_{0.6}$	1196	1270	2	
$(TiZrNbTa)_{99.1}N_{0.9}$	1242	1242	2.3	
$HfTa_{0.2}TiZr$	1206	1315	2	11
$HfNb_{0.15}Ta_{0.2}TiZr$	406	1004	10	
$HfNb_{0.2}Ta_{0.2}TiZr$	607	983	5	
$HfNb_{0.25}Ta_{0.2}TiZr$	748	843	3	
$(TiV)_{88}Al_{12}$	418	418	0.4	12
$(TiV)_{88}Cr_9Al_3$	623	623	0.6	
$(TiV)_{88}Cr_6Al_6$	791	791	0.9	
$(TiV)_{94}Cr_3Al_3$	807	807	0.9	
$(TiV)_{91}Cr_{4.5}Al_{4.5}$	784	812	8	
$(TiV)_{88}Cr_3Al_9$	955	970	2.3	

$(TiV)_{88}Cr_{12}$	1024	1070	2.7	
$Ti_{60}Al_{6}(VCrNb)_{34}$	895	1115	3	13
$Ti_{60}Al_{8}(VCrNb)_{32}$	920	1146	3	
$Ti_{60}Al_{10}(VCrNb)_{30}$	938	1129	3	
$Ti_{60}Al_{12}(VCrNb)_{28}$	960	1072	3	
$Ti_{35}Zr_{15}Nb_{25}Ta_{25}$	842	873	1.5	14
VNbTa	925	1050	5	15
$V_{0.5}Nb_{0.5}ZrTi$	832	933	2	16
$Ti_{38}V_{15}Nb_{23}Hf_{24}$	774	792	3	17
$Zr_{50}V_{35}Ti_{15}$	657	755	3	18
HfNbTaTi	800	820	0.5	19
HfNbTaZr	1025	1070	1.8	
HfNbTiZr	635	670	1.7	
HfTaTiZr	1060	1080	1.1	
NbTaTiZr	975	1045	3.1	
TiZrVNb	1040	1444	3.4	20
$Ti_{1.5}ZrVNb$	1020	1404	3.5	
$Ti_{2}ZrVNb$	975	1303	3.6	
$Ti_{35}Zr_{27.5}Hf_{27.5}Nb_{5}Ta_{5}$	121	574	19	21
$Ti_{38}Zr_{25}Hf_{25}Ta_{10}Sn_{2}$	407	925	26	
$Ti_{38}Zr_{25}Hf_{25}Ta_{7}Sn_{5}$	232	409	12	
$Ti_{65}(AlCrNb)_{35}$	997	1154	5	22
NbTa	246	304	21	23
TiNbTa	478	514	19	
HfNbTa	847	889	10	
TiHfNbTa	663	709	11	
$Ti_{35}Zr_{27.5}Hf_{27.5}Nb_{5}Ta_{5}$	540	995	17	24
TaHfZrTi	1378	1498	2.4	25
$Ta_{0.6}HfZrTi$	719	1093	19.3	
$Ta_{0.5}HfZrTi$	671	1103	27.1	
$Ta_{0.4}HfZrTi$	371	1109	28.1	
$Hf_{0.5}Nb_{0.5}Ta_{0.5}Ti_{1.5}Zr$	879	969	2	26
TiHfZrTaNb	810	890	7	27
$Ti_{40}Zr_{25}Nb_{25}Ta_{5}Al_{5}$	960	1020	2	28
$Al_{0.8}CrNiMn_{2}Fe_{2.5}$	907	1328	9.6	29
$Ti_{1.6}ZrNbAl_{0.4}$	865	869	2	30
$Ti_{1.6}ZrNbAl_{0.2}V_{0.2}$	806	816	2	
$Ti_{1.6}ZrNbAlV_{0.4}$	770	779	2	
$Ti_{1.6}ZrNbAl_{0.3}$	800	830	2	31
$Ti_{1.6}ZrNbAl_{0.4}$	820	890	2	
$Ti_{1.6}ZrNbAl_{0.5}$	880	900	2	
**FCC structure CCAs**				
$CrMnFeCoNi_{0.8}$	240	527	62.6	32

CrMnFeCoNi$_{0.8}$ + 2.5 vol%TiC	362	792	52.5	
CrMnFeCoNi$_{0.8}$ + 5.0 vol%TiC	451	874	39.3	
CrMnFeCoNi$_{0.8}$ + 7.5 vol%TiC	517	926	38	
CrMnFeCoNi$_{0.8}$ + 10 vol%TiC	449	804	25.8	
$Ni_2CrFeAl_{0.3}Ti_{0.1}$	230	671	74	33
$Ni_2CrFeAl_{0.3}Ti_{0.2}$	467	917	38.4	
$Ni_{36}Co_{30}Cr_{11}Fe_{11}Al_{10}Nb_2$	536	889	32	34
$Ni_{36}Co_{30}Cr_{11}Fe_{11}Al_8Nb_4$	632	902	14.8	
$Ni_{36}Co_{30}Cr_{11}Fe_{11}Al_6Nb_6$	713	893	5.4	
$Ni_{36}Co_{30}Cr_{11}Fe_{11}Al_4Nb_8$	812	984	4.6	
$Ni_{36}Co_{30}Cr_{11}Fe_{11}Al_2Nb_{10}$	997	1134	4	
VCoNi	383	634	42.3	35
$(VCoNi)_{97}Al_3$	491	813	41.8	
$(VCoNi)_{93}Al_7$	629	1214	26.2	
CrCoNiAl	263	543	58.2	36
$CrCoNiAl_{0.2}$	358	608	56.7	
$CrCoNiAl_{0.4}$	563	972	48.8	
$CrCoNiAl_{0.6}$	594	1105	12.2	
CoCrNi	352	799	50.2	37
$Ni_{0.6}CoFe_{1.4}$	342	792	5.2	38
$Ni_{0.6}CoFe_{1.4}Nb_{0.05}$	248	573	22.3	
$Ni_{0.6}CoFe_{1.4}Nb_{0.08}$	426	748	16.4	
$Ni_{0.6}CoFe_{1.4}Nb_{0.1}$	440	838	11.8	
$Al_{0.3}CoCrFeNiMn$	220	427	69.4	39
CoCrFeNiMn	368	664	52.4	40
$Al_{0.5}CoFeNiC_{0.1}$	1115	1212	8	41
$Al_{0.5}CoFeNiC_{0.1}Mn_{0.3}$	1000	1281	23	
$Al_{0.5}CoFeNiC_{0.1}Mn_{0.6}$	673	1153	25.8	
$Al_{0.4}Cr_{0.7}Fe_{0.5}Ni_2V_{0.2}$	459	687	30.9	42
$Al_{0.4}Cr_{0.7}FeNi_2V_{0.2}$	367	541	40.1	
$Al_{0.4}Cr_{0.7}Fe_2Ni_2V_{0.2}$	151	318	73	
$(CrMnFeCoNi)_{98}V_2$	275	552	64	43
$(CrMnFeCoNi)_{98}Ti_2$	310	635	54	
$(CrMnFeCoNi)_{98}Nb_2$	385	640	24	
$CrFeNiMn_{0.5}Cu_{0.5}$	582	1020	28	44
$Al_{0.3}CoCrFeNi$	167	500	88	45
$Al_{0.3}CoCrFeNiTi_{0.1}$	289	635	81	
$Al_{0.3}CoCrFeNiTi_{0.2}$	414	758	47	
$Al_{0.3}CoCrFeNiTi_{0.3}$	578	852	15	
$Al_{0.3}CoCrFeNiTi_{0.4}$	698	922	7	
$Al_{0.3}CoCrFeNiTi_{0.5}$	487	487	3	
$Al_{0.3}CoCrFeNiSi_{0.1}$	217	489	98	46
$Al_{0.3}CoCrFeNiSi_{0.2}$	258	591	86	

$Al_{0.3}CoCrFeNiSi_{0.3}$	428	717	58	
$Al_{0.3}CoCrFeNiSi_{0.4}$	625	774	18	
$Al_{0.3}CoCrFeNiSi_{0.5}$	713	748	6	
$CoCrFeNiMo_{0.4}$	420	646	24.1	47
$CoCrFeNiNb_{0.1}Mo_{0.3}$	426	714	17.4	
$CoCrFeNiNb_{0.2}Mo_{0.2}$	510	763	10	
$CoCrFeNiNb_{0.3}Mo_{0.1}$	692	894	7.5	
$CoCrFeNiNb_{0.4}$	618	645	4.4	
$Co_{30}Cr_{30}(FeNi)_{40}$	227	578	90.3	48
$Co_{30}Cr_{30}(FeNi)_{38}Mo_2$	261	616	97.6	
$Co_{30}Cr_{30}(FeNi)_{36}Mo_4$	327	689	102.4	
$Co_{30}Cr_{30}(FeNi)_{34}Mo_6$	486	770	43.9	
$Co_{30}Cr_{30}(FeNi)_{32}Mo_8$	681	730	6.9	
$Co_{30}Cr_{30}(FeNi)_{30}Mo_{10}$	688	709	5	
$CrFeNiCuSi_{0.2}$	262	458	48.5	49
$CrFeNiCuSi_{0.3}$	305	502	13.6	
$Co_{30}Cr_{30}Fe_{20}Ni_{20}$	267	613	83.3	50
$Co_{36}Fe_{36}Cr_{18}Ni_{10}$	147	509	35.6	51
$Fe_{40}Cr_{15}Co_{15}Mn_{10}Ni_{20}$	158	420	58.8	52
$Fe_{40}Cr_{15}Co_{10}Mn_4Ni_{20}Al_{11}$	218	507	55	
$Fe_{40}Cr_{15}Co_{10}Mn_5Ni_{20}Mo_{10}$	246	346	5.1	
$AlCo_4Cr_{1.5}Fe_{1.5}Ni_5$	230	533	57.5	53
$Al_{1.2}Co_4Cr_{1.5}Fe_{1.5}Ni_5$	284	577	51.7	
$Al_{1.4}Co_4Cr_{1.5}Fe_{1.5}Ni_5$	306	609	51.3	
$Al_{1.6}Co_4Cr_{1.5}Fe_{1.5}Ni_5$	346	678	45.6	
$Al_{1.8}Co_4Cr_{1.5}Fe_{1.5}Ni_5$	369	775	34.5	
$Al_2Co_4Cr_{1.5}Fe_{1.5}Ni_5$	401	834	31.6	
$CrFe_2Ni_2Mo_{0.3}$	237	548	57	54
$Ni_{46}Co_{24}Fe_{24}Al_3Ti_3$	525	935	32	55
$Al_8Co_{30}Cr_{18}Fe_9Ni_{31}Nb_4$	568	765	/	56
$Al_8Co_{30}Cr_{18}Fe_9Ni_{31}Nb_2Ti_2$	494	738	/	
$Al_8Co_{30}Cr_{18}Fe_9Ni_{31}Ti_4$	486	844	/	
MnFeCoCu	313	518	38.2	57
$MnFeCoCuNi_{0.5}$	344	546	39.4	
MnFeCoCuNi	301	499	40.1	
$MnFeCoCuNi_{1.5}$	216	480	47.6	
$Cr_7Mn_{25}Co_9Ni_{23}Cu_{36}$	390	692	33.6	58
$Fe_{50}Mn_{27}Ni_{10}Cr_{13}$	90	250	40	59
$(Fe_{50}Mn_{27}Ni_{10}Cr_{13})_{98}Mo_2$	215	387	25.3	
$(Fe_{50}Mn_{27}Ni_{10}Cr_{13})_{96}Mo_4$	295	455	11.3	
$(Fe_{50}Mn_{27}Ni_{10}Cr_{13})_{94}Mo_6$	450	465	2.5	
$Ni_{3.5}Co_3Cr_{1.5}$	147	447	78.8	60
$(Ni_{3.5}Co_3Cr_{1.5})_{90}Al_5Ti_5$	792	1003	38.2	

$Al_{0.3}FeCoNi$	221	486	41	61
$Al_{0.3}Cr_{0.5}FeCoNi$	233	504	45	
$Al_{0.3}CrFeCoNi$	259	559	47	
$Al_{0.3}Cr_{1.5}FeCoNi$	294	640	44	
$Al_{0.3}Cr_{1.7}FeCoNi$	338	742	28	
$Al_{0.3}Cr_2FeCoNi$	546	1078	11	
$Fe_{53}Mn_{29}Co_9Cr_9CSi$	368	756	57.3	62
$Fe_4CrNi(AlTi)_{0.2}$	173	425	23.2	63
$Fe_4CrNi(AlTi)_{0.6}$	386	858	12.8	
$Fe_4CrNi(AlTi)_{1.0}$	1118	1172	0.8	
$Fe_{40}Mn_{40}Ni_{10}Cr_{10}$	170	446	47.8	64
$(Fe_{40}Mn_{40}Ni_{10}Cr_{10})_{95}Al_5$	190	477	43.3	
$(Fe_{40}Mn_{40}Ni_{10}Cr_{10})_{92}Al_8$	259	595	32.6	
$(Fe_{40}Mn_{40}Ni_{10}Cr_{10})_{90}Al_{10}$	531	845	20.7	
$CrMnFeCoNi_{0.2}$	460	460	3.3	65
$CrMnFeCoNi_{0.4}$	194	508	52.7	
$CrMnFeCoNi_{0.6}$	247	556	55.8	
$CrMnFeCoNi_{0.8}$	233	529	59	
$CrMnFeCoNi_{0.8}Nb_{0.1}$	359	614	34.6	
$CrMnFeCoNi_{0.8}Nb_{0.2}$	509	662	13.7	
$CrMnFeCoNi_{0.8}Nb_{0.3}$	602	743	5	
$(Al_{7.5}Co_{21.9}Cr_{10.9}Ti_{5.0}Fe_{21.9}Ni_{32.8})_{99.5}Cu_{0.5}$	662	1000	20	66
$(Al_{7.5}Co_{21.9}Cr_{10.9}Ti_{5.0}Fe_{21.9}Ni_{32.8})_{97.5}Cu_{2.5}$	676	953	16.5	
$(Al_{7.5}Co_{21.9}Cr_{10.9}Ti_{5.0}Fe_{21.9}Ni_{32.8})_{95}Cu_5$	644	907	13.1	
$FeMnCrNiCo_{0.95}C_{0.05}$	287	605	55.4	67
$Al_{0.6}CoCrFeNi_2Mo_{0.08}V_{0.04}$	400	683	39	68
$Ni_2Co_1Fe_1V_{0.5}Mo_{0.2}$	324	578	57.1	69
$Al_{0.3}CoCr_3Fe_5Ni$	831	1019	16.1	70
$Y_{0.01}Al_{0.3}CoCr_3Fe_5Ni$	404	830	41.5	
$Y_{0.03}Al_{0.3}CoCr_3Fe_5Ni$	437	805	26.5	
$Y_{0.01}Al_{0.4}CoCr_3Fe_5Ni$	684	1003	25.9	
$Fe_{50}Mn_{27}Ni_{10}Cr_{13}$	90	250	40	71
$Al_{0.5}Cr_{0.9}FeNi_{2.5}V_{0.2}$	281	476	43.1	72
CoCrCuMnNi	458	742	40	
CoCrFeNi	151	446	64.8	73
$CoCrFeNiW_{0.2}$	255	476	42	
$CoCrFeNiW_{0.4}$	316	691	33.1	
CoCrFeMnNi-0.5N	220	530	52	74
CoCrFeMnNi-1N	275	610	54	
CoCrFeMnNi-1.5N	395	765	60	
$CoCrFeNi_{2.1}Nb_{0.2}$	169	522	33.5	75
$CoCrFeNi_{2.1}Nb_{0.4}$	628	814	6.1	
$CoCrFeMnNiSn_{0.03}$	257	467	61.1	76

$CoCrFeMnNiSn_{0.07}$	247	487	46.2	
$CoCrFeMnNiSn_{0.1}$	351	487	22.4	
CoCrFeNi	240	464	45	77
$CoCrFeNiHf_{0.1}$	350	640	29	
$CoCrFeNiHf_{0.2}$	450	616	6	
FeCoNiCu	359	427	2.5	78
$Fe_{1.5}CoNiCu$	248	392	12.7	
$Fe_2CoNiCu$	303	474	20.5	
$Fe_{2.5}CoNiCu$	367	639	8.8	
$Fe_3CoNiCu$	576	576	3.6	
$Al_{0.4}CrMnFeCoNi$	238	626	38	79
$Al_{0.5}CrMnFeCoNi$	293	699	32.8	
$Al_{0.6}CrMnFeCoNi$	370	800	27.9	
$Fe_{67}Co_{8.25}Cr_{8.25}Ni_{8.25}Mn_{8.25}$	150	625	68	80
$Fe_{24}Co_{23}Ni_{24}Cr_{23}Ti_2Al_4$	300	418	22	81
$Al_{0.5}Co_{1.5}CrFeNi_{1.5}$	180	453	62	82
$Fe_{53.6}Co_{8.8}Mn_{28.1}Cr_{8.6}C_{0.46}Si_{0.44}$	340	670	45.3	83
$Ni_{28}Co_{28}Fe_{21}Cr_{15}Al_4Ti_4$	289	552	34	84
$(FeCoNi)_{81}Cr_9Al_8Ti_1Nb_1$	280	701	47	85
$Co_{34.5}Cr_{30}Ni_{26.5}Al_{5.4}W_{3.6}$	311	647	52	86
FeCrCuMnNi	610	950	14.4	87
$Al_5Cr_{12}Fe_{35}Mn_{28}Ni_{20}$	243	547	47.1	88
$Al_{10}Cr_{12}Fe_{35}Mn_{23}Ni_{20}$	292	637	42.5	
$Fe_{40}Co_{15}Cr_{15}Ni_{15}Mn_{15}$	234	498	57	89
$Fe_{60}Co_{10}Cr_{10}Ni_{10}Mn_{10}$	200	451	66.5	
$Fe_{62}Co_{9.5}Cr_{9.5}Ni_{9.5}Mn_{9.5}$	175	494	95.8	
$Fe_{67}Co_{8.25}Cr_{8.25}Ni_{8.25}Mn_{8.25}$	145	606	71.8	
$Fe_{38.5}Mn_{20}Co_{20}Cr_{15}Si_5Cu_{1.5}$	318	594	34.3	90
$FeCoNiCr_{0.2}Si_{0.2}$	225	732	61	91
$Fe_{40.4}Ni_{11.3}Mn_{34.8}Al_{7.5}Cr_6$	159	535	40.8	92
$Fe_{40.4}Ni_{11.3}Mn_{34.8}Al_{7.5}Cr_6$-1.1C	355	1174	49.5	
$CoCrFeNi_{2.1}$	130	280	71.7	93
$CoCrFeNi_{2.1}Nb_{0.2}$	170	537	31.6	
$CoCrFeNi_{2.1}Nb_{0.4}$	640	840	7	
$Al_{0.5}CoCrFeNi$	325	390	40	94
$AlNi_2Co_2Fe_{1.5}Cr_{1.5}$	346	713	30	95
$CoCrFeNiMo_{0.15}$	489	895	53.6	96
$FeCoNiCrTi_{0.2}$	702	1240	36	97
$Ni_6Cr_4WFe_9Ti$	369	650	6.9	98
$V_{10}Cr_{10}Fe_{45}Co_{20}Ni_{15}$	294	626	36	99
$CoFeNi_2V_{0.5}Mo_{0.2}$	252	547	82.1	100
$CoCrFeNiMnC_{0.05}$	259	574	59.3	101
$CoCrFeNiMnC_{0.1}$	337	553	47	

$CoCrFeNiMnC_{0.15}$	365	619	18.5	
$CoCrFeNiMnC_{0.2}$	422	734	11.8	
$Ni_{48.7}Co_{17.5}Cr_{10}Fe_{9.3}Al_{7.5}Ti_{7}$	859	1044	14.7	102
$Ni_{47.7}Co_{17.5}Cr_{10}Fe_{9.3}Al_{7.5}Ti_{7}Mo_{1}$	899	1064	14.3	
CrFeCoNi-2C	321	700	38.1	103
CrFeCoNi-2.5C	352	755	41.1	
CrFeCoNi-3C	339	773	37.4	
$Fe_{49.5}Mn_{30}Co_{10}Cr_{10}C_{0.5}$	134	486	39.3	104
$Ni_{45}(FeCoCr)_{40}(AlTi)_{15}$	811	1009	11	105
$Al_{0.3}CoCrFeNi$	119	295	59	106
$Al_{0.3}CoCrFeNiMn_{0.1}$	149	336	49	
$Al_{0.3}CoCrFeNiMn_{0.3}$	158	371	46	
$Fe_{29}Ni_{29}Co_{28}Cu_{7}Ti_{7}$	917	1060	1.8	107
$Fe_{29}Ni_{29}Co_{28}Cu_{7}Ti_{7}$	411	681	52	
$(FeNiCrMn)_{95}Co_{5}$	274	486	56.3	108
$(FeNiCrMn)_{90}Co_{10}$	293	535	60.1	
$(FeNiCrMn)_{80}Co_{20}$	260	514	64.4	
$CoCrFeNiZr_{0.1}$	280	592	11	109
$CoCrFeNiZr_{0.2}$	500	642	3.7	
$CoCrFeNiZr_{0.3}$	554	752	2.1	
$CoCrFeNiZr_{0.4}$	668	668	1.3	
$Co_{22.35}Cr_{19.67}Fe_{22.85}Ni_{22.44}Mn_{8.62}Al_{3.37}C_{0.69}$	210	455	74	110
FeCoNi	191	495	46.6	111
$FeCoNiMn_{0.25}Al_{0.25}$	300	649	42.6	
$FeCoNiMn_{0.5}Al_{0.5}$	355	973	26.8	
CrCoNi	360	870	38	112
$Fe_{40.4}Ni_{11.3}Mn_{34.8}Al_{7.5}Cr_{6}$	175	387	39.9	113
$Fe_{40.4}Ni_{11.3}Mn_{34.8}Al_{7.5}Cr_{6}C_{1.1}$	373	749	49.6	
$Fe_{0.4}Cr_{0.4}NiCu$	332	608	19.2	114
$Fe_{0.4}Cr_{0.4}NiMn_{0.2}Cu$	355	740	22	
$Fe_{0.4}Cr_{0.4}NiMn_{0.4}Cu$	401	766	21.2	
$Fe_{0.4}Cr_{0.4}NiMn_{0.6}Cu$	406	763	20.5	
$Fe_{0.4}Cr_{0.4}NiMn_{0.8}Cu$	429	831	20.5	
$Fe_{0.4}Cr_{0.4}NiMnCu$	439	885	23.5	
$Fe_{0.4}Cr_{0.4}NiMn_{1.2}Cu$	385	769	19.3	
$Fe_{0.4}Cr_{0.4}NiMn_{1.4}Cu$	387	622	7.3	
CoCrFeMn	338	795	4.3	115
$(CoCrFeMn)_{90}Ni_{10}$	246	450	18.2	
CoCrFeMnNi	245	530	49.1	
$CoCrFeAlNi_{1.5}$	795	1150	3.1	
$CoCrFeAlNi_{2}$	680	1130	21.2	
$CoCrFeAlNi_{2.5}$	470	900	25.3	
$CoCrFeAlNi_{3}$	387	810	31.2	

$CoCrFeAlNi_4$	312	660	39	
$Fe_{40.4}Ni_{11.3}Mn_{34.8}Al_{7.5}Cr_6$	159	388	40.8	116
$Fe_{40.4}Ni_{11.3}Mn_{34.8}Al_{7.5}Cr_6C_{0.07}$	171	436	51.6	
$Fe_{40.4}Ni_{11.3}Mn_{34.8}Al_{7.5}Cr_6C_{0.16}$	181	466	52.2	
$Fe_{40.4}Ni_{11.3}Mn_{34.8}Al_{7.5}Cr_6C_{0.3}$	208	502	48.2	
$Fe_{40.4}Ni_{11.3}Mn_{34.8}Al_{7.5}Cr_6C_{0.55}$	274	594	52.3	
$Fe_{40.4}Ni_{11.3}Mn_{34.8}Al_{7.5}Cr_6C_{1.1}$	355	752	49.5	
$(FeCoNiCr)_{94}Ti_2Al_4$	159	503	64.4	117
$Fe_{36}Ni_{18}Mn_{33}Al_{13}$	270	578	22.8	118
$Fe_{36}Ni_{18}Mn_{33}Al_{13}Ti_2$	532	876	13.9	
$Fe_{36}Ni_{18}Mn_{33}Al_{13}Ti_4$	953	1145	2.3	
$Al_{10.5}Cr_{24.75}Fe_{20}Co_{20}Ni_{24.75}$	370	790	25	119
$Al_{13}Cr_{23.5}Fe_{20}Co_{20}Ni_{23.5}$	760	1010	6	
CoCrFeNi	147	413	48	120
$CoCrFeNiNb_{0.103}$	317	622	19.2	
$CoCrFeNiNb_{0.155}$	321	744	23.3	
$CoCrFeNiNb_{0.206}$	402	807	8.6	
$CoCrFeNiNb_{0.309}$	478	879	3.5	
$CoCrFeNiNb_{0.412}$	637	1004	1.3	
$Al_{0.25}CoCrFe_{1.25}Ni_{1.25}$	92	420	45.3	121
$Al_{0.5}CrCuFeNi_2$	402	570	17.5	122
$Al_{0.5}CrCuFeNi_2$	363	500	16.1	123
$Fe_{37}Mn_{45}Co_9Cr_9$	90	373	44.7	124
$Fe_{40}Mn_{27}Ni_{26}Co_5Cr_2$	90	373	47	
FeCoNiCrMn	198	500	53.1	125
$(FeCoNiCrMn)_{96}Al_4$	210	500	49.8	
$(FeCoNiCrMn)_{93}Al_7$	236	530	43.5	
$(FeCoNiCrMn)_{92}Al_8$	276	647	35.5	
$(FeCoNiCrMn)_{91}Al_9$	288	728	30.7	
$(FeCoNiCrMn)_{90}Al_{10}$	531	994	18.7	
$(FeCoNiCrMn)_{89}Al_{11}$	902	1174	7.8	
FeMnNiCuCoSn	163	474	13.5	126
$FeMnNiCuCoSn_{0.03}$	163	467	17.2	
$FeMnNiCuCoSn_{0.05}$	163	475	10.9	
$FeMnNiCuCoSn_{0.08}$	163	423	7	
$FeMnNiCuCoSn_{0.1}$	163	471	6	
$FeMnNiCuCoSn_{0.2}$	163	368	2.4	
CoCrFeMnNi	213	588	39.5	127
CoCrFeNi	309	667	42	
$FeCoNiAl_{0.2}Si_{0.2}$	280	636	41.6	128
$Al_8Co_{17}Cr_{17}Cu_8Fe_{17}Ni_{33}$	357	459	9	129
AlCrCuNiFeCo	790	790	0.2	130
CoCrFeNiCu	350	360	13	131

$CoCrCuFeNiAl_{0.5}$	360	707	19.1	132
$Al_{0.3}CoCrFeNi$	170	342	58.3	133
CoCrFeNi	160	450	64	134
CoCrFeNiCu	320	500	60	
$Co_{0.4}Fe_{0.3}Ni_{0.3}$	103	202	71.3	135
$(Co_{0.4}Fe_{0.3}Ni_{0.3})_{97.5}(Al_{0.4}Mn_{0.6})_{2.5}$	121	304	44.2	
$(Co_{0.4}Fe_{0.3}Ni_{0.3})_{95}(Al_{0.4}Mn_{0.6})_{5}$	127	329	52.1	
$(Co_{0.4}Fe_{0.3}Ni_{0.3})_{92.5}(Al_{0.4}Mn_{0.6})_{7.5}$	142	383	43.1	
$(Co_{0.4}Fe_{0.3}Ni_{0.3})_{90}(Al_{0.4}Mn_{0.6})_{10}$	143	406	45.2	
$Ni_{50}Co_{25}Cr_{9.5}Al_{9}Ti_{5}Ta_{1.5}$	1000	1250	12	136
**Eutectic CCAs**				
$Al_{20.45}Co_{10}Cr_{10}Ni_{59.55}$	710	718	1.8	137
$Al_{19.3}Co_{15}Cr_{15}Ni_{50.7}$	699	1127	10.3	
$Al_{17.5}Co_{20}Cr_{20}Ni_{52.5}$	740	1272	14.4	
$Al_{16.3}Co_{25}Cr_{25}Ni_{33.7}$	654	1109	13.6	
$AlCrFe_{1.5}Ni_{2.6}$	484	923	18.1	138
$Ni_{30}Co_{30}Cr_{10}Fe_{10}Al_{18}Mo_{2}$	628	1096	15	139
$Co_{30}Cr_{10}Fe_{10}Al_{18}Ni_{32}$	724	1130	16.1	140
$Al_{18}Co_{24}Cr_{20}Ni_{38}$	543	1005	8.1	141
$CoCrNi_{2}(V_{2}B)_{0.43}$	588	1028	1.2	142
$CoCrFeNi_{2}(V_{2}B)_{0.5}$	567	1137	2.1	
$CoCrNi_{2}(V_{3}B_{2}Si)_{0.2}$	567	106	1.8	
$CoCrFeNi_{2}(V_{6}B_{3}Si)_{0.149}$	531	1139	3.3	
$Al_{0.9}CoFeNi_{2}$	559	1005	6.2	143
$Al_{19.25}Co_{18.86}Fe_{18.36}Ni_{43.53}$	486	956	10	144
$CrFeNi_{2.2}Al_{0.8}$	479	956	12.7	145
$Ni_{30}Co_{30}Cr_{10}Fe_{10}Al_{18}W_{2}$	620	1267	20.3	146
$AlCoCrFeNi_{2}$	488	1088	12.2	147
$Fe_{20}Co_{20}Ni_{41}Al_{19}$	577	1103	18.7	148
$AlCoCrFeNi_{2.1}$	545	1052	17.6	149
$AlCoCrFeNi_{2.1}$	500	1100	18	150
$AlCoCrFeNi_{2.1}$	78	944	25.6	151
$Fe_{30}Ni_{20}Mn_{35}Al_{15}$	740	/	8	152
$Al_{17}Co_{14.3}Cr_{14.3}Fe_{14.3}Ni_{40.1}$	479	1067	14	153
$Al_{17}Co_{28.6}Cr_{14.3}Fe_{14.3}Ni_{25.8}$	473	1001	14.8	
$Al_{17}Co_{14.3}Cr_{14.3}Fe_{28.6}Ni_{25.8}$	731	1145	10.3	
$AlCoCrFeNi_{2.1}$	610	950	8	154
$Ti_{30}Ni_{30}Fe_{10}Hf_{10}Nb_{20}$	700	980	1.5	155
$AlCrFeNi_{3}$	580	1200	11.8	156
$Al_{0.9}CoCrNi_{2.1}$	490	1000	6.2	157
$Ni_{49}Fe_{20}Al_{17}V_{6}Co_{4}Cr_{4}$	700	1200	15.5	158
$Al_{18}Co_{30}Cr_{11}Fe_{11}Ni_{30}$	520	960	16	159
$Al_{18}Co_{13}Cr_{10}Fe_{14}Ni_{45}$	623	1047	10.8	160

$(Al_{18}Co_{13}Cr_{10}Fe_{14}Ni_{45})_{99.5}Ti_{0.5}$	690	1065	9.2	
$Al_{19}Co_{23}Fe_{21}Ni_{37}$	510	970	10	161
$(AlCoFe)_{55}(Ni)_{45}$	580	1030	17.5	162
$Al_{1.2}CoCrFeNi_3$	512	961	15.0	163
$Al_{1.25}CoCrFeNi_3$	535	1060	17.2	
$Cr_{41}Ni_{39}Co_{10}V_{10}$	591	1076	10	164
$Cr_{37}Ni_{43}Fe_{10}V_{10}$	537	940	10	
$Cr_{39}Ni_{37}Co_8Fe_8V_8$	434	843	10	
$Cr_{47}Ni_{33}Co_{10}Fe_{10}$	372	727	14	
$Ni_{44}Co_{10}Cr_{12}Fe_{15}Al_{17}W_2$	805	1240	17.7	165
$Co_{17.33}Cr_{17.33}Ni_{47.33}Al_{18}$	700	1180	9.2	166
$AlCoCr_{0.5}FeNi_{2.1}$	480	1030	12.8	167
$Al_{0.8}CrFeNi_{2.2}$	654	1050	10.8	168
$Al_{0.82}Cr_{0.8}Fe_{1.2}Ni_{2.18}$	575	1012	12.6	
$Al_{0.83}Cr_{0.6}Fe_{1.4}Ni_{2.17}$	549	979	12.1	
$Al_{0.85}Cr_{0.4}Fe_{1.6}Ni_{2.15}$	490	951	15.2	
$Co_{30}Cr_{10}Fe_{10}Al_{18}Ni_{30}Mo_2$	490	1100	13.8	169
$Ni_{52}Co_{10}Cr_{20}Al_{18}$	620	1180	16	170
$Al_{17}Cr_{17}Co_{33}Ni_{33}$	490	1028	19.1	171
$Al_{20}Co_{36}Cr_4Fe_4Ni_{36}$	483	1000	9.6	172
$Ni_{49}Fe_{20}Al_{17}Cr_8V_6$	795	1232	16.3	173
$Ni_{49}Fe_{20}Al_{17}Cr_8V_6$	790	1230	16.5	174
$CoCrFeNi(TiC)_{0.2}$	330	760	15.2	175
**Near Eutectic CCAs**		♦		
$Al_{0.5}CoCrFeNi_{2.1}$	257	670	56	176
$Al_{0.7}CoCrFeNi_{2.1}$	412	904	36	
$Al_{0.9}CoCrFeNi_{2.1}$	687	1028	13.9	
$Al_{1.1}CoCrFeNi_{2.1}$	812	1371	14.1	
$AlCoCrFeNi_{2.4}$	619	1078	13	177
$Ni_{1.71}FeCoCrAl$	1050	1550	17.5	178
$Co_{30}Cr_{10}Fe_{10}Al_{18}Ni_{31}Mo_1$	592	1150	17.4	169
$Co_{30}Cr_{10}Fe_{10}Al_{18}Ni_{30}Mo_2$	596	1250	14.6	
$Co_{30}Cr_{10}Fe_{10}Al_{18}Ni_{29}Mo_3$	588	1200	13.7	
$Al_{22}Co_{24}Cr_{20}Ni_{34}$	753	895	2.2	141
$Al_{20}Co_{24}Cr_{20}Ni_{36}$	616	1060	5.4	
$Al_{16}Co_{24}Cr_{20}Ni_{40}$	461	1028	19.3	
$Al_{14}Co_{24}Cr_{20}Ni_{42}$	423	873	31.2	
$CrFeNi_{2.5}Mo$	781	1015	3.4	179
$Al_{0.2}CrFeNi_{2.5}Mo_{0.8}$	773	933	5.6	
$Al_{0.5}CrFeNi_{2.5}Mo_{0.5}$	596	826	12.6	
$Al_{0.8}CrFeNi_{2.5}Mo_{0.4}$	609	951	15	
$AlCrFeNi_{2.5}$	502	1090	16.8	
$CrFe_2Ni_2Mo_{0.3}Nb_{0.25}$	665	869	16.7	54

$CrFe_2Ni_2Mo_{0.3}Nb_{0.5}$	580	580	2.5	
$CrFe_2Ni_2Mo_{0.3}Nb_{0.75}$	359	359	1.6	
$Ni_{40}Co_{20}Cr_{10}Fe_{10}Al_{18}Mo_2$	640	1264	18	
$Ni_{50}Co_{10}Cr_{10}Fe_{10}Al_{18}Mo_2$	791	1252	14	139
$Ni_{60}Cr_{10}Fe_{10}Al_{18}Mo_2$	832	1358	14.5	
$AlCoCrFeNi_{2.1}Ti_{0.1}$	584	1119	8.2	
$AlCoCrFeNi_{2.1}Ti_{0.15}$	603	1253	12.9	180
$AlCoCrFeNi_{2.1}Ti_{0.2}$	538	1217	6.9	
$AlCoCrFeNi_3$	368	675	24.8	181
$AlCoCrNi_2$	553	1200	8.9	
$Al_{0.8}CoCrNi_{2.2}$	578	969	17.3	157
$Al_{0.7}CoCrNi_{2.3}$	518	853	26.2	
$AlCo_{0.4}CrFeNi_{2.7}$	540	941	8.5	182
$CrFeNi_{2.3}Al_{0.7}$	461	835	30	
$CrFeNi_{2.1}Al_{0.9}$	610	1173	9.1	145
$CrFeNi_2Al$	774	1357	6.4	
$AlCoCrFeNi_{2.0}$	545	1076	16.6	149
$AlCoCrFeNi_{2.2}$	545	1123	20.3	
**CCAs with spinodal decomposition**				
$Al_{0.4}Co_{0.5}FeNiV_{0.6}$	703	1128	27.7	183
$AlCrFe_2Ni_2$	986	11.4	11.8	184
$AlCoCr_2FeNi_2$	672	1330	7.2	185
$CrFeNiAl_{0.28}Si_{0.12}$	656	1130	8.1	186
$CrFeNiAl_{0.27}Si_{0.11}Mo_{0.02}$	552	1011	15.5	187
$Al_{0.4}Co_{0.5}V_{0.6}FeNi$	583	1113	27.8	188
$Fe_{37}Ni_{36}Al_{17}Cr_{10}$	511	1100	14.5	
$Fe_{36}Ni_{36}Al_{17}Cr_{10}Mo_1$	571	1178	14.9	189
$Fe_{35}Ni_{36}$Al17$Cr_{10}Mo_2$	863	1285	16.1	
$Fe_{34}Ni_{36}$Al17$Cr_{10}Mo_3$	840	1260	16	
$Al_{0.6}CoCrFeNi$	360	939	32.6	190
$CoCr_3Fe_5Ni$	322	643	55	
$Al_{0.1}CoCr_3Fe_5Ni$	412	781	38.1	191
$Al_{0.2}CoCr_3Fe_5Ni$	568	854	28	
$Al_{0.3}CoCr_3Fe_5Ni$	831	1019	16.1	
$Al_{0.1}CrFe_2Ni_2$	202	493	53.2	
$Al_{0.3}CrFe_2Ni_2$	209	509	50.7	
$Al_{0.5}CrFe_2Ni_2$	315	705	47.4	
$Al_{0.6}CrFe_2Ni_2$	376	737	40.5	192
$Al_{0.7}CrFe_2Ni_2$	610	869	27.1	
$Al_{0.8}CrFe_2Ni_2$	850	1083	20	
$Al_{0.9}CrFe_2Ni_2$	1100	1282	12.5	
$AlCrFe_2Ni_2$	777	1033	19	
$Al_{0.6}CrFe_2Ni_2Mo_{0.2}$	341	744	52	193

$Al_{0.7}CrFe_2Ni_2Mo_{0.2}$	398	785	25	
$Al_{0.8}CrFe_2Ni_2Mo_{0.2}$	622	1059	9	
$Al_{0.9}CrFe_2Ni_2Mo_{0.2}$	657	1067	8	
$AlCoCr_{1.6}FeNi_2$	475	1078	16	147
$AlCoCr_{1.8}FeNi_2$	608	1227	11.2	
$AlCoCr_2FeNi_2$	843	1372	7.3	
$AlCoCr_{2.2}FeNi_2$	830	1347	4.7	
$Al_{0.6}CoCrFeNi$	412	932	21	194
$Al_{13}Co_{21}Cr_{21}Fe_{21}Ni_{21}Ti_3$	450	908	16	
$Al_{16}Co_{21}Cr_{21}Fe_{21}Ni_{21}$	1207	1398	1	
$Al_{13}Co_{21.69}Cr_{21.69}Fe_{21.69}Ni_{21.69}C_{0.25}$	1098	1415	5	
$Al_{13}Co_{20.94}Cr_{20.94}Fe_{20.94}Ni_{20.94}Ti3_{C0.25}$	960	1344	5	
$AlCrFe_2Ni_2$	780	1228	17	195
$Al_{0.7}CoCrFe_2Ni$	866	1223	7.9	196
$(AlCrFe2)_{65}Ni_{35}$	810	1320	11	197
$Al_{7.7}Cr_{25}Fe_{25}Ni_{42.3}$, wt.%	850	1330	18	198
$FeNi_{0.9}Cr_{0.5}Al_{0.4}$	670	1196	21	199
**Dual phase or multiphase CCAs**				
$Ni_2CrFeAl_{0.3}Ti_{0.3}$	898	1240	11.2	29
$FeMn_{0.7}Ni_{0.6}Cr_{0.4}Al_{0.3}$	645	1061	19.5	200
$FeMnNiCrCu_{0.5}$	257	499	34.4	201
$Al_{0.25}FeMnNiCrCu_{0.5}$	573	927	20.3	
$Al_{0.5}FeMnNiCrCu_{0.5}$	985	1212	6.5	
$AlFeMnNiCrCu_{0.5}$	621	/	2.5	
$Fe_{49.85}Cr_{10.03}Mn_{10.03}Co_{10.03}Ni_{10.03}Al_{10.03}$	300	637	30	202
$Fe_{23}Co_{24}Ni_{24}Cr_{21}Al_8$	426	780	48.3	203
$Al_{0.25}FeCoNiV$	510	1045	30	204
$Fe_{36}Mn_{36}Ni_9Cr_9Al_{10}$	705	842	25	205
$(Fe_{36}Mn_{36}Ni_9Cr_9Al_{10})_{98.5}C_{1.5}$	426	755	49	
$(Fe_{36}Mn_{36}Ni_9Cr_9Al_{10})_{97.5}C_{2.5}$	387	741	57	
$(Fe_{36}Mn_{36}Ni_9Cr_9Al_{10})_{96.5}C_{3.5}$	329	694	50	
$(Fe_{36}Mn_{36}Ni_9Cr_9Al_{10})_{95}C_5$	453	757	33	
$(Fe_{36}Mn_{36}Ni_9Cr_9Al_{10})_{94}C_6$	528	707	15	
$Fe_{40.2}Ni_{11.3}Mn_{30}Al_{7.5}Cr_{11}$	568	898	21.4	206
$Al_2(NiCoFeCr)_{14}$	567	929	15.5	3
$Al_2(Ni_4Co_4Fe_3Cr_3)_{14}$	335	730	33	
$Al_2(NiCoFe_2Cr)_{14}$	927	1245	8.1	
$Cr_{30}Fe_{30}Ni_{30}Al_5Ti_5$	1175	1621	12	207

Table S4. A summary of tensile properties in Fig. S1, including yield strength ($\sigma_{0.2}$), ultimate tensile strength (σ_{uts}), uniform elongation (ε_u) and corresponding references of directly synthesized as-cast eutectic CCAs.

Composition	$\sigma_{0.2}$ (MPa)	σ_{uts} (MPa)	ε_u (%)	Ref
$Al_{20.45}Co_{10}Cr_{10}Ni_{59.55}$	710	718	1.8	137
$Al_{19.3}Co_{15}Cr_{15}Ni_{50.7}$	699	1127	10.3	
$Al_{17.5}Co_{20}Cr_{20}Ni_{52.5}$	740	1272	14.4	
$Al_{16.3}Co_{25}Cr_{25}Ni_{33.7}$	654	1109	13.6	
$AlCrFe_{1.5}Ni_{2.6}$	484	923	18.1	138
$Ni_{30}Co_{30}Cr_{10}Fe_{10}Al_{18}Mo_2$	628	1096	15	139
$Co_{30}Cr_{10}Fe_{10}Al_{18}Ni_{32}$	724	1130	16.1	140
$Al_{18}Co_{24}Cr_{20}Ni_{38}$	543	1005	8.1	141
$CoCrNi_2(V_2B)_{0.43}$	588	1028	1.2	142
$CoCrFeNi_2(V_2B)_{0.5}$	567	1137	2.1	
$CoCrNi_2(V_3B_2Si)_{0.2}$	567	106	1.8	
$CoCrFeNi_2(V_6B_3Si)_{0.149}$	531	1139	3.3	
$Al_{0.9}CoFeNi_2$	559	1005	6.2	143
$Al_{19.25}Co_{18.86}Fe_{18.36}Ni_{43.53}$	486	956	10	144
$CrFeNi_{2.2}Al_{0.8}$	479	956	12.7	145
$Ni_{30}Co_{30}Cr_{10}Fe_{10}Al_{18}W_2$	620	1267	20.3	146
$AlCoCrFeNi_2$	488	1088	12.2	147
$Fe_{20}Co_{20}Ni_{41}Al_{19}$	577	1103	18.7	148
$AlCoCrFeNi_{2.1}$	545	1052	17.6	149
$AlCoCrFeNi_{2.1}$	500	1100	18	150
$AlCoCrFeNi_{2.1}$	78	944	25.6	151
$Fe_{30}Ni_{20}Mn_{35}Al_{15}$	740	/	8	152
$Al_{17}Co_{14.3}Cr_{14.3}Fe_{14.3}Ni_{40.1}$	479	1067	14	153
$Al_{17}Co_{28.6}Cr_{14.3}Fe_{14.3}Ni_{25.8}$	473	1001	14.8	
$Al_{17}Co_{14.3}Cr_{14.3}Fe_{28.6}Ni_{25.8}$	731	1145	10.3	
$AlCoCrFeNi_{2.1}$	610	950	8	154
$Ti_{30}Ni_{30}Fe_{10}Hf_{10}Nb_{20}$	700	980	1.5	155
$AlCrFeNi_3$	580	1200	11.8	156
$Al_{0.9}CoCrNi_{2.1}$	490	1000	6.2	157
$Ni_{49}Fe_{20}Al_{17}V_6Co_4Cr_4$	700	1200	15.5	158
$Al_{18}Co_{30}Cr_{11}Fe_{11}Ni_{30}$	520	960	16	159
$Al_{18}Co_{13}Cr_{10}Fe_{14}Ni_{45}$	623	1047	10.8	160
$(Al_{18}Co_{13}Cr_{10}Fe_{14}Ni_{45})_{99.5}Ti_{0.5}$	690	1065	9.2	
$Al_{19}Co_{23}Fe_{21}Ni_{37}$	510	970	10	161
$(AlCoFe)_{55}(Ni)_{45}$	580	1030	17.5	162
$Al_{1.2}CoCrFeNi_3$	512	961	15.0	163
$Al_{1.25}CoCrFeNi_3$	535	1060	17.2	
$Cr_{41}Ni_{39}Co_{10}V_{10}$	591	1076	10	164

$Cr_{37}Ni_{43}Fe_{10}V_{10}$	537	940	10	
$Cr_{39}Ni_{37}Co_8Fe_8V_8$	434	843	10	
$Cr_{47}Ni_{33}Co_{10}Fe_{10}$	372	727	14	
$Ni_{44}Co_{10}Cr_{12}Fe_{15}Al_{17}W_2$	805	1240	17.7	165
$Co_{17.33}Cr_{17.33}Ni_{47.33}Al_{18}$	700	1180	9.2	166
$AlCoCr_{0.5}FeNi_{2.1}$	480	1030	12.8	167
$Al_{0.8}CrFeNi_{2.2}$	654	1050	10.8	168
$Al_{0.82}Cr_{0.8}Fe_{1.2}Ni_{2.18}$	575	1012	12.6	
$Al_{0.83}Cr_{0.6}Fe_{1.4}Ni_{2.17}$	549	979	12.1	
$Al_{0.85}Cr_{0.4}Fe_{1.6}Ni_{2.15}$	490	951	15.2	
$Co_{30}Cr_{10}Fe_{10}Al_{18}Ni_{30}Mo_2$	490	1100	13.8	169
$Ni_{52}Co_{10}Cr_{20}Al_{18}$	620	1180	16	170
$Al_{17}Cr_{17}Co_{33}Ni_{33}$	490	1028	19.1	171
$Al_{20}Co_{36}Cr_4Fe_4Ni_{36}$	483	1000	9.6	172
$Ni_{49}Fe_{20}Al_{17}Cr_8V_6$	795	1232	16.3	173
$Ni_{49}Fe_{20}Al_{17}Cr_8V_6$	790	1230	16.5	174
$CoCrFeNi(TiC)_{0.2}$	330	760	15.2	175

References

44. Trimby P. *et al.*, Advanced classification of microstructures in EBSD datasets using AZtecCrystal. *Microsc. Micronal.* **26**, 112-113 (2020).
45. Zhang, D.D. *et al.*, A strong and ductile NiCoCr-based medium-entropy alloy strengthened by coherent nanoparticles with superb thermal-stability. *J. Mater. Sci. Technol.* **132**, 201-212 (2023).
46. Zhao, Y. L. et al., Heterogeneous precipitation behavior and stacking-fault-mediated deformation in a CoCrNi-based medium-entropy alloy. *Acta Mater.* **138**, 112483 (2017).
47. Zhao, Y. L. et al., Exceptional nanostructure stability and its origins in the CoCrNi-based precipitation-strengthened medium-entropy alloy. *Mater. Res. Lett.* **7**, 152-158 (2019).
48. Chen, Y. A. et al., Dual precipitates and heterogeneous fine-grain structure induced strength-ductility synergy in a CoCrNi-based medium-entropy alloy. *Mater. Sci. Eng: A* **867**, 144504 (2023).
49. Liu, S. Y. et al., Superior hydrogen embrittlement resistance of CoCrNi-based medium-entropy alloy via coherent precipitation and grain boundary strengthening. *Corros. Sci.* **240**, 112483 (2024).
50. Zhang, D. D. et al. Achieving excellent strength-ductility synergy in twinned NiCoCr medium-entropy alloy via Al/Ta co-doping. *J. Mater. Sci. Technol.* **87**, 184-195 (2021).
51. An, N. et al., High temperature strengthening via nanoscale precipitation in wrought CoCrNi-based medium-entropy alloys. *Mater. Sci. Eng. A* **798**, 140213 (2020).
52. Yang, T. et al., Nanoparticles-strengthened high-entropy alloys for cryogenic applications showing an exceptional strength-ductility synergy. Scr. Mater. 164, 30-35 (2019)
53. Fang, J. Y. C., Liu, W. H., Luan, J. H. & Biao, J. Z., Phase stability and precipitation in L12-strengthened CoCrNi medium-entropy alloys at intermediate temperatures. *J. Phase Equilib. Diff.* **42**, 781-793 (2021).
54. Yang, L. et al., Simultaneous enhancement of strength and ductility via microband formation and nanotwinning in an L12-strengthened alloy. *Fundamental Res.* **4**, 147-157 (2022).

55. Zhang, L. et al., Enhancing the strength-ductility trade-off in a NiCoCr-based medium-entropy alloy with the synergetic effect of ultra fine precipitates, stacking faults, dislocation locks and twins. *Scr. Mater.* **211**, 114497 (2022).
56. Zhang, D. D. et al., Superior strength-ductility synergy and strain hardenability of Al/Ta co-doped NiCoCr twinned medium entropy alloy for cryogenic applications. *Acta Mater.* **220**, 117288 (2021).
57. Wu, Q. et al., Effects of Ta microalloying on the microstructure and mechanical properties of L12-strengthened CoCrFeNi-AlTi high-entropy alloys. *Mater. Sci. Eng. A* **875**, 145048 (2023)
58. Tong, Y. et al., Outstanding tensile properties of a precipitation-strengthened FeCoNiCrTi0.2 high-entropy alloy at room and cryogenic temperatures. *Acta Mater.* **165**, 228-240 (2019).
59. Huang, X. et al., Enhancing strength-ductility synergy in a casting non-equiatomic NiCoCr-based high-entropy alloy by Al and Ti combination addition. *Scr. Mater.* **200**, 113898 (2021).
60. GB/T 2975-2018, Spheroidal graphite iron castings, Standards Press of China (2019) (in Chinese).
61. ISO 1083:2018, Spheroidal graphite cast irons, International Organization for Standardization (2019).
62. GSO EN 1563:2024, Spheroidal graphite cast irons, European Committee for Standardization (2024).
63. ASTM A351, Standard specification for castings, austenitic, for Pressure-Containing Parts, (2025).
64. GB/T 2100-2017 Corrosion-resistanst steel castings for general applications, Standards Press of China (2019) (in Chinese).
65. Wu, Z. et al., Temperature dependence of the mechanical properties of equiatomic solid solution alloys with face-centered cubic crystal structures. *Acta Mater.* **81**, 428-441 (2014).
66. Varvenne, C., Luque, A. & Curtin, W.A., Theory of strengthening in fcc high entropy alloys. *Acta Mater.* **118**, 164-176 (2016).
67. Yin, B., Maresca, F. & Curtin, W. A., Vanadium is an optimal element for strengthening in both fcc and bcc high-entropy alloys. *Acta Mater.* **188**, 486-491 (2020).
68. Liu, G. et al., Nanostructured high-strength molybdenum alloys with unprecedented tensile ductility. *Nat. Mater.* **12,** 344-350 (2013).

References of Tables S3 and S4

1. D. Gu, et al., Designing (TiZrHf) $_{100-x}Cu_x$ high entropy alloys with abnormal as-cast divorced eutectoid microstructure and balanced mechanical properties. *Mater. Sci. Eng. A* **879,** 145217 (2023).
2. L. Rosenkranz, et al., Tensile behavior of hexagonal rare-earth-based low, medium, and high entropy alloys: Strengthening effect of configurational entropy. *Intermetallics* **155,** 107835 (2023).
3. Y. Ma, et al., Controlled formation of coherent cuboidal nanoprecipitates in body-centered cubic high-entropy alloys based on Al_2 $(Ni,Co,Fe,Cr)_{14}$ compositions. *Acta Mater.* **147:** 213-225 (2018).
4. N. Yurchenko, et al., Effect of B2 ordering on the tensile mechanical properties of refractory $Al_xNb_{40}Ti_{40}V_{20-x}$ medium-entropy alloys. *J. Alloy. Compd.* **937,** 168465 (2023).
5. R. Huang, et al., A novel AlMoNbHfTi refractory high-entropy alloy with superior ductility. *J. Alloy. Compd.* **940,** 168821 (2023).
6. W. Huang, et al., Excellent room-temperature tensile ductility in as-cast $Ti_{37}V_{15}Nb_{22}Hf_{23}W_3$ refractory high entropy alloys. *Intermetallics* **151,** 107735 (2022).
7. Z. Q. Xu, et al., Designing TiVNbTaSi refractory high-entropy alloys with ambient tensile ductility. *Scr. Mater.* **206,** 114230 (2022).
8. W. Lai, et al. Design of BCC refractory multi-principal element alloys with superior mechanical properties. *Mater. Res. Lett.* **10,** 133-140 (2022).

9. X. Yan, P. K. Liaw, & Y. Zhang., Ultrastrong and ductile BCC high-entropy alloys with low-density via dislocation regulation and nanoprecipitates. *J. Mater. Sci. Technol.* **110,** 109-116 (2022).
10. R. Wang, et al., Achieving high strength and ductility in nitrogen-doped refractory high-entropy alloys. *Mater. Des.* **213,** 110356 (2022).
11. X. Wen, et al., Effects of Nb on deformation-induced transformation and mechanical properties of $HfNb_xTa_{0.2}TiZr$ high entropy alloys. *Mater. Sci. Eng. A* **805,** 140798 (2021).
12. D. Li, et al., An as-cast Ti-V-Cr-Al light-weight medium entropy alloy with outstanding tensile properties. *J. Alloy. Compd.* **877,** 160199 (2021).
13. Y. C. Liao, et al., Effect of Al concentration on the microstructural and mechanical properties of lightweight $Ti_{60}Al_x(VCrNb)_{40-x}$ medium-entropy alloys. *Intermetallics* **135,** 107213 (2021).
14. L. Mustafi, et al., Microstructure, tensile properties and deformation behaviour of a promising bio-applicable new $Ti_{35}Zr_{15}Nb_{25}Ta_{25}$ medium entropy alloy (MEA). *Mater. Sci. Eng. A* **824,** 141805 (2021).
15. Z. Han, et al., Novel BCC VNbTa refractory multi-element alloys with superior tensile properties. *Mater. Sci. Eng. A* **825,** 141908 (2021).
16. Y. Chen, et al., A single-phase $V_{0.5}Nb_{0.5}ZrTi$ refractory high-entropy alloy with outstanding tensile properties. *Mater. Sci. Eng. A* **792,** 139774 (2020).
17. S. Wei, et al., Natural-mixing guided design of refractory high-entropy alloys with as-cast tensile ductility. *Nat. Mater*. **19,** 1175-1181 (2020).
18. X. Yan, & Y. Zhang., A body-centered cubic $Zr_{50}Ti_{35}Nb_{15}$ medium-entropy alloy with unique properties. *Scr. Mater.* **178,** 329-333 (2020).
19. Q. He, et al., Effect of elemental combination on microstructure and mechanical properties of quaternary refractory medium entropy alloys. *Mater. Tran*. **61,** 577-586 (2020).
20. T. Huang, et al., Effect of Ti content on microstructure and properties of Ti_xZrVNb refractory high-entropy alloys. *Int. J. Min. Met. Mater*. **27,** 1318-1325 (2020).
21. R. R. Eleti, et al., Exceptionally high strain-hardening and ductility due to transformation induced plasticity effect in Ti-rich high-entropy alloys. *Sci. Rep*. **34,** 13293 (2020).
22. Y. C. Liao, et al., Designing novel lightweight, high-strength and high-plasticity $Ti_x(AlCrNb)_{100-x}$ medium-entropy alloys. *Intermetallics* **117,** 106673 (2020).
23. S. P. Wang, E. Ma, & J. Xu., New ternary equi-atomic refractory medium-entropy alloys with tensile ductility: Hafnium versus titanium into NbTa-based solution. *Intermetallics* **107,** 15-23 (2019).
24. L. Lilensten, et al., Design and tensile properties of a bcc Ti-rich high-entropy alloy with transformation-induced plasticity. *Mater. Res. Lett*. **147,** 110-116 (2017).
25. H. Huang, et al., Phase‐transformation ductilization of brittle high‐entropy alloys via metastability engineering. *Adv. Mater*. **462,** 1701678 (2017).
26. S. Sheikh, et al., Alloy design for intrinsically ductile refractory high-entropy alloys. *J. Appl. Phys*. **120,** 164902 (2016).
27. G. Dirras, et al., Elastic and plastic properties of as-cast equimolar TiHfZrTaNb high-entropy alloy. *Mater. Sci. Eng. A* **654:** 30-38 (2016).
28. L. Mustafi, et al., A new non-equiatomic $Ti_{40}Zr_{25}Nb_{25}Ta_5Al_5$ refractory high entropy alloy for potential biomedical applications. *J. Alloys Compd.* **1026**, 180299 (2025).
29. S. Z. Peng, et al., Hierarchical coherent precipitates lead to high tensile strength in casting BCC-structured $Al_{0.8}CrNiMn_2Fe_{2.5}$ high-entropy alloy. *J. Mater. Res. Technol.* **36**, 903-912 (2025).
30. H. Wang, et al., Microstructural evolution and mechanical behavior of novel $Ti_{1.6}ZrNbAl_x$ lightweight refractory high-entropy alloys containing BCC/B2 phases. *Mater. Sci. Eng. A* **885,** 145661 (2023).

31. H. Wang, et al., Lightweight Ti-Zr-Nb-Al-V refractory high-entropy alloys with superior strength-ductility synergy and corrosion resistance. *Int. J. Refract. Met. Hard Mater.* **116**, 106331 (2023).
32. S. Huang, et al., Enhanced tensile properties of $CrMnFeCoNi_{0.8}$ high entropy alloy with in-situ TiC particles. *Intermetallics* **148,** 107639 (2022).
33. X. X. Liu, et al., Microstructure evolution and mechanical response of Co-free $Ni_2CrFeAl_{0.3}Ti_x$ high-entropy alloys. *J. Alloy. Compd.* **931,** 167523 (2023).
34. T. Liu, et al., Microstructure evolution and strengthening mechanisms in $Ni_{36}Co_{30}Cr_{11}Fe_{11}Al_{12-x}Nb_x$ high entropy alloys. *J. Alloy. Compd.* **946,** 169390 (2023).
35. J. Tian, et al., Effects of Al alloying on microstructure and mechanical properties of VCoNi medium entropy alloy. *Mater. Sci. Eng. A* **811,** 141054 (2021).
36. Q. Hu, et al., Effect of Al on microstructure and mechanical properties of cast CrCoNi medium-entropy alloy. *China Foundry* **15,** 253-262 (2018).
37. J. Wang, et al., Microstructure and properties of CoCrNi medium-entropy alloy produced by gas atomization and spark plasma sintering. *J. Mater. Res*. **34,** 2126-2136 (2019).
38. J. Wu, H. Zhu, & Z. Xie., Strength and ductility synergy of Nb-alloyed $Ni_{0.6}CoFe_{1.4}$ alloys. *Int. J. Min. Met. Mater*. **30,** 707-714 (2023).
39. W. Guo, et al., Mechanical performances and processing-property modeling for $Al_{0.3}CoCrFeNiMn$ high-entropy alloy. *J. Alloy. Compd.* **905,** 163791 (2022).
40. F. Otto, et al., The influences of temperature and microstructure on the tensile properties of a CoCrFeMnNi high-entropy alloy. *Acta Mater.* **61,** 5743-5755 (2013).
41. X. Zhou, et al., Effect of Mn on microstructure and tensile properties of as-cast $Al_{0.5}CoFeNiC_{0.1}$ high-entropy alloy. *Mater. Sci. Eng. A* **873,** 144951 (2023).
42. Q. Shen, et al., Microstructures and mechanical properties of the precipitation strengthened $Al_{0.4}Cr_{0.7}Fe_xNi_2V_{0.2}$ high entropy alloys. *Mater. Sci. Eng. A* **864,** 144606 (2023).
43. M. R. Zamani, et al., Unveiling the strengthening mechanisms of as-cast micro-alloyed CrMnFeCoNi high-entropy alloys. *J. Alloy. Compd.* **957,** 170443 (2023).
44. S. H. Shim, H. Pouraliakbar, & S. I. Hong., Hierarchical structured as-cast $CrFeNiMn_{0.5}Cu_{0.5}$ high entropy alloy with excellent tensile strength/ductility properties. *Scr. Mater*. **210,** 114473 (2022).
45. X. Gu, Y. Zhuang, & P. Jia., Evolution of phase, microstructure and mechanical properties of as-cast $Al_{0.3}$CoCrFeNiTix high entropy alloys. *Mater. Today Commun*. **31,** 103328 (2022).
46. X. Gu, Y. Zhuang, & P. Jia., Evolution of the microstructure and mechanical properties of as-cast $Al_{0.3}CoCrFeNi$ high entropy alloys by adding Si content. *Mater. Sci. Eng. A* **840,** 142983 (2022).
47. R. Fan, et al., Synergistic effect of Nb and Mo alloying on the microstructure and mechanical properties of CoCrFeNi high entropy alloy. *Mater. Sci. Eng. A* **829,** 142153 (2022).
48. X. Gao, et al., Precipitation phase and twins strengthening behaviors of as-cast non-equiatomic CoCrFeNiMo high entropy alloys. *J. Alloy. Compd.* **918,** 165584 (2022).
49. C. Chen, et al., Microstructure, mechanical properties, corrosion resistance and anti-bacterial behavior of novel Co-free high entropy alloys. *J. Alloy. Compd.* **902,** 163714 (2022).
50. X. Gao, et al., Nano-twinning induced high strain hardening behavior in a metastable as-cast $Co_{30}Cr_{30}Fe_{20}Ni_{20}$ high entropy alloy at room temperature. *Mater. Charact*. **194,** 112420 (2022).
51. H. Wang, et al., Mechanical properties and corrosion resistance characterization of a novel $Co_{36}Fe_{36}Cr_{18}Ni_{10}$ high-entropy alloy for bioimplants compared to 316L alloy. *J. Alloy. Compd.* **906,** 163947 (2022).
52. S. Wang, et al., Effects of Al or Mo addition on microstructure and mechanical properties of Fe-rich nonequiatomic FeCrCoMnNi high-entropy alloy. *Metals*. **12,** 191 (2022).

53. T. Liu, et al., Formation of hypoeutectic structure and strengthening mechanism of $Co_4Cr_{1.5}Fe_{1.5}Ni_5$ high entropy alloy alloyed by Al. *J. Alloy. Compd.* **923:** 166372 (2022).
54. W. Wu, et al., A novel hypoeutectic high-entropy alloy $CrFe_2Ni_2Mo_{0.3}Nb_{0.25}$ with high tensile strength and good ductility. *J. Mater. Eng. Perform*. **31,** 7913-7920 (2022).
55. Z. Y. Jia, et al., Heterogeneous precipitation strengthened non-equiatomic NiCoFeAlTi medium entropy alloy with excellent mechanical properties. *Mater. Sci. Eng. A* **834,** 142617 (2022).
56. A. Ayrenk, & I. Kalay. Microstructure and mechanical properties of Al–Co–Cr–Fe–Ni–(Nb–Ti) high entropy alloys. *Philos. Mag*. **102,** 1961-1973 (2022).
57. C. Zhu.et al., Mechanical properties and fracture mechanism of as-cast $MnFeCoCuNi_x$ high-entropy alloys. *T. Nonferr. Metal. Soc. China*. **31,** 222-231 (2021).
58. G. Qin, et al., Experimental and theoretical investigations on the phase stability and mechanical properties of $Cr_7Mn_{25}Co_9Ni_{23}Cu_{36}$ high-entropy alloy. *Acta Mater*. **208,** 116763 (2021).
59. R. K. Nutor, et al., Tunability of the mechanical properties of $(Fe_{50}Mn_{27}Ni_{10}Cr_{13})_{100-x}Mo_x$ high-entropy alloys via secondary phase control. *J Mater. Sci. Technol*. **73,** 210-217 (2021).
60. X. Huang, et al., Enhancing strength−ductility synergy in a casting non-equiatomic NiCoCr-based high-entropy alloy by Al and Ti combination addition. *Scr. Mater.* **200,** 113898 (2021).
61. X. Yan, et al., $Al_{0.3}Cr_xFeCoNi$ high-entropy alloys with high corrosion resistance and good mechanical properties. *J. Alloy. Compd.* **860,** 158436 (2021).
62. F. Yang, et al., Mechanical properties of FeMnCoCr high entropy alloy alloyed with C/Si at low temperatures. *J. Alloy. Compd.* **859,** 157876 (2021).
63. Y. Ji, et al., Microstructure and tensile properties of Co-free $Fe_4CrNi(AlTi)_x$ high-entropy alloys. *Intermetallics* **138,** 107339 (2021).
64. L. Bai, et al., Effects of Al addition on microstructure and mechanical properties of Co-free $(Fe_{40}Mn_{40}Ni_{10}Cr_{10})_{100-x}Al_x$ high-entropy alloys. *J. Alloy. Compd.* **879,** 160342 (2021).
65. S. Huang, et al., Effect of niobium addition upon microstructure and tensile properties of $CrMnFeCoNi_x$ high entropy alloys. *Mater. Sci. Eng. A* **809,** 140959 (2021).
66. J. Li, et al., Microstructures and Mechanical properties of as sast $(Al_{7.5}Co_{21.9}Cr_{10.9}Ti_{5.0}Fe_{21.9}Ni_{32.8})_{100-x}Cu_x$ high-entropy alloys. *Front. Mater*. **8,** 804918 (2021).
67. E. Astafurova, et al., A comparative study of a solid solution hardening in carbon-alloyed $FeMnCrNiCo_{0.95}C_{0.05}$ high-entropy alloy subjected to different thermal–mechanical treatments. *Mater. Lett*. **285,** 129073 (2021).
68. Y. Dong, et al., Excellent strength−ductility synergy in as-cast $Al_{0.6}CoCrFeNi_2Mo_{0.08}V_{0.04}$ high-entropy alloy at room and cryogenic temperatures. *Mater. Lett.***294,** 129778 (2021).
69. W. Jiang, et al., Mechanical properties and deformation mechanisms of a $Ni_2Co_1Fe_1V_{0.5}Mo_{0.2}$ medium-entropy alloy at elevated temperatures. *Acta Mater.* **213,** 116982 (2021).
70. J. Zhang, H. Zhu, & Z. Xie., Effects of Y and Al additions on the microstructure and tensile properties of $CoCr_3Fe_5Ni$ high entropy alloys. *Mater. Lett*. **299,** 130110 (2021).
71. R. K. Nutor, et al., Microstructure and properties of a Co-free $Fe_{50}Mn_{27}Ni_{10}Cr_{13}$ high entropy alloy. *J. Alloy. Compd.* **851,** 156842 (2021).
72. L. Wang, et al., Precipitation and micromechanical behavior of the coherent ordered nanoprecipitation strengthened Al-Cr-Fe-Ni-V high entropy alloy. *Acta Mater*. **216,** 117121 (2021).
73. L. Wang, et al., Microstructure and mechanical properties of $CoCrFeNiW_x$ high entropy alloys reinforced by μ phase particles. *J. Alloy. Compd.* **843,** 155997 (2020).
74. M. Klimova, et al., Effect of nitrogen on mechanical properties of CoCrFeMnNi high entropy alloy at room and cryogenic temperatures. *J. Alloy. Compd.* **849,** 156633 (2020).

75. U. Sunkari, et al., Effect of niobium alloying on the microstructure, phase stability and mechanical properties of $CoCrFeNi_{2.1}Nb_x$ high entropy alloys: Experimentation and thermodynamic modeling. *Mater. Sci. Eng. A* **793,** 139897 (2020).
76. X. Y. Gu, et al., Microstructure and mechanical properties of $CoCrFeMnNiSn_x$ high-entropy alloys. *Met. Mater. Int.* **26,** 292-301 (2020).
77. H. Ma, & C. H. Shek., Effects of Hf on the microstructure and mechanical properties of CoCrFeNi high entropy alloy. *J. Alloy. Compd.* **827,** 154159 (2020).
78. H. Qiu, et al., Effect of Fe content upon the microstructures and mechanical properties of $Fe_xCoNiCu$ high entropy alloys. *Mater. Sci. Eng. A* **769,** 138514 (2020).
79. X. U. Jun, et al., Microstructures, tensile properties and serrated flow of $Al_xCrMnFeCoNi$ high entropy alloys. *T. Nonferr. Metal. Soc. China*. **30,** 746-755 (2020).
80. T. Zhang, et al., Transformation-enhanced strength and ductility in a FeCoCrNiMn dual phase high-entropy alloy. *Mater. Sci. Eng. A* **780,** 139182 (2020).
81. Z. Ma, et al., Enhanced strength and slightly reduced ductility in a high entropy alloy via cold rolling and annealing. *J. Alloy. Compd.* **817,** 152709 (2020).
82. S. Dasari, et al., Engineering multi-scale B2 precipitation in a heterogeneous FCC based microstructure to enhance the mechanical properties of a $Al_{0.5}Co_{1.5}CrFeNi_{1.5}$ high entropy alloy. *J. Alloy. Compd.* **830,** 154707 (2020).
83. F. Yang, et al., Microstructural features and tensile behaviors of a novel FeMnCoCr high entropy alloys. *Mater. Lett.* **275,** 128154 (2020).
84. H. Peng, et al., Ripening of $L1_2$ nanoparticles and their effects on mechanical properties of $Ni_{28}Co_{28}Fe_{21}Cr_{15}Al_4Ti_4$ high-entropy alloys. *Mater. Sci. Eng. A* **772,** 138803 (2020).
85. Z. Li, et al., Improving mechanical properties of an FCC high-entropy alloy by γ' and B2 precipitates strengthening. *Mater. Charact.* **159,** 109989 (2020).
86. L. Zhang, et al., A ductile high entropy alloy strengthened by nano sigma phase. *Intermetallics* **122,** 106813 (2020).
87. A. Shabani, et al., Microstructure and mechanical properties of a multiphase FeCrCuMnNi high-entropy alloy. *J. Mater. Eng. Perform.* **28,** 2388-2398 (2019).
88. S. Elkatatny, et al., Effect of Al content and cold rolling on the microstructure and mechanical properties of $Al_5Cr_{12}Fe_{35}Mn_{28}Ni_{20}$ high-entropy alloy. *Mater. Sci. Eng. A* **759,** 380-390 (2019).
89. T. Zhang, et al., Microstructure and mechanical properties of $Fe_xCoCrNiMn$ high-entropy alloys. *J. of Mater. Sci. Technol.* **35,** 2331-2335 (2019).
90. S. S. Nene, et al., Corrosion-resistant high entropy alloy with high strength and ductility. *Scr. Mater.* **166,** 168-172 (2019).
91. H. Zhang, et al., A novel $FeCoNiCr_{0.2}Si_{0.2}$ high entropy alloy with an excellent balance of mechanical and soft magnetic properties. *J. Magn. Magn. Mater.* **478,** 116-121 (2019).
92. M. Wu, et al., The effects of carbon on the phase stability and mechanical properties of heat-treated FeNiMnCrAl high entropy alloys. *Mater. Sci. Eng. A* **748,** 59-73 (2019).
93. U. Sunkari, et al., Effect of prolonged aging on phase evolution and mechanical properties of intermetallic strengthened $CoCrFeNi_{2.1}Nb_x$ high entropy alloys. *Mater. Lett.* **248,** 119-122 (2019).
94. J. Wang, et al., A novel strategy for enhancing mechanical performance of $Al_{0.5}CoCrFeNi$ high-entropy alloy via high magnetic field. *Mater. Lett.* **240,** 250-252 (2019).
95. Y. Ma, et al., Coherent precipitation and strengthening in a dual-phase $AlNi_2Co_2Fe_{1.5}Cr_{1.5}$ high-entropy alloy. *Mater. Sci. Eng. A* **764,** 138241 (2019).
96. W. Wu, et al., Altered microstructural evolution and mechanical properties of $CoCrFeNiMo_{0.15}$ high-entropy alloy by cryogenic rolling. *Mater. Sci. Eng. A* **759,** 574-582 (2019).

97. Y. Tong, et al., Outstanding tensile properties of a precipitation-strengthened $FeCoNiCrTi_{0.2}$ high-entropy alloy at room and cryogenic temperatures. *Acta Mater.* **165,** 228-240 (2019).
98. X. Yang, et al., Additively manufactured fine grained $Ni_6Cr_4WFe_9Ti$ high entropy alloys with high strength and ductility. *Mater. Sci. Eng. A* **767,** 138394 (2019).
99. Y. H. Jo, et al., Cryogenic-temperature fracture toughness analysis of non-equi-atomic $V_{10}Cr_{10}Fe_{45}Co_{20}Ni_{15}$ high-entropy alloy. *J. Alloy. Compd.* **809,** 151864 (2019).
100. L. Jiang, et al., A promising $CoFeNi_2V_{0.5}Mo_{0.2}$ high entropy alloy with exceptional ductility. *Scr. Mater.* **165,** 128-133 (2019).
101. J. Chen, et al., Effect of C content on microstructure and tensile properties of as-cast CoCrFeMnNi high entropy alloy. *Mater. Chem. Phys.* **210,** 136-145 (2018).
102. L. Zhang, et al., Precipitation-hardened high entropy alloys with excellent tensile properties. *Mater. Sci. Eng. A* **732,** 186-191 (2018).
103. X. W. Liu, et al., The role of carbon in grain refinement of cast CrFeCoNi high-entropy alloys. *Metall. Mater. Tran. A.* **49,** 2151-2160 (2018).
104. Z. Li, & D. Raabe., Influence of compositional inhomogeneity on mechanical behavior of an interstitial dual-phase high-entropy alloy. *Mater. Chem. Phys.* **210,** 29-36 (2018).
105. L. Zhang, et al., The microstructure and high-temperature properties of novel nano precipitation-hardened face centered cubic high-entropy superalloys. *Scr. Mater.* **146,** 226-230 (2018).
106. S. K. Wong, et al., Microstructures and properties of $Al_{0.3}CoCrFeNiMn_x$ high-entropy alloys. *Mater. Chem. Phys.* **210,** 146-151 (2018).
107. Z. Fu, et al., Influence of heat treatment on microstructure, mechanical behavior, and soft magnetic properties in an fcc-based $Fe_{29}Co_{28}Ni_{29}Cu_7Ti_7$ high-entropy alloy. *J. Mater. Res.* **33,** 2214-2222 (2018).
108. Z. G. Zhu, et al., Annealing effect on the phase stability and mechanical properties of $(FeNiCrMn)_{(100-x)}Co_x$ high entropy alloys. *J. Alloy. Compd.* **695,** 2945-2950 (2017).
109. W. Huo, et al., Microstructure and mechanical properties of $CoCrFeNiZr_x$ eutectic high-entropy alloys. *Mater. Des.* **134,** 226-233 (2017).
110. M. Klimova, et al., Microstructure and mechanical properties evolution of the Al, C-containing CoCrFeNiMn-type high-entropy alloy during cold rolling. *Materials.* **11,** 53 (2017).
111. P. Li, A. Wang, & C. T. Liu., Composition dependence of structure, physical and mechanical properties of $FeCoNi(MnAl)_x$ high entropy alloys. *Intermetallics* **87,** 21-26 (2017).
112. G. Laplanche, et al., Reasons for the superior mechanical properties of medium-entropy CrCoNi compared to high-entropy CrMnFeCoNi. *Acta Mater.* **128,** 292-303 (2017).
113. Z. Wang, et al., The effect of carbon on the microstructures, mechanical properties, and deformation mechanisms of thermo-mechanically treated $Fe_{40.4}Ni_{11.3}Mn_{34.8}Al_{7.5}Cr_6$ high entropy alloys. *Acta Mater.* **126,** 346-360 (2017).
114. Z. Y. Rao, et al., Affordable FeCrNiMnCu high entropy alloys with excellent comprehensive tensile properties. *Intermetallics* **77,** 23-33 (2016).
115. Z. G. Zhu, et al., Compositional dependence of phase formation and mechanical properties in three CoCrFeNi-(Mn/Al/Cu) high entropy alloys. *Intermetallics* **79,** 1-11 (2016).
116. Z. Wang, et al., The effect of interstitial carbon on the mechanical properties and dislocation substructure evolution in $Fe_{40.4}Ni_{11.3}Mn_{34.8}Al_{7.5}Cr_6$ high entropy alloys. *Acta Mater.* **120,** 228-239 (2016).
117. J. Y. He, et al., A precipitation-hardened high-entropy alloy with outstanding tensile properties. *Acta Mater.* **102,** 187-196 (2016).
118. Z. Wang, et al., Effect of Ti content on the microstructure and mechanical behavior of $(Fe_{36}Ni_{18}Mn_{33}Al_{13})_{100-x}Ti_x$ high entropy alloys. *Intermetallics* **75,** 79-87 (2016).

119. C. Chen, et al., Microstructure and mechanical properties of $Al_{20-x}Cr_{20+0.5x}Fe_{20}Co_{20}Ni_{20+0.5x}$ high entropy alloys. *J. Alloy. Compd.* **659,** 279-287 (2016).
120. W. H. Liu, et al., Effects of Nb additions on the microstructure and mechanical property of CoCrFeNi high-entropy alloys. *Intermetallics* **60,** 1-8 (2015).
121. Z. Wang, et al., Effect of cold rolling on the microstructure and mechanical properties of $Al_{0.25}CoCrFe_{1.25}Ni_{1.25}$ high-entropy alloy. *Mater. Sci. Eng. A* **645,** 163-169 (2015).
122. S. G. Ma, et al., Microstructural features and tensile behaviors of the $Al_{0.5}CrCuFeNi_2$ high-entropy alloys by cold rolling and subsequent annealing. *Mater. Des.* **88,** 1057-1062 (2015).
123. C. Ng, et al., Phase stability and tensile properties of Co-free $Al_{0.5}CrCuFeNi_2$ high-entropy alloys. *J. Alloy. Compd.* **584,** 530-537 (2014).
124. C. C. Tasan, et al., Composition dependence of phase stability, deformation mechanisms, and mechanical properties of the CoCrFeMnNi high-entropy alloy system. *JOM.* **66,** 1993-2001 (2014).
125. J. Y. He, et al., Effects of Al addition on structural evolution and tensile properties of the FeCoNiCrMn high-entropy alloy system. *Acta Mater.* **62,** 105-113 (2014).
126. L. Liu, et al., Microstructure and tensile properties of $FeMnNiCuCoSn_x$ high entropy alloys. *Mater. Des.* **44,** 223-227 (2013).
127. A. Gali, & E. P. George., Tensile properties of high-and medium-entropy alloys. *Intermetallics* **39,** 74-78 (2013).
128. T. Zuo, et al., Processing effects on the magnetic and mechanical properties of $FeCoNiAl_{0.2}Si_{0.2}$ high entropy alloy. *Inter. J. Min., Met. Mater.* **20,** 549-555 (2013).
129. H. M. Daoud, et al., Microstructure and tensile behavior of $Al_8Co_{17}Cr_{17}Cu_8Fe_{17}Ni_{33}$ (at.%) high-entropy alloy. *JOM.* **65,** 1805-1814 (2013).
130. A. V. Kuznetsov, et al., Tensile properties of an AlCrCuNiFeCo high-entropy alloy in as-cast and wrought conditions. *Mater. Sci. Eng. A* **533,** 107-118 (2012).
131. Y. Zhang, & W. J. Peng., Microstructural control and properties optimization of high-entrop alloys. *Proce. Eng.* **27,** 1169-1178 (2012).
132. F. Wang, et al., Tensile and compressive mechanical behavior of a $CoCrCuFeNiAl_{0.5}$ high entropy alloy. *Int. J. Modern Phys. B.* **23,** 1254-1259 (2009).
133. T. T. Shun, & Y. C. Du., Microstructure and tensile behaviors of FCC $Al_{0.3}CoCrFeNi$ high entropy alloy. *J. Alloy. Compd.* **479,** 157-160 (2009).
134. L. Feng, et al., Tensile properties and molecular dynamics simulation of $FeCrMnAl_xCu$ high-entropy alloys. *Vacuum.* **238,** 114219 (2025).
135. C. Peng, et al., Dual-phase lamellar structure achieving high strength and large ductility in a novel Co-free eutectic high-entropy alloy. *Intermetallics.* **179,** 108669 (2025).
136. J. Q. Liu, et al., Superior strength-ductility synergy in novel as-cast $L1_2$-type HEI $Ni_{50}Co_{25}Cr_{9.5}Al_9Ti_5Ta_{1.5}$ with hierarchical core-shell structure. *Acta Mater.* **288,** 120811 (2025).
137. Q. Liu, et al., Designing novel AlCoCrNi eutectic high entropy alloys. *J. Alloy. Compd.* **904,** 163775 (2022).
138. H. Wu, et al., A cost-effective eutectic high entropy alloy with an excellent strength–ductility combination designed by VEC criterion. *J. Mater. Res. Technol.* **19,** 1759-1765 (2022).
139. Y. Jia, et al., Enhancing the yield strength of Ni–Co–Cr–Fe–Al as-cast hypoeutectic high-entropy alloys by introducing γ' precipitation. *Mater. Sci. Eng. A* **858,** 144190 (2022).
140. Z. Yang, et al., Enhancing the mechanical properties of casting eutectic high entropy alloys with Mo addition. *Appl. Phys. A.* **125,** 1-6 (2019).
141. L. Ma, et al., Microstructure and mechanical property of $Al_{56-x}Co_{24}Cr_{20}Ni_x$ eutectic high-entropy alloys with an ordered FCC/BCT phase structure. *J. Alloy. Compd.* **936,** 168194 (2023).

142. L. Zhang, et al., Eutectic high entropy alloys containing B and Si with excellent mechanical properties in annealing. *Mater. Sci. Eng. A* **856,** 143994 (2022).
143. H. Jiang, et al., Tensile deformation behavior and mechanical properties of a bulk cast $Al_{0.9}CoFeNi_2$ eutectic high-entropy alloy. *J. Mater. Sci. Technol.* **61,** 119-124 (2021).
144. P. J. Shi, et al., A precipitate-free AlCoFeNi eutectic high-entropy alloy with strong strain hardening. *J. Mater. Sci. Technol.* **89,** 88-96 (2021).
145. X. Jin, et al., A new $CrFeNi_2Al$ eutectic high entropy alloy system with excellent mechanical properties. *J. Alloy. Compd.* **770,** 655-661 (2019).
146. Q. Wu, et al., A casting eutectic high entropy alloy with superior strength–ductility combination. *Mater Lett.* **253,** 268-271 (2019).
147. X. Jin, et al., Non-monotonic variation of structural and tensile properties with Cr content in $AlCoCr_xFeNi_2$ high entropy alloys. *J. Alloy. Compd.* **798,** 243-248 (2019).
148. X. Jin, et al., A novel $Fe_{20}Co_{20}Ni_{41}Al_{19}$ eutectic high entropy alloy with excellent tensile properties. *Mater. Lett.* **216,** 144-146 (2018).
149. Y. Lu, et al., Directly cast bulk eutectic and near-eutectic high entropy alloys with balanced strength and ductility in a wide temperature range. *Acta Mater.* **124,** 143-150 (2017).
150. X. Gao, et al., Microstructural origins of high strength and high ductility in an $AlCoCrFeNi_{2.1}$ eutectic high-entropy alloy. *Acta Mater.* **141,** 59-66 (2017).
151. Y. Lu, et al., A promising new class of high-temperature alloys: eutectic high-entropy alloys. *Sci. Rep.* **4,** 6200 (2014).
152. Y. Liao, & I. Baker., Microstructure and room-temperature mechanical properties of $Fe_{30}Ni_{20}Mn_{35}Al_{15}$. *Mater. Charact.* **59,** 1546-1549 (2008).
153. X. Jin, et al., A new pseudo binary strategy to design eutectic high entropy alloys using mixing enthalpy and valence electron concentration. *Mater Des.* **143,** 49-55 (2018).
154. T. Bhattacharjee, et al., Effect of low temperature on tensile properties of $AlCoCrFeNi_{2.1}$ eutectic high entropy alloy. *Mater. Chem. Phys*. **210,** 207-212 (2018).
155. H. Li, et al., High tensile strength and superelasticity of directionally solidified $Ti_{30}Ni_{30}Fe_{10}Hf_{10}Nb_{20}$ eutectic high entropy alloy. *Acta Metall. Sinica*. **35,** 1583-1590 (2022).
156. Y. Dong, et al., Microstructure and mechanical properties of $AlCo_xCrFeNi_{3-x}$ eutectic high-entropy-alloy system. *J. Alloy. Compd.* **823,** 153886 (2020).
157. X. Jin, et al., Enhanced strength and ductility of $Al_{0.9}CoCrNi_{2.1}$ eutectic high entropy alloy by thermomechanical processing. *Materialia* **10**, 100639 (2020).
158. X. Liu, et al., Design of directly cast $Ni_{49}Fe_{20}Al_{17}V_6Co_4Cr_4$ eutectic high-entropy alloy with outstanding mechanical performance. *Mater. Sci. Eng. A* **912,** 146991 (2024).
159. D. Chung, et al., Hetero-deformation promoted strengthening and toughening in BCC rich eutectic and near eutectic high entropy alloys. *J. Mater. Sci. Technol*. **146,** 1-9 (2023).
160. J. Zheng, et al., Effect of Ti Addition on Microstructure Evolution and Mechanical Properties of $Al_{18}Co_{13}Cr_{10}Fe_{14}Ni_{45}$ Eutectic High-Entropy Alloys. *Acta Metall. Sinica*. **36,** 1493-1501 (2023).
161. X. Wang, et al., Design of novel AlCoFeNiV high-entropy alloys with high-strength and high-ductility. *Mater. Charact*. **203,** 113059 (2023).
162. G. Talluri, et al., Designing a eutectic multi-principal element alloy for strength–ductility synergy. *J. Alloy. Compd.* **976,** 173278 (2024).
163. X. Yang, et al., Tensile properties and strengthening mechanisms of eutectic high-entropy alloys induced by heterostructure. *Mater. Charact*. **208,** 113464 (2024).

164. X. Jin, et al., Exploring multicomponent eutectic alloys along an univariant eutectic line. *Mater. Sci. Eng. A* **877,** 145136 (2023).

165. X. Liu, et al., Enhancing the yield strength of casting eutectic high-entropy alloys via coherent precipitates. *Metall. Mater. Tran. A.* **54,** 4620-4624 (2023).

166. N. Xu, et al., Novel casting CoCrNiAl eutectic high entropy alloys with high strength and good ductility. *OAE Publishing Inc.* (2023).

167. H. Jiang, et al., Effects of chromium on the microstructures and mechanical properties of $AlCoCr_xFeNi_{2.1}$ eutectic high entropy alloys. *Acta Metall. Sinica.* **34,** 1565-1573 (2021).

168. Z. Mao, et al., Understanding the yield strength difference in dual-phase eutectic high-entropy alloys. *Mater. Sci. Eng. A* **867,** 144725 (2023).

169. Q. Zhao, et al., Hydrogen induced cracking behavior of the dual-phase $Co_{30}Cr_{10}Fe_{10}Al_{18}Ni_{30}Mo_2$ eutectic high entropy alloy. *Int. J. Hydrogen Energy* **50,** 134-147 (2024).

170. X. Liu, et al., Novel Ni-Co-Cr-Al eutectic high-entropy superalloys with superb mechanical properties. *J. Alloy. Compd.* **1004,** 175987 (2024).

171. C. Su, et al., Microstructure and mechanical property of the novel $Al_{17}Cr_{17}Co_{33}Ni_{33}$ eutectic medium entropy alloy fabricated by powder plasma arc additive manufacturing. *Mater. Lett.* **361,** 136074 (2024).

172. W. Jiao, et al., Hot deformation characteristics and microstructure evolution of $Al_{20}Co_{36}Cr_4Fe_4Ni_{36}$ eutectic high entropy alloy. *Mater. Charact.* **204,** 113180 (2023).

173. X. Y. Zhu, et al., Achieving enhanced mechanical performance in a duplex eutectic $Ni_{49}Fe_{20}Al_{17}Cr_8V_6$ high-entropy alloy with heterogeneous structure. *Intermetallics* **183**, 108805 (2025).

174. C. Peng, et al., Dual-phase lamellar structure achieving high strength and large ductility in a novel Co-free eutectic high-entropy alloy. *Intermetallics.* **179,** 108669 (2025).

175. J. Zhang, et al., Enhance the mechanical properties of $CoCrFeNi(TiC)_{0.2}$ eutectic high-entropy alloy through carbide precipitation and partial recrystallization. *Mater. Sci. Eng. A* **937**, 148438 (2025).

176. X. Zhang, et al., The evolution of eutectic microstructure and mechanical properties of $Al_xCoCrFeNi_{2.1}$ high-entropy alloys. *J. Mater. Res.* **37,** 2082-2092 (2022).

177. M. S. Bijnavandi, A. Ghaderi, & K. Dehghani., Effect of cold rolling on the microstructure rvolution, mechanical, and corrosion properties of $AlCoCrFeNi_{2.4}$ high-entropy alloy. *J. Mater. Eng. Perform.* **33,** 1685-1692 (2024).

178. G. N. Ji, et al., Microstructure and mechanical properties of $Ni_xFeCoCrAl$ high-entropy alloys. *Mater. Today Commus.* **32,** 103919 (2022).

179. J. Liu, et al., Microstructure, mechanical and corrosion properties of Co-and Cu-free $Al_xCrFeNi_{2.5}Mo_{1-x}$ high entropy alloys. *Intermetallics* **153,** 107775 (2023).

180. X. Chen, et al., Influences of Ti additions on the microstructure and tensile properties of $AlCoCrFeNi_{2.1}$ eutectic high entropy alloy. *Intermetallics* **128,** 107024 (2021).

181. Q. W. Tian, et al., Effect of Ni content on the phase formation, tensile properties and deformation mechanisms of the Ni-rich $AlCoCrFeNi_x$ (x= 2, 3, 4) high entropy alloys. *Mater. Charact.***176,** 111148 (2021).

182. L. Zhang, Y. Zhang. Tensile properties and impact toughness of $AlCo_xCrFeNi_{3.1-x}$ (x= 0.4, 1) high-entropy alloys. *Frontiers in Mater.* **7,** 92 (2020).

183. Y. Li, et al., Effect of element V on the as-cast microstructure and mechanical properties of $Al_{0.4}Co_{0.5}V_xFeNi$ high entropy alloys. *J. Alloy. Compd.* **911,** 165043 (2022).

184. T. Han, et al., Refined microstructure and enhanced mechanical properties of $AlCrFe_2Ni_2$ medium entropy alloy produced via laser remelting. *J. Mater. Sci. Technol.* **99,** 18-27 (2022).

185. X. Jin, et al., Back stress strengthening dual-phase $AlCoCr_2FeNi_2$ high entropy alloy with outstanding tensile properties. *Mater. Sci. Eng. A* **745,** 137-143 (2019).

186. C. Chen, et al., Microstructure, mechanical properties and corrosion resistance of an as-cast fine-structured Cr-Fe-Ni-Al-Si high entropy alloy. *Mater. Today Commu.* **35,** 105523 (2023).
187. X. Li, et al., Microstructure, mechanical properties and corrosion resistance of an as-cast fine-structure Cr-Fe-Ni-Al-Si high entropy alloy with Mo addition. *Mater. Today Commu.* **35,** 106020 (2023).
188. Y. Li, et al., Microstructure and mechanical properties of $Al_{0.4}Co_{0.5}V_{0.6}FeNi$ high-entropy alloys processed by homogenization treatment. *Intermetallics* **159,** 107941 (2023).
189. J. Wang, et al., Alloying behavior of W and Mo in the as-cast dual-phase FeNiCrAl multi-component alloys. *J. Alloy. Compd.* **951,** 169951 (2023).
190. H. Yang, et al., Optimize the mechanical properties of $Al_{0.6}CoCrFeNi$ high-entropy alloys by thermo-mechanical processing. *Metals* **12,** 178 (2022).
191. J. Zhang, et al., Effect of Al additions on the microstructures and tensile properties of $Al_xCoCr_3Fe_5Ni$ high entropy alloys. *Mater. Charact.* **175,** 111091 (2021).
192. C. Wei, et al., Evolution of microstructure and mechanical properties of as-cast $Al_xCrFe_2Ni_2$ high-entropy alloys with Al content. *Metall. Mater. Tran. A.* **52,** 1850-1860 (2021).
193. Q. Nie, et al., Microstructures and Mechanical Properties of Multi-component $Al_xCrFe_2Ni_2Mo_{0.2}$ High-Entropy Alloys. *Acta Metall. Sinica.* **33,** 1135-1144 (2020).
194. A. Asabre, et al., Effect of Al, Ti and C additions on Widmanstätten microstructures and mechanical properties of cast $Al_{0.6}CoCrFeNi$ compositionally complex alloys. *Mater. Des.* **184,** 108201 (2019).
195. Y. Dong, et al., A multi-component $AlCrFe_2Ni_2$ alloy with excellent mechanical properties. *Mater. Lett.* **169,** 62-64 (2016).
196. Q. Wang, et al., A cuboidal B2 nanoprecipitation-enhanced body-centered-cubic alloy $Al_{0.7}CoCrFe_2Ni$ with prominent tensile properties. *Scr. Mater.* **120,** 85-89 (2016).
197. L. F. Huang, et al., Vermicular eutectic multi‑principal element alloy with exceptional strength and ductility. *Adv. Sci*. 2501150 (2025).
198. Z. F. Jiang, et al. Mutual FCC-BCC phase transition driving surface homogenization in a novel core-shell medium-entropy alloy after exposure to supercritical water. *Corr. Sci.* **252**, 112971 (2025).
199. Z. F. Jiang, et al., Directly cast fibrous heterostructured $FeNi_{0.9}Cr_{0.5}Al_{0.4}$ high entropy alloy with low-cost and remarkable tensile properties. *Scr. Mater*. **230**, 115421 (2023).
200. Z. Jiang, et al., Lamellar-structured low-cost $FeMn_{0.7}Ni_{0.6}Cr_{0.4}Al_{0.3}$ high entropy alloy with excellent tensile properties. *Vacuum* **209,** 111767 (2023).
201. T. Nguyen, et al., Effect of Al content on microstructure and mechanical properties of as-cast $Al_xFeMnNiCrCu_{0.5}$ high-entropy alloys. *Mater. Sci. Eng. A* **832,** 142495 (2022).
202. C. Chen, et al., Microstructure of and mechanical properties of an as-cast fine-grain dual-phase Fe-based high entropy alloy formed via solid-state phase transformation. *Mater. Sci. Eng. A* **838,** 142779 (2022).
203. W. Zhang, et al., Refinement strengthening, second phase strengthening and spinodal microstructure-induced strength–ductility trade-off in a high-entropy alloy. *Mater. Sci. Eng. A* **847,** 143343 (2022).
204. Z. Ye, et al., Realizing superior strength–ductility combination in dual-phase AlFeCoNiV high-entropy alloy through composition and microstructure design. *Mater. Res. Lett.* **10,** 736-743 (2022).
205. L. Bai, et al., Effect of carbon on microstructure and mechanical properties of $Fe_{36}Mn_{36}Ni_9Cr_9Al_{10}$ high-entropy alloys. *Mater. Sci. Technol.* **36,** 1851-1860 (2020).
206. M. Wu, & I. Baker. High strength and high ductility in a novel $Fe_{40.2}Ni_{11.3}Mn_{30}Al_{7.5}Cr_{11}$ multiphase high entropy alloy. *J. Alloy. Compd.* **820,** 153181 (2020).
207. Gao Q, W. et al., Exceptional strength-ductility synergy in a casting multi-principal element alloy with a hierarchically heterogeneous structure. *Mater. Today* **81**, 70-83 (2024).
